# The SUMO protease SENP3 regulates mitochondrial autophagy mediated by Fis1

**DOI:** 10.15252/embr.201948754

**Published:** 2022-01-07

**Authors:** Emily Waters, Kevin A Wilkinson, Amy L Harding, Ruth E Carmichael, Darren Robinson, Helen E Colley, Chun Guo

**Affiliations:** ^1^ School of Biosciences University of Sheffield Sheffield UK; ^2^ School of Biochemistry University of Bristol Bristol UK; ^3^ School of Clinical Dentistry University of Sheffield Sheffield UK

**Keywords:** Fis1, mitophagy, organellar stress, SENP3, SUMO, Autophagy & Cell Death, Post-translational Modifications & Proteolysis

## Abstract

Mitochondria are unavoidably subject to organellar stress resulting from exposure to a range of reactive molecular species. Consequently, cells operate a poorly understood quality control programme of mitophagy to facilitate elimination of dysfunctional mitochondria. Here, we used a model stressor, deferiprone (DFP), to investigate the molecular basis for stress‐induced mitophagy. We show that mitochondrial fission 1 protein (Fis1) is required for DFP‐induced mitophagy and that Fis1 is SUMOylated at K149, an amino acid residue critical for Fis1 mitochondrial localization. We find that DFP treatment leads to the stabilization of the SUMO protease SENP3, which is mediated by downregulation of the E3 ubiquitin (Ub) ligase CHIP. SENP3 is responsible for Fis1 deSUMOylation and depletion of SENP3 abolishes DFP‐induced mitophagy. Furthermore, preventing Fis1 SUMOylation by conservative K149R mutation enhances Fis1 mitochondrial localization. Critically, expressing a Fis1 K149R mutant restores DFP‐induced mitophagy in SENP3‐depleted cells. Thus, we propose a model in which SENP3‐mediated deSUMOylation facilitates Fis1 mitochondrial localization to underpin stress‐induced mitophagy.

## Graphical abstract

SENP3‐mediated deSUMO‐2/3‐ylation of Fis1 promotes mitophagy induced by deferiprone (DFP).

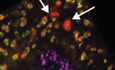

## Introduction

Accurate and proper degradation of dysfunctional mitochondria by mitophagy is essential for maintaining control over mitochondrial quality and quantity. However, mitophagic mechanisms, and their regulation, remain unclear (Green *et al*, 2011). Degradation of dysfunctional mitochondria occurs via a poorly understood quality control programme involving fission of dysfunctional organelles (Twig *et al*, 2008), their accumulation in autophagosomes with elevated levels of LC3 lipidation (Jimenez‐Orgaz *et al*, 2018) and subsequent lysosomal fusion resulting in degradation (Sun *et al*, 2015).

Accumulation of dysfunctional mitochondria is associated with both ageing (Green *et al*, 2011; Lopez‐Otin *et al*, 2013; Biala *et al*, 2015; Gonzalez‐Freire *et al*, 2015) and age‐related diseases (Lionaki *et al*, 2015; Quiros *et al*, 2015; Wong & Holzbaur, 2015). Mitochondrial fission ensures efficient mitophagy (Twig *et al*, 2008; Archer, 2013; Mao *et al*, 2013; Burman *et al*, 2017) and impairment of mitochondrial fission is evident in ageing (Morozov *et al*, 2017) and age‐related diseases (Chen & Chan, 2009; Lionaki *et al*, 2015; Zhang *et al*, 2016). Mitochondrial fission mainly depends on the GTPase dynamin‐related protein 1 (Drp1) (Frank *et al*, 2001), which is recruited to the mitochondrial surface and acts by engaging with specific mitochondrial docking proteins such as Mff, MID49, MID51 and probably Fis1 in mammalian cells (Loson *et al*, 2013). However, Drp1 itself does not appear to be essential for mitophagy (Mendl *et al*, 2011; Song *et al*, 2015; Yamashita *et al*, 2016).

More recently, evidence has emerged for a major role of Fis1 in the disposal of defective mitochondria induced by stressors, including antimycin A, paraquat and deferiprone (DFP), which regulates the formation of reactive oxygen species (Shen *et al*, 2014; Yamano *et al*, 2014; Rojansky *et al*, 2016). Furthermore, Fis1 has been identified, although not validated, in a proteomic screen as a target for post‐translational modification (PTM) by the Small Ubiquitin‐like MOdifier protein (SUMO) *in vivo* (Tirard *et al*, 2012).

SUMOylation involves the reversible covalent conjugation of SUMO to specific lysine(s) in target proteins (Boddy *et al*, 1996; Shen *et al*, 1996; Kamitani *et al*, 1997). Three distinct *SUMO* gene products in mammals are validated for conjugation: SUMO‐1 shares ~ 50% sequence homology with SUMO‐2 and ‐3, which differ by only three amino acids and are collectively referred to as SUMO‐2/3. SUMO conjugation is highly transient and is reversed by the action of SUMO‐specific proteases (Wilkinson & Henley, 2010; Henley *et al*, 2014). SUMO proteases deconjugate SUMO from SUMOylated proteins in a process termed deSUMOylation (Hickey *et al*, 2012; Nayak & Muller, 2014). The extent of target protein SUMOylation in any given state arises from the balance of the opposing activities of SUMO‐conjugating and ‐deconjugating enzymes. To date, three families of SUMO proteases have been identified: deSUMOylating isopeptidase 1 and 2 (DeSI1 and DeSI2), ubiquitin‐specific protease‐like 1 (USPL1) and sentrin‐specific proteases (SENPs). The largest and best characterized family of SUMO proteases, SENPs, consist of six cysteine proteases (SENP1‐3 and 5‐7), each having a distinct subcellular localization and SUMO isoform substrate preference (Hickey *et al*, 2012; Guo & Henley, 2014).

SENP3 is an essential deSUMOylating enzyme primarily responsible for deconjugating SUMO‐2/3 from modified proteins, as evidenced by the findings that SENP3 depletion results in a significant increase in global SUMO‐2/3‐ylation in mammalian cells (Haindl *et al*, 2008; Fanis *et al*, 2012; Guo *et al*, 2013; Luo *et al*, 2017). Although SENP3 is known to play key roles in many processes such as inflammation (Huang *et al*, 2011), stem cell differentiation (Nayak *et al*, 2014), ribosome biogenesis (Raman *et al*, 2016) and cell stress responses (Huang *et al*, 2009; Yan *et al*, 2010; Guo *et al*, 2013), specific targets and physiological roles for this enzyme are largely unknown. Previously believed to be an exclusively nuclear protein, our recent work has revealed that, importantly, SENP3 also resides in the cytoplasm, and is required for mitochondrial fission (Guo *et al*, 2013, 2017), a process which ensures mitophagy‐mediated removal of damaged or dysfunctional mitochondria (Twig *et al*, 2008). Moreover, emerging evidence has strongly implicated SENP3 in ageing and age‐related degenerative diseases. For example, *SENP3* gene expression is markedly reduced in brain samples either from healthy aged subjects (Lu *et al*, 2004) or from Alzheimer's disease patients (Weeraratna *et al*, 2007), and SENP3 levels are altered in brain samples from the patients with Down's syndrome (Binda *et al*, 2017), which is associated with premature ageing and is a risk factor for dementia development (Roth *et al*, 1996; Weksler *et al*, 2013). However, the functional consequences and molecular mechanism(s) underlying the changes in SENP3 levels remain undetermined. Furthermore, accumulation of dysfunctional mitochondria is linked with ageing and age‐related degenerative diseases, and this is believed to be due to defects in a mitochondrial quality control programme enforced by mitophagy, suggesting the possible involvement of SENP3 in mitochondrial quality control.

Here, we have investigated the role of SENP3 in mitophagy‐mediated mitochondrial disposal in model (HeLa) cells by investigating the effects of altered SENP3 levels on induced mitophagy and autophagosome formation. Cells were exposed to a model stressor, the iron chelator DFP, which specifically induces mitophagy, but not general autophagy (Allen *et al*, 2013; Sargsyan *et al*, 2015). DFP is used for treatment of iron overload in inherited and degenerative diseases such as Thalassemia major and Parkinson’s disease. We show that levels of the SUMO‐2/3‐specific deSUMOylating enzyme SENP3 are greatly stabilized upon DFP treatment via a pathway that involves the reduction in levels of the E3 ubiquitin (Ub)‐protein ligase CHIP. This stabilization of SENP3 decreases Fis1 SUMO‐2/3‐ylation, enhances Fis1 mitochondrial localization and induces Fis1‐mediated LC3 lipidation and mitochondrial disposal. Taken together, our results reveal a novel mechanism dependent on the presence of SENP3 in co‐ordinated regulation of the SUMOylation status of Fis1 to ensure mitochondrial quality control.

## Results

### DFP‐induced mitophagy is accompanied by decreased SUMO‐2/3 conjugation and increased SENP3 levels

Deferiprone is known to induce Parkin‐independent mitophagy (Allen *et al*, 2013). Consistent with this, we detected induction of autophagy, as evidenced by increased LC3‐II levels (a commonly used marker for LC3 lipidation, and phagophore or autophagosome formation), by DFP in HeLa cells that express little or no Parkin, and the upregulation of LC3 lipidation was confirmed following the inhibition of lysosomes with choloroquine (CQ) (Appendix Fig S1).

**Figure EV1:**
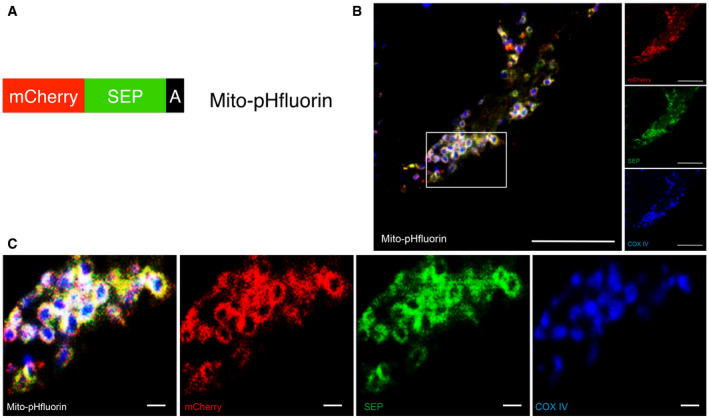
Mito‐pHfluorin outlines the mitochondria Schematic illustrates Mito‐pHfluorin, a tandem‐tagged construct encoding mCherry‐SEP‐A (SEP, Super‐ecliptic pHluorin (a pH‐dependent GFP variant); A, the mitochondrial targeting sequence derived from the ActA protein fused to the C‐terminus of SEP).Mito‐pHfluorin was transfected into HeLa cells for 48 h. Cells were fixed with 4% PFA, and immunocytochemistry against the mitochondrial inner membrane protein COX IV was performed (Red: mCherry; Green: SEP; Blue: COX IV; Scale bar, 10 μm).Magnified views of the boxed area to show the co‐localization of mito‐pHfluorin (outer ring) and COX IV (inner circle) (Scale bar, 1 μm).

**Figure EV2:**
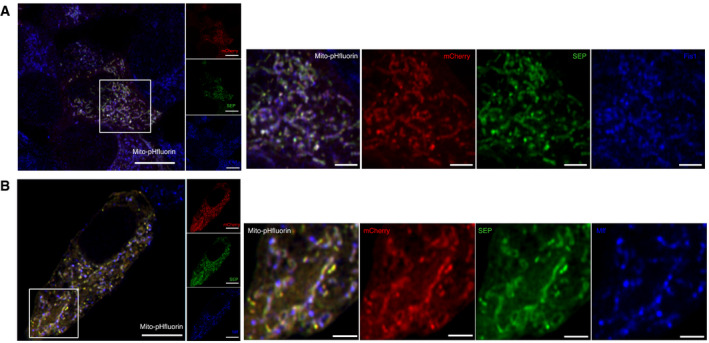
Mito‐pHfluorin is targeted to the mitochondria outer membrane containing Fis1 and Mff Mito‐pHfluorin was transfected into HeLa cells for 48 h. Cells were fixed with 4% PFA, and immunocytochemistry against the mitochondrial outer membrane protein Fis1 was performed (Left panel; Red: mCherry; Green: SEP; Blue: Fis1; Scale bar, 10 μm). Magnified views of the boxed area to show the co‐localization of mito‐pHfluorin and Fis1 (Light panel; Scale bar, 2.5 μm).Mito‐pHfluorin was transfected into HeLa cells for 48 h. Cells were fixed with 4% PFA, and immunocytochemistry against the mitochondrial outer membrane protein Mff was performed (Left panel; Red: mCherry; Green: SEP; Blue: Mff; Scale bar, 10 μm). Magnified views of the boxed area to show the localization of Mff within the mitochondrial outer membrane outlined by mito‐pHfluorin (Light panel; Scale bar, 2.5 μm).

**Figure EV3:**
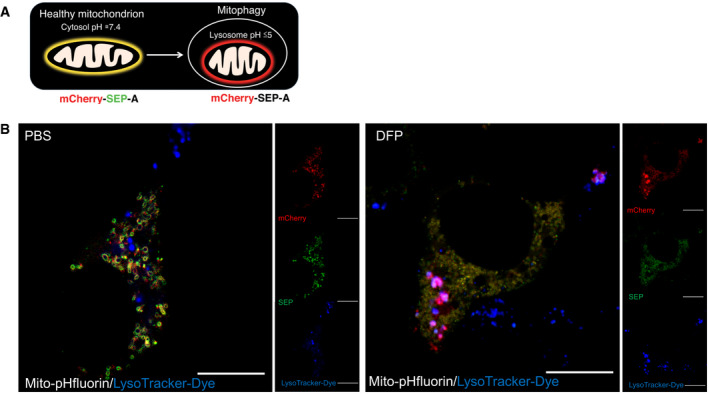
Mito‐pHfluorin is a probe for DFP‐induced mitophagy Schematic illustrates a mitophagy assay using the mito‐pHfluorin construct. Under basal conditions, in green/red merged images mitochondria are labelled as yellow structures while upon mitophagy, mitochondria are sequestered within autolysosomes where fluorescence of SEP is quenched due to the acidic environment and therefore outlined as red structures.Co‐localization of red‐alone puncta detected by mito‐pHfluorin and lysosomes stained by LysoTracker (Red: mCherry; Green: SEP; Blue: lysosomes; Scale bar, 10 μm).

To detect lysosomal‐mediated acidification of engulfed mitochondria, we designed and characterized a novel probe termed mito‐pHfluorin (Fig EV1A). This probe consists of mCherry–SEP (a pH‐sensitive GFP‐variant (Ashby *et al*, 2004)) tagged to the mitochondrial outer membrane targeting sequence of the ActA protein of *Listeria monocytogenes* (Pistor *et al*, 1994). Mito‐pHfluorin delineates mitochondria and surrounds the COX IV‐containing mitochondrial inner membranes (Fig EV1B and C) and co‐localizes with mitochondrial outer membranes containing Fis1 (Fig EV2A) and Mff (Fig EV2B), indicating the targeting of mito‐pHfluorin to the organelle. HeLa cells treated with DFP show increased levels of SEP fluorescence quenching that results in the appearance of red puncta (Fig EV3A). The quenchable dual‐fluorescence mitochondrial markers in DFP‐treated cells were co‐localized with structures stained by LysoTracker (Fig EV3B), suggesting they represent mitochondria which have been engulfed by autolysosomes. Furthermore, by assessing the size of autophagosome structures we established criteria for the use of mito‐pHfluorin in identification of autolysosomes that we use in subsequent figures (see Materials and Methods for details).

**Figure 1:**
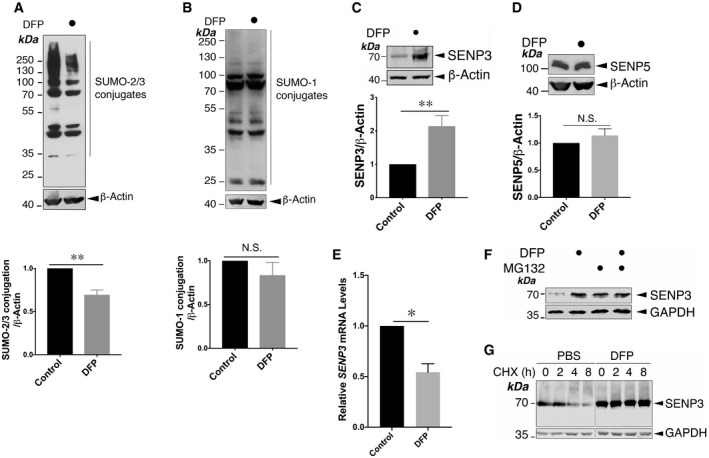
Iron chelation induces protein deSUMO‐2/3‐ylation coinciding with SENP3 stabilization Treatment of HeLa cells with DFP (1 mM for 24 h) reduces the conjugation of SUMO‐2/3 (*n* = 5, biological replicates; **P < 0.01; paired *t*‐test).Treatment of HeLa cells with DFP (1 mM for 24 h) does not change global SUMO‐1‐ylation (*n* = 5, biological replicates; *P* > 0.05; N.S., not statistically significant; paired *t*‐test).Treatment of HeLa cells with DFP (1 mM for 24 h) leads to increased levels of SENP3 (*n* = 8, biological replicates; **P < 0.01; paired *t*‐test).Treatment of HeLa cells with DFP (1 mM for 24 h) does not change levels of SENP5 (*n* = 5, biological replicates; *P* > 0.05; N.S., not statistically significant; paired *t*‐test).Treatment of HeLa cells with DFP (1 mM for 24 h) leads to decreased levels of *SENP3* mRNA (*n* = 3, biological replicates; *P < 0.05; unpaired *t*‐test).Treatment of DFP (1 mM for 24 h)‐treated HeLa cells with 50 μM MG132 for the final 6 h prevents further increase in SENP3 levels induced by DFP.DFP treatment increases SENP3 stability. DFP (1 mM for 24 h)‐treated HeLa cells were treated with cycloheximide (CHX; 50 μg/ml) for the indicated durations in a chase experiment. Lysate samples were separated on different lanes in the same gel and immunoblotted as indicated. Data information: In (A–E) values are presented as mean ± SEM and are normalized to the control value. Source data are available online for this figure.

Interestingly, DFP treatment also led to a significant decrease in global SUMO‐2/3‐ylation (Fig 1A) but not SUMO‐1‐lyation (Fig 1B) in HeLa cells. Moreover, levels of the deSUMOylating enzyme SENP3 (Fig 1C), but not SENP5 (Fig 1D), were increased by DFP. Surprisingly, levels of *SENP3* mRNA were actually significantly reduced by DFP treatment (Fig 1E). However, the DFP‐induced SENP3 increase was prevented by proteasome inhibition (Fig 1F), and SENP3 stability was increased in DFP‐treated cells (Fig 1G). Taken together, these findings suggest that a proteasome‐dependent mechanism underlies increased SENP3 stability upon DFP treatment, leading to the decrease in global SUMO‐2/3‐lyation.

### DFP treatment decreases CHIP levels, most likely through reducing *CHIP* mRNA

**Figure 2:**
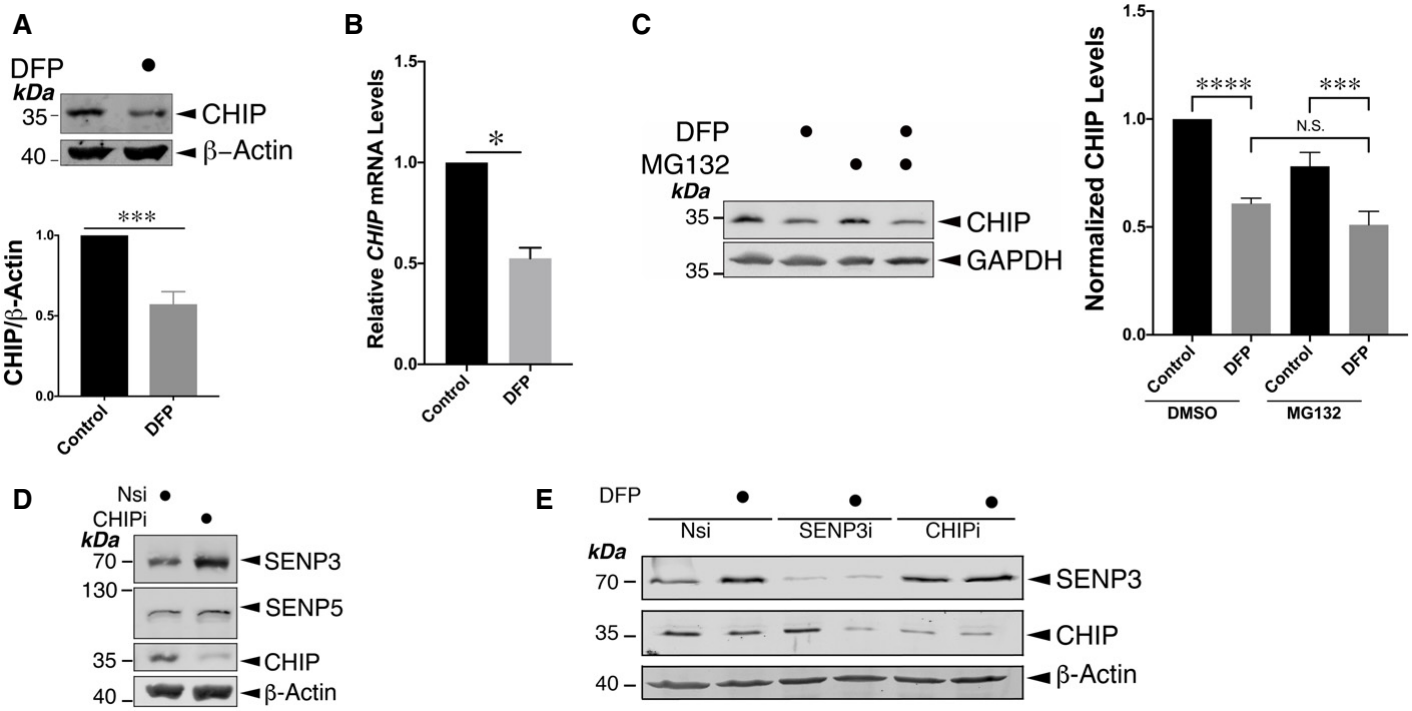
Iron chelation reduces levels of both CHIP and *CHIP* mRNA Treatment of HeLa cells with DFP (1 mM for 24 h) decreases levels of CHIP (*n* = 8, biological replicates; ***P < 0.001; paired *t*‐test).Treatment of HeLa cells with DFP (1 mM for 24 h) leads to decreased levels of *CHIP* mRNA (*n* = 3, biological replicates; *P < 0.05; unpaired *t*‐test).Treatment of DFP (1 mM for 24 h)‐treated HeLa cells with 50 μM MG132 for the final 6 h does not recover reduced levels of CHIP induced by DFP. Histogram shows relative levels of cellular CHIP upon four different conditions (*n* = 8, biological replicates; ****P < 0.0001; ***P < 0.001; repeated measures analysis of variance followed by Tukey's multiple comparisons test).CHIP knockdown increases levels of SENP3 but not SENP5 in HeLa cells. Nsi or CHIPi (Nsi, non‐specific siRNA; CHIPi, CHIP siRNA; concentration, 20 nM) was transfected into HeLa cells for 48 h. Lysate samples were blotted as indicated.CHIP knockdown prevents the DFP‐induced increase in SENP3 levels. Nsi, SENP3i or CHIPi (Nsi, non‐specific siRNA; SENP3i, SENP3 siRNA; CHIPi, CHIP siRNA; siRNA concentration, 20 nM) was transfected into HeLa cells for 48 h. Two days post‐transfection the cells were treated with DFP (1mM) for a further 24 h. Data information: In (A, C, D, E) whole cell lysate samples were blotted as indicated. In (A–C) values are presented as mean ± SEM and are normalized to the control value. Source data are available online for this figure.

Interestingly DFP treatment decreased the levels of the Ub ligase CHIP and reduced *CHIP* mRNA (Fig 2A and B). Moreover, proteasome inhibition did not recover the decreased CHIP levels in DFP‐treated cells (Fig 2C). Furthermore, consistent with previous findings where CHIP was identified as an E3 Ub ligase known to control SENP3 levels under basal conditions (Yan *et al*, 2010), knocking down CHIP in HeLa cells increased the levels of SENP3 but not SENP5 (Fig 2D), and prevented the DFP‐induced increase in SENP3 levels (Fig 2E). Taken together, these findings suggest that the downregulation of *CHIP* gene expression upon DFP treatment underlies decreased CHIP levels, most likely leading to increased SENP3 levels.

### SENP3 is required for mitophagy induced by DFP

**Figure 3:**
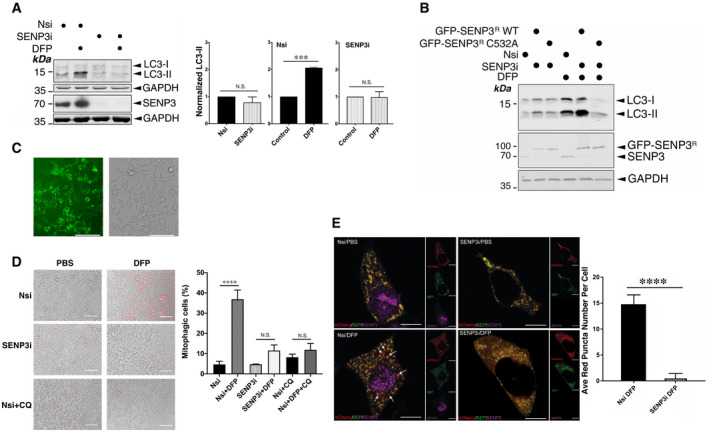
SENP3 is required for mitophagy induced by DFP SENP3 knockdown abolishes the DFP‐induced increase in LC3‐II in HeLa cells. Nsi or SENP3i (I) was transfected into HeLa cells. Two days post‐transfection the cells were treated with DFP for a further 24 h. Whole cell lysate samples were blotted as indicated (Nsi, non‐specific siRNA; SENP3i, SENP3 siRNA; siRNA concentration, 20 nM; DFP, 1 mM for 24 h; values normalized to the control value; *n* = 3, biological replicates; ***P < 0.001; N.S., not significant, *P* > 0.05; paired *t*‐test).Replacement of endogenous SENP3 with GFP‐SENP3^R^ WT, but not GFP‐SENP3^R^ C532A, restores DFP‐induced LC3‐II levels. GFP‐SENP3^R^ WT or SENP3^R^ C532A was expressed in HeLa cells after knockdown of endogenous SENP3 using SENP3i (II). The cells were treated with DFP (1 mM for 24 h) in the presence of CQ (50 μM; for final 12 h of DFP treatment). Whole cell lysate samples were blotted as indicated.Establishment of HeLa cells stably expressing mito‐Keima (Live image of cells grown in a T75 flask; Scale bar, 100 μm).Nsi or SENP3i (II) was transfected into HeLa cells with stable expression of mito‐Keima grown on six‐well plates. Two days post‐transfection the cells were treated with DFP (1 mM; 24 h). Cells transfected with Nsi and treated with CQ (50 μM; 24 h) in the absence or presence of DFP (1 mM; 24 h) were included as a negative control for mitophagy induction. The images show overlaid fluorescence and transmitted light images of unfixed, live cells obtained using an EVOS‐fl inverted LED fluorescence microscope (Red: mitophagic puncta; Scale bar, 100 μm). Histogram shows the percentages of mitophagic cells under six different conditions (*n* = 3, biological replicates; ****P < 0.0001; Ordinary one‐way ANOVA followed by Tukey's multiple comparisons test).SENP3 knockdown abolishes DFP‐induced mitophagic autolysosomes. Nsi or SENP3i (I) together with mito‐pHfluorin were transfected into HeLa cells in the absence or presence of DFP (24 h), and were analysed using the mito‐pHfluorin construct 72 h post‐transfection (Red: mCherry‐A or puncta indicating occurrences of mitophagy marked by white arrows; Green: SEP‐A; Yellow: mitochondria labelled by mCherry‐SEP‐A; Magenta: SENP3; Scale bar, 10 μm; *n* = 28 cells per condition from two individual experiments; ****P < 0.0001; unpaired *t*‐test). Data information: In (A), (D) and (E) values are presented as mean ± SEM. Source data are available online for this figure.

Mitophagy‐mediated disposal of dysfunctional mitochondria occurs via a multi‐step process involving two intracellular events: mitochondrial autophagosome formation and subsequent autolysosome formation (Yamashita & Kanki, 2017). To explore if SENP3 is involved in DFP‐induced LC3 lipidation, we examined the effect of RNAi‐mediated knockdown of SENP3 on the expression of the LC3 lipidation marker LC3‐II in HeLa cells treated with DFP. SENP3 knockdown blocked the DFP‐induced increase in LC3‐II expression (Fig 3A and Appendix Fig S2A and B), indicating an essential role for SENP3 in LC3 lipidation. Furthermore, DFP treatment caused LC3‐II induction in SENP3 knockdown cells expressing RNAi‐resistant GFP‐SENP3 wild‐type (WT) but not its catalytically inactive C532A mutant (Fig 3B and Appendix Fig S2C), indicating that the deSUMOylation activity of SENP3 is required for DFP‐induced autophagosome formation.

In contrast to LC3‐II, levels of the LC3‐interacting protein p62 were reduced by DFP treatment, consistent with previous reports (Sargsyan *et al*, 2015; Yamashita *et al*, 2016). Interestingly, however, SENP3 knockdown prevented the DFP‐induced decrease in p62 (Appendix Fig S3), supporting a potential role for p62 in DFP‐induced mitophagy, and a further role for SENP3 in this process.

Next, to investigate if SENP3 is important for mitochondria‐containing autolysosome formation induced by DFP, we examined the role of SENP3 in mitophagic flux using mito‐Keima, a well‐established probe targeting the mitochondrial inner membrane for mitophagy detection (Sun *et al*, 2017), in living HeLa cells treated with DFP. Either SENP3 knockdown or CQ treatment prevented DFP‐induced mitophagic red puncta in HeLa stably expressing mito‐Keima (Fig 3C and D and Appendix Table S1).

**Figure EV4:**
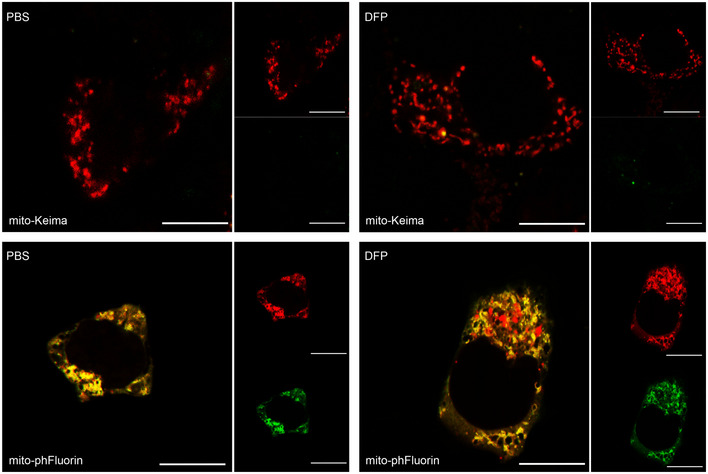
Mito‐pHfluorin is suitable for detecting DFP‐induced mitophagy in fixed HeLa cells Mito‐Keima or mito‐pHfluorin were transfected into HeLa cells for 48 h. Cells were treated with either PBS or DFP (1 mM; 24 h) and fixed with 4% PFA prior to imaging analysis. Images of mito‐Keima under control (PBS) or DFP treatment conditions are shown in the upper panel (Red: Mito‐Keima; Green, residual green fluorescence emission from Mito‐Keima; Scale bar, 10 μm). Images of mito‐pHfluorin under the two conditions are shown in the lower panel. Red: mCherry; Green, SEP; Scale bar, 10 μm. In mito‐pHfluroin‐expressing cells, mitochondria are visualized in the merged images as yellow structures, and red, SEP‐quenched, puncta are present in DFP‐treated cells.

We then sought to reproduce these findings in fixed cells. However, in agreement with previous studies (Sun *et al*, 2017), we found mito‐Keima to be less suitable than mito‐pHfluorin for detection of DFP‐mitophagy under these conditions (Fig EV4). Therefore, we examined mitochondria‐containing autolysosome formation in control and SENP3‐knockdown cells using mito‐pHfluorin. In agreement with our results using mito‐Keima in live cells, SENP3 knockdown significantly reduced the levels of SEP fluorescence quenching after DFP treatment, indicating a critical role for SENP3 in the disposal of dysfunctional mitochondria (Fig 3E). Taken together, these results suggest that a protein deSUMOylation pathway mediated by SENP3 promotes DFP‐induced mitophagy.

### Fis1 is critical for DFP‐induced mitophagy

Since SENP3‐mediated deSUMOylation regulates mitophagy, we next questioned the identity of the specific substrate(s) of its deSUMOylating activity that may mediate this effect. We reasoned that the most likely SENP3 substrates in this pathway are component(s) of the mitochondrial fission machinery. Mitochondrial fission depends on the action of the large GTPase Drp1, a validated SUMO target (Figueroa‐Romero *et al*, 2009; Guo *et al*, 2013; Prudent *et al*, 2015). Drp1 is recruited to the mitochondrial surface and acts by interacting with specific mitochondrial docking proteins such as Mff, MID49, MID51 and possibly Fis1, in mammalian cells (Loson *et al*, 2013). However, Drp1 does not appear to be essential for mitophagy (Yamashita *et al*, 2016; Yamashita & Kanki, 2017), and the role of Drp1 deSUMOylation in DFP‐induced mitophagy is unclear. Levels of LC3‐II induction by DFP were comparable between Drp1 knockdown cells expressing RNAi*‐*resistant YFP*‐*Drp1^R^ WT and their counterparts expressing a non*‐*SUMOylatable mutant Drp1^R^ 4KR (Appendix Fig S4), discounting the possibility of Drp1 deSUMOlyation playing a role in DFP‐induced mitophagy.

**Figure 4:**
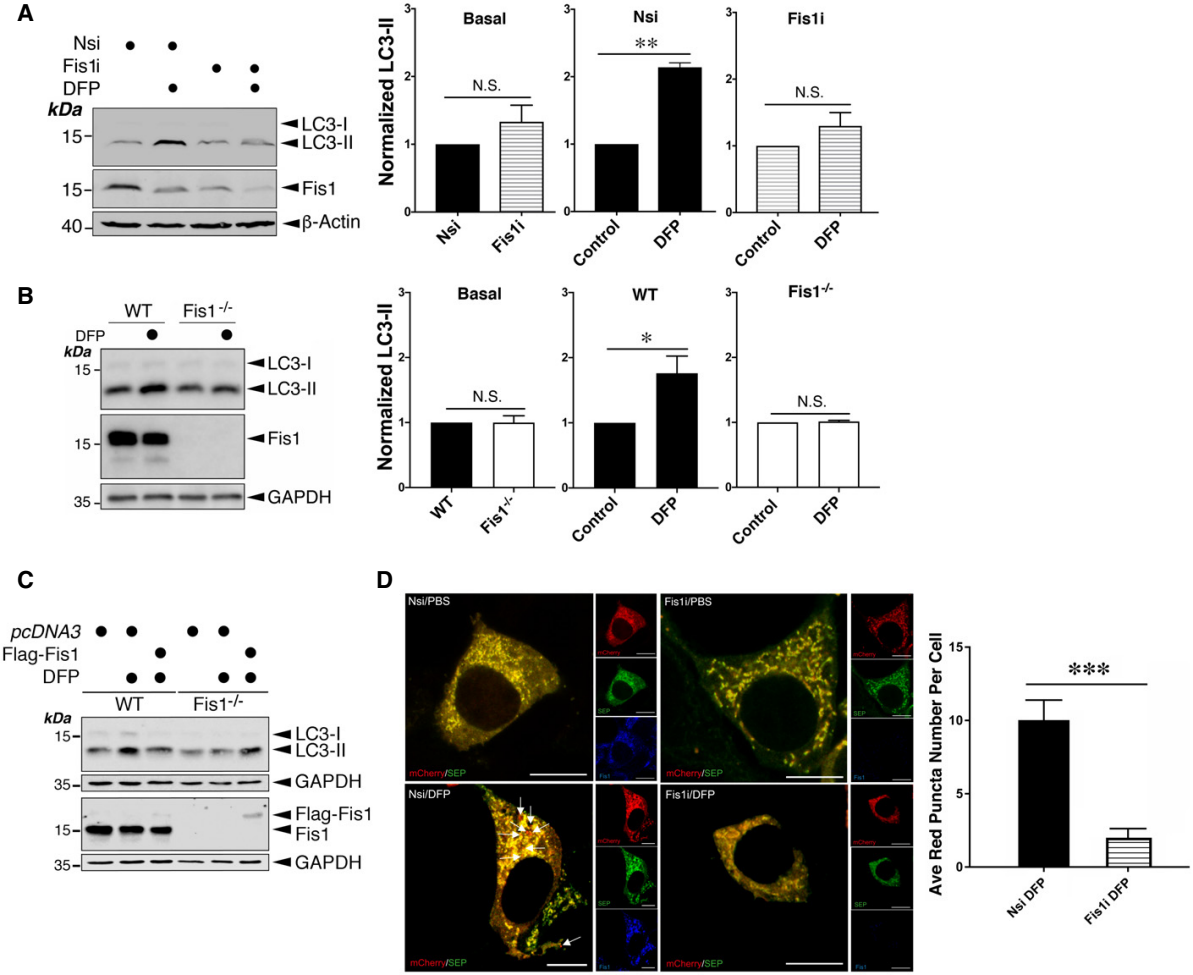
Fis1 is required for DFP‐induced mitophagy Fis1 knockdown abolishes DFP‐induced LC3‐II. Fis1 siRNA was introduced into HeLa cells for 48 h, and the cells were treated with DFP for further 24 h (Nsi, non‐specific siRNA; Fis1i, Fis1 siRNA; siRNA concentration, 20 nM; DFP, 1 mM). Whole cell lysate samples were blotted as indicated. Values are presented as mean ± SEM and are normalized to the control value (*n* = 3, biological replicates; **P < 0.01; paired *t*‐test).Fis1 knockout abolishes DFP‐induced LC3‐II. Fis1^+/+^ or Fis1^−/−^ cells were treated with DFP for 24 h. Whole cell lysate samples were blotted as indicated. Values are presented as mean ± SEM and are normalized to the control value (*n* = 6, biological replicates; *P < 0.05; paired *t*‐test).Expressing exogenous Fis1 restores LC3‐II induction by DFP in Fis1 KO cells. WT or Fis1 KO HeLa cells transfected with *pcDNA3* or Flag‐Fis1 for 48 h were treated with DFP for a further 24 h. Whole cell lysate samples were blotted as indicated.Fis1 knockdown prevents DFP‐induced mitophagic autolysosomes. Nsi or Fis1i together with mito‐pHfluorin were transfected into HeLa cells in the absence or presence of DFP (24 h), which were analysed using the mito‐pHfluorin construct 72 h post‐transfection (Red: mCherry‐A or puncta indicating occurrences of mitophagy marked by white arrows; Green: SEP‐A; Yellow: mitochondria labelled by mCherry‐SEP‐A; Blue: Fis1; Scale bar, 10 μm; *n* = 25 cells per condition from three individual experiments; ***P < 0.001; unpaired *t*‐test). Data information: In (A), (B) and (D) values are presented as mean ± SEM. Source data are available online for this figure.

Of note, evidence has emerged for the critical role of Fis1 in the mitophagy‐mediated disposal of defective mitochondria induced by stressors (Shen *et al*, 2014; Yamano *et al*, 2014; Rojansky *et al*, 2016). In agreement with previous studies where Fis1 is degraded upon mitophagy (Yamano *et al*, 2014), treatment of cells with DFP results in the loss of Fis1 but not *FIS1* mRNA (Fig 4A and Appendix Fig S5). To investigate if Fis1 is involved in DFP‐induced mitochondrial autophagosome formation, we first examined the effect of RNAi‐mediated knockdown of Fis1 on the induction of LC3‐II in HeLa cells treated with DFP. Interestingly, Fis1 knockdown, as well as Fis1 knockout (KO) using CRISPR/Cas9, eliminates LC3‐II induction by DFP in HeLa cells (Fig 4A and B), indicating a critical role for Fis1 in DFP‐induced mitophagic LC3 lipidation. Importantly, LC3‐II becomes inducible in DFP‐treated Fis1 KO cells expressing Flag‐Fis1, further substantiating the role of Fis1 in DFP‐induced mitophagy (Fig 4C), although Fis1 overexpression itself does not result in LC3‐II induction in either WT or Fis1 KO cells under basal conditions (Appendix Fig S6).

We then compared mitochondria‐containing autolysosome formation in fixed control and Fis1 knockdown cells using mito‐pHfluorin. Fis1 depletion significantly reduced the levels of SEP fluorescence quenching induced by DFP, indicating a critical role for Fis1 in the disposal of dysfunctional mitochondria upon induction of iron chelation‐mediated stress (Fig 4D). Taken together, these results indicate that Fis1 is required for DFP‐induced mitophagy.

### Fis1 is a novel SUMO target and is deSUMOylated by SENP3

**Figure 5:**
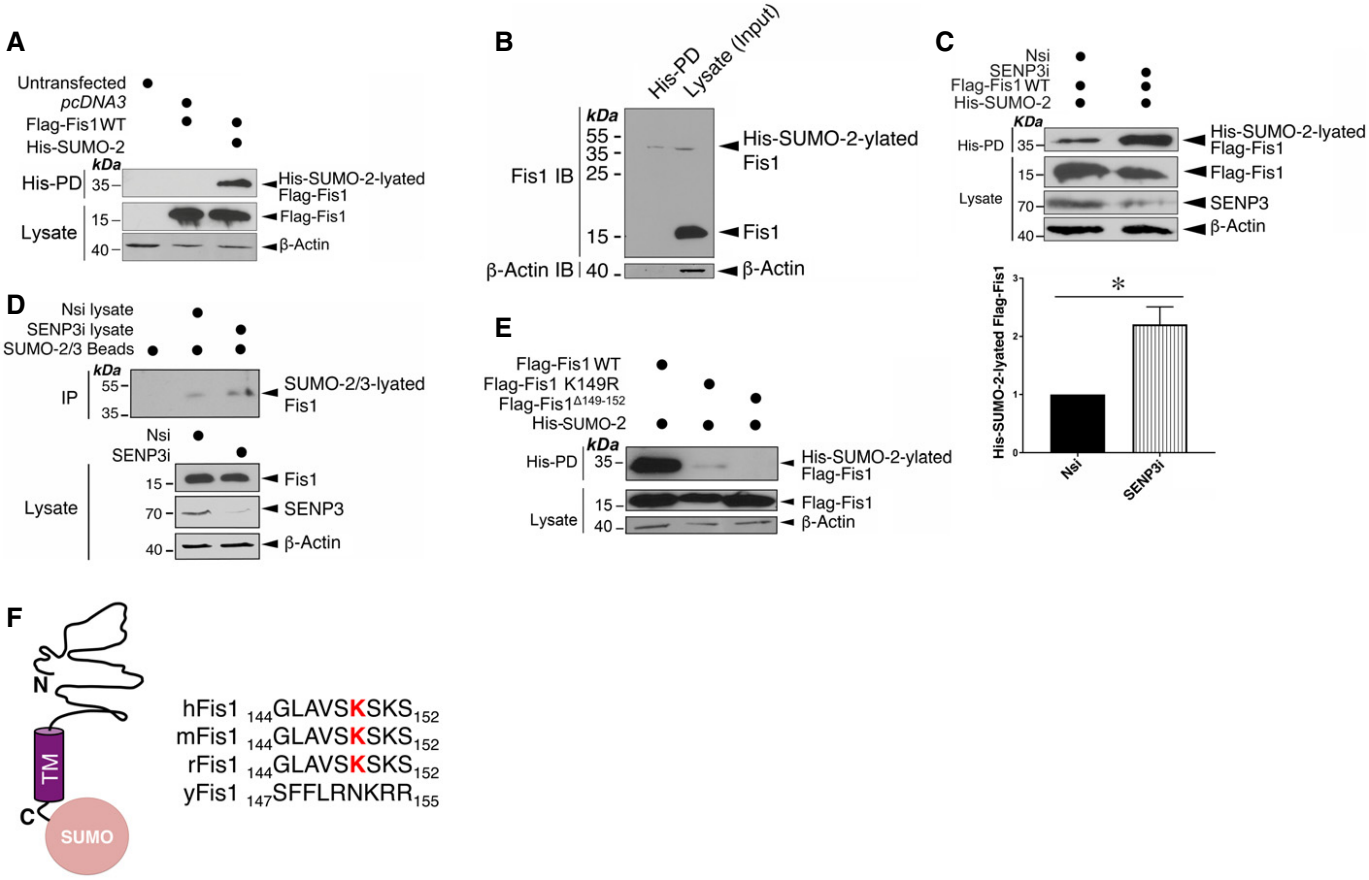
SENP3 mediates deSUMOlyation of Fis1 Fis1 is a *bona fide* target for SUMOylation. Fis1 is a SUMO target in mammalian cells. Flag‐Fis1 together with *pcDNA3* or His‐SUMO‐2 were transfected into HEK293 cells expressing Ubc9 for 48 h. His‐pulldown (His‐PD) and lysate samples were detected by immunoblotting for Flag or β‐actin.Endogenous Fis1 is modified by His‐SUMO‐2 stably expressed in HEK293 cells. His‐pulldown (His‐PD) and lysate samples were blotted as indicated.Flag‐Fis1 is deSUMOylated by SENP3. SENP3 knockdown enhances Fis1 SUMOylation. Nsi or SENP3i was transfected into HEK293 cells expressing Flag‐Fis1, His‐SUMO‐2 and Ubc9 for 48 h. His‐pulldown and lysate samples were detected by immunoblotting for Flag, SENP3 or β‐actin. Values are presented as mean ± SEM (*n* = 3, biological replicates; *P < 0.05; paired *t*‐test).SENP3 knockdown increases endogenous Fis1 SUMO‐2/3‐ylation in HeLa cells. SUMO‐2/3 conjugates were immunoprecipitated (IP) from whole cell lysates prepared from HeLa cells using SUMO‐2/3 affinity beads. IP and lysate samples were blotted as indicated.Amino acids including lysine 149 at the C‐terminus of Fis1 are required for Fis1 SUMOylation. Flag‐Fis1, Flag‐Fis1 K149R mutant or Flag‐Fis1 Δ149‐152 mutant together with His‐SUMO‐2 were transfected into HEK293 cells expressing Ubc9 for 48 h. His‐pulldown and lysate samples were blotted as indicated.Alignment (the right panel) shows amino acid sequences of the C‐terminal tail of Fis1 from the indicated organisms: human (h), mouse (m), rat (r) and *S. cerevisiae* (y); red letters mark the lysine residue required for Fis1 SUMOylation. Schematic (the left panel) illustrates SUMO conjugation at the C‐terminal tail of Fis1 (N, N‐terminus; TM, Transmembrane domain; C, C‐terminus). Source data are available online for this figure.

Since Fis1 has been identified as a potential target for SUMOylation in a proteomics screen for SUMO targets *in vivo* (Tirard *et al*, 2012), we next verified whether Fis1 was in fact a *bona fide* target for SUMOylation. Flag‐Fis1 is efficiently conjugated by His‐SUMO‐2 in HEK293 cells (Fig 5A). Subsequently, under denaturing conditions, we detected conjugation of His‐SUMO‐2 to endogenous Fis1 in HEK293 cells (Fig 5B). Importantly, SENP3 knockdown led to a significant increase in Flag‐Fis1 SUMO‐2‐ylation and in endogenous Fis1 SUMO‐2/3‐ylation (Fig 5C and D) while overexpressing GFP‐SENP3 resulted in a decrease in endogenous Fis1 SUMO‐2/3‐ylation (Appendix Fig S7), indicating that SENP3 directly deSUMOylates Fis1. Taken together, these findings indicate that Fis1 is subject to SUMO‐2/3‐lyation under basal conditions, which can be reversed through the action of the deSUMOylating enzyme, SENP3.

### Fis1 is primarily SUMOylated at lysine 149

To identify which lysine in Fis1 is SUMOylated, we performed site‐directed mutagenesis. Fis1 contains one high‐probability consensus lysine residue (K119) and three high‐probability non‐consensus lysine residues (K67, K149 and K151), predicted by GPS‐SUMO (Zhao *et al*, 2014). While mutation of either the K119 or the K67 to a non‐SUMOylatable arginine (R) did not reduce Fis1 SUMOylation in a HEK293 cell‐based assay under denaturing conditions (Appendix Fig S8A), K149R mutation in Fis1 reduced Fis1 SUMOylation by ~ 90% (Fig 5E). Moreover, the deletion of the final four amino acid residues (^149^ **K**S**K**S^152^) completely eliminated Fis1 SUMOylation (Fig 5E). This finding led us to question if K151 in Fis1 was a minor site for SUMO conjugation. Unexpectedly, K151R mutation does not seem to reduce Fis1 SUMOylation (Appendix Fig S8B), however it is possible that this site becomes a target only in the absence of the nearby K149. Nonetheless, these findings indicate that K149 is the major SUMO acceptor site in Fis1 (Fig 5F).

### The SUMOylation status of Fis1 may act as a key switch to regulate its mitochondrial localization and mitophagy induction

**Figure 6:**
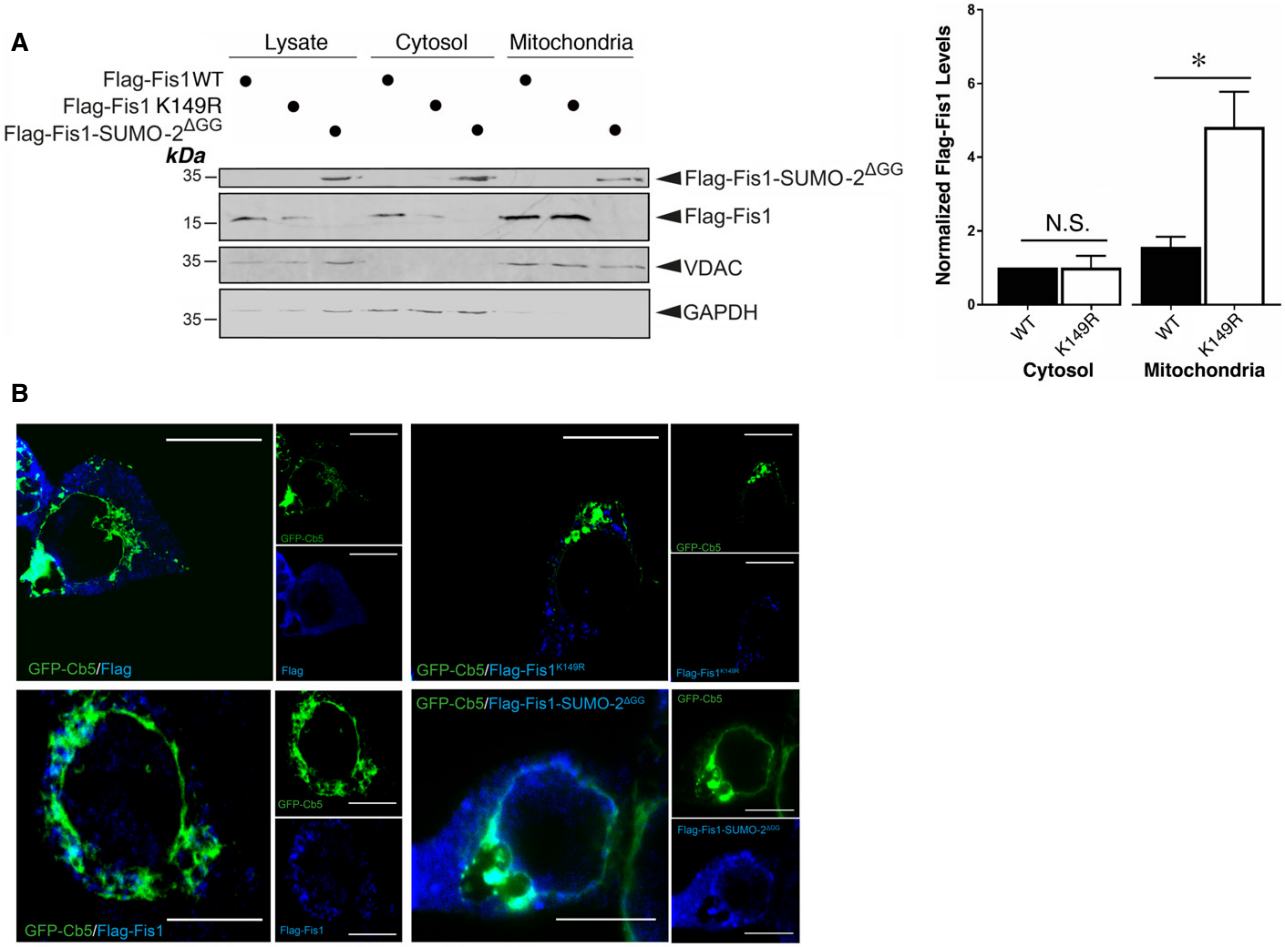
SUMOylation influences Fis1 subcellular distribution The SUMOylation status of Fis1 influences its mitochondrial localization. Flag‐Fis1 WT, Flag‐Fis1 SUMOylation‐deficient mutant K149R or Flag‐Fis1‐SUMO‐2^ΔGG^ was expressed in HeLa cells for 48 h. Whole cell lysates and cytosolic and mitochondrial fractions were prepared and blotted as indicated (the upper panel). Histograms (the right panel) show normalized levels of Flag‐Fis1 WT or Flag‐Fis1 K149R mutant associated with the cytosolic or mitochondrial fraction (Values are presented as mean ± SEM and are normalized to the control value; *n* = 7, biological replicates; *P < 0.05; paired *t*‐test).The SUMOylation status of Fis1 may influence its association with the Endoplasmic Reticulum (ER). Flag‐Fis1 WT, Flag‐Fis1 SUMOylation‐deficient mutant K149R or Flag‐Fis1‐SUMO‐2^ΔGG^ along with GFP‐Cb5 as an ER marker were expressed in HeLa cells for 48 h. Immunocytochemistry was then performed (Green: ER labelled by GFP‐Cb5; Blue: different Flag‐Fis1 forms; Scale bar, 10 μm). Source data are available online for this figure.

Fis1 has been detected in three organelles, mitochondria, peroxisomes and endoplasmic reticulum (ER) (Stojanovski *et al*, 2004; Ji *et al*, 2017). Interestingly, K149 is known to be critical for localizing Fis1 to mitochondria. Non‐conservative mutation of K149 to alanine (A) greatly reduces Fis1 mitochondrial localization (Stojanovski *et al*, 2004; Delille & Schrader, 2008) while Fis1 with the conservative K149R mutation is localized to mitochondria (Alirol *et al*, 2006). However, whether the K149R mutation results in any changes in the levels of mitochondrial Fis1 remained undetermined. The K149R mutation in fact increases the levels of Fis1 in mitochondria, and fusion of SUMO‐2 to Fis1, to mimic constitutive SUMOylation (Fis1‐SUMO‐2^ΔGG^), seems to relocate Fis1 primarily to the cytosolic fraction (Fig 6A), suggesting that deSUMOylation promotes Fis1 mitochondrial localization. Nevertheless, there was no significant difference between levels of Fis1 WT and Fis1 K149R in the cytosolic fraction, which likely contained peroxisomes. This raised the possibility that Fis1 WT was localized to an intracellular area other than the cytosolic and mitochondrial fractions prepared in this study. Considering that perinuclear ER continuous with the nucleus could be a “contaminant” component in the nuclear fraction during the classic hypotonic buffer‐based preparation (Huber *et al*, 2003; Shaiken & Opekun, 2014), we performed immunocytochemistry to examine the ER distribution of exogenously expressed Fis1. The “constitutively SUMOylated” Fis1‐SUMO‐2^ΔGG^ fusion seemed to be more co‐localized with ER than the Fis1 WT, and Fis1 K149R did not seem to co‐localize with ER (Fig 6B), suggesting that Fis1 in its SUMOylated state is more likely to localize to areas of the cell in which ER is located, whereas non‐SUMOylated Fis1 may preferentially localize away from the ER.

**Figure 7:**
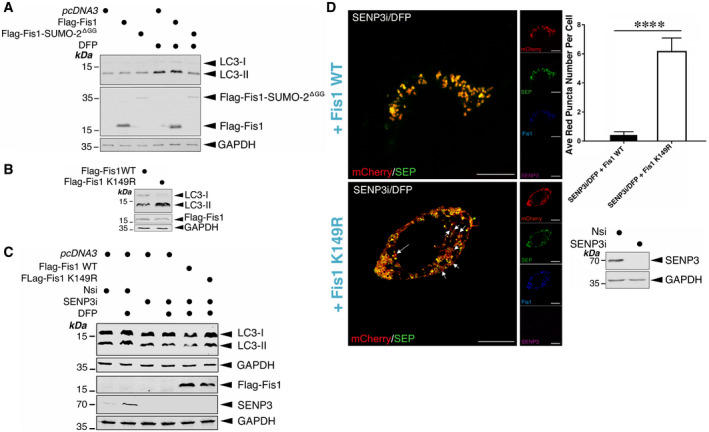
SUMOylatable Fis1 is essential for SENP3 regulation of mitophagy Expression of Flag‐Fis1‐SUMO‐2^ΔGG^, but not Flag‐Fis1, in HeLa cells prevents DFP‐mediated induction of LC3‐II. Flag‐Fis1 WT or Flag‐Fis1‐SUMO‐2^ΔGG^ mutant was transfected into HeLa cells for 48 h and the cells were treated with DFP for a further 24 h. Lysate samples were blotted as indicated.Expression of a SUMOylation‐deficient Flag‐Fis1 K149R mutant in HeLa cells induces LC3‐II. Flag‐Fis1 WT or Flag‐Fis1 K149R mutant was transfected into HeLa cells in which endogenous Fis1 was depleted using siRNA for 48 h. Lysate samples were blotted as indicated.Expressing a SUMOylation‐deficient Fis1 mutant reverses the effect of SENP3 knockdown on DFP‐induced LC3‐II induction. *pcDNA3*, Flag‐Fis1 WT or Flag‐Fis1 K149R mutant was transfected into HeLa cells in which SENP3 was depleted using siRNA for 48 h (Nsi, non‐specific siRNA; SENP3i, SENP3 siRNA (I); siRNA concentration, 20 nM), and the cells were treated with DFP for a further 24 h (DFP, 1 mM). Lysate samples were blotted as indicated.SENP3 depletion does not abolish mitophagic puncta detected in HeLa cells expressing SUMOylation‐deficient CFP‐Fis1 K149R in the presence of DFP. CFP‐Fis1 WT or CFP‐Fis1 K149R mutant were transfected into HeLa cells in which SENP3 was depleted using siRNA for 48 h, and the cells were treated with DFP for a further 24 h (Red: mCherry‐A or puncta indicating occurrences of mitophagy marked by white arrows; Green: SEP‐A; Cyan/blue: CFP‐Fis1; Magenta, SENP3; Scale bar, 10 μm); Values are presented as mean ± SEM (*n* = 21 cells per condition from two individual experiments; ****P < 0.0001; unpaired *t*‐test). Knockdown of SENP3 was further confirmed by immunoblotting (the lower right panel). Source data are available online for this figure.

We then asked if changes in Fis1 SUMOylation status have an impact on mitophagy. We examined the effect of expressing Fis1 WT or Flag‐Fis1‐SUMO‐2^ΔGG^ in HeLa cells on LC3‐II induction by DFP. As expected, expression of the Fis1‐SUMO‐2^ΔGG^ fusion greatly reduced LC3‐II levels in DFP‐treated cells (Fig 7A), suggesting that constitutive SUMOylation of Fis1 may prevent DFP‐induced LC3 lipidation. Moreover, in the absence of DFP, expressing the Fis1 K149R mutant in HeLa cells, where endogenous (human) Fis1 was depleted by siRNA (Appendix Fig S9), led to enhanced LC3‐II levels compared to expressing Fis1 WT (Fig 7B), suggesting promoting Fis1 deSUMOylation may enhance mitophagy *per se*. Taken together, these findings suggest that the SUMOylation status of Fis1 may serve as a key molecular switch to regulate its mitochondrial localization and mitophagic autophagosome formation.

### SENP3 deSUMOylates Fis1 to regulate DFP‐induced mitophagy

The above‐mentioned findings led us to hypothesize that SENP3‐mediated deSUMO‐2/3‐ylation of Fis1 would be a critical step in DFP‐induced mitophagy. To test this hypothesis, we examined the levels of LC3‐II in DFP‐treated HeLa cells in which SENP3 was knocked down and the Flag‐Fis1 WT or the Flag‐Fis1 K149R mutant was expressed. As expected, upon DFP treatment, levels of LC3‐II in SENP3 knocked down cells expressing the SUMOylation‐deficient K149R mutant seemed to be higher than those in the cells expressing the WT counterpart (Fig 7C). Since SENP3 knockdown blocks LC3‐II expression only in the presence of SUMOylatable Fis1, these results indicate that Fis1 is the downstream deSUMOylation target for SENP3‐mediated mitophagic LC3 lipidation. In parallel, we monitored levels of SEP quenching of the mito‐pHfluorin reporter in HeLa cells in which SENP3 was knocked down and CFP‐Fis1 WT or CFP‐Fis1 K149R mutant were expressed. As predicted, upon SENP3 knockdown, there were significantly more autophagic puncta in cells expressing Fis1 K149R than WT Fis1 (Fig 7D), further indicating the importance of the SUMOylation status of Fis1 for SENP3‐mediated autolysosome formation. Taken together, these results support the conclusion that SENP3‐mediated deSUMO‐2/3‐ylation of Fis1 is required for this previously uncharacterized mitophagy pathway.

## Discussion

**Figure 8:**
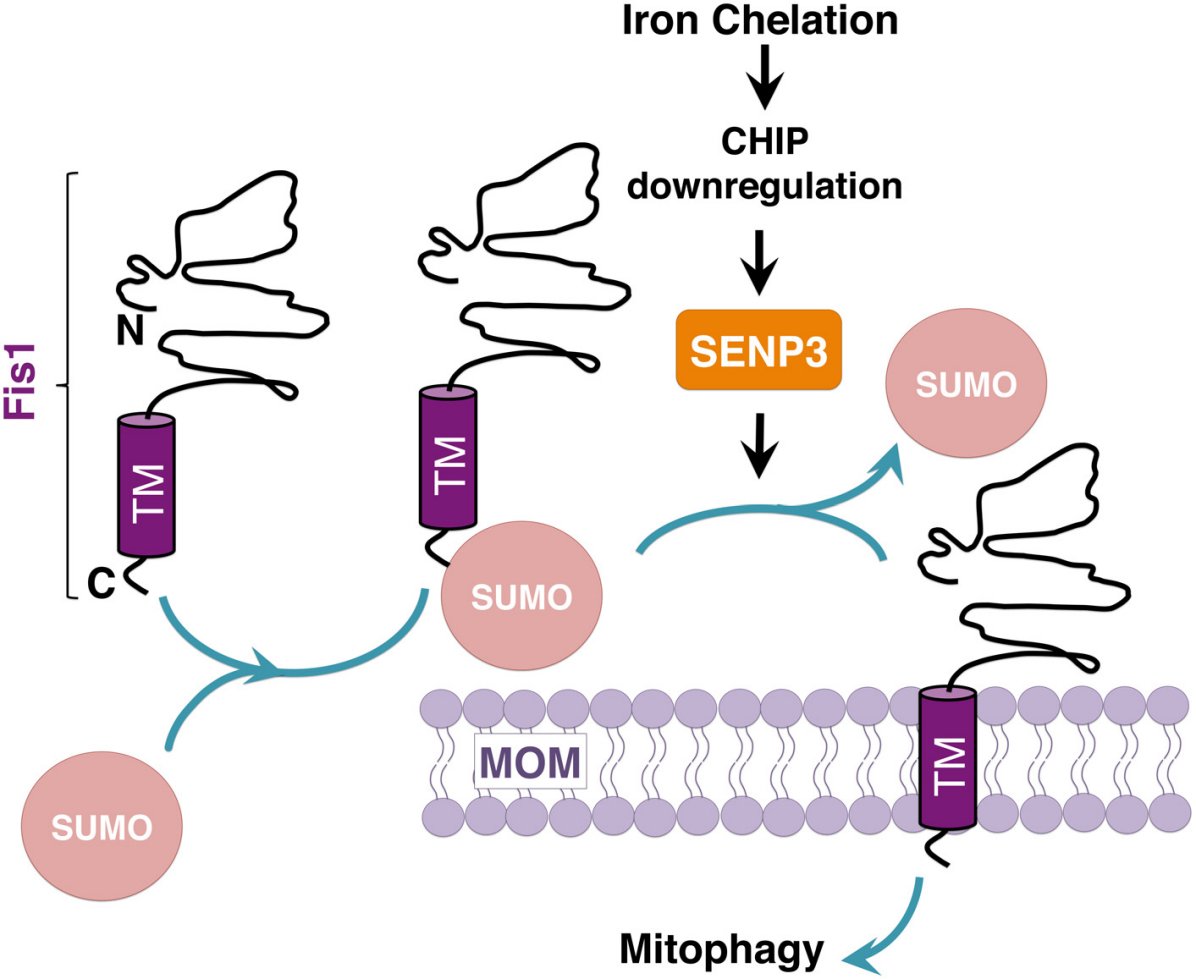
Schematic showing the roles of SUMOylation and SENP3‐mediated deSUMOylation in Fis1 mitochondrial localization DFP treatment enhances SENP3‐mediated deSUMOylation of Fis1, thereby facilitating Fis1 mitochondrial localization that is required for mitophagy.

Healthy mitochondria are vital to generate energy for eukaryotic cells and accumulation of dysfunctional mitochondria has been linked to ageing and age‐related diseases. This age‐related pathological feature is believed to be due to defective mitophagy, which mediates mitochondrial quality control via lysosomal degradation. However, the mechanisms that regulate mitophagy and the key pathways involved in mitochondrial quality control remain largely unknown. Here we report a previously uncharacterized pathway that regulates mitophagy via modulation of the SUMOylation status of the mitochondrial protein Fis1 by the SUMO‐2/3‐specific protease SENP3 (Fig 8). To the best of our knowledge, this is the first example showing functional crosstalk between protein SUMOylation and regulation of mitophagy.

### Modulation of SENP3 stability by mitophagy induction

In mammals the Ub‐proteasome system (UPS) controls SENP3 levels through proteasome‐mediated degradation (Kuo *et al*, 2008; Yan *et al*, 2010). It has been shown previously that the E3 Ub ligase CHIP mediates UPS‐dependent degradation of SENP3 (Yan *et al*, 2010). In this study, we found that upon DFP‐mediated iron chelation, SENP3 is stabilized while levels of CHIP and *CHIP* mRNA are reduced, suggesting that *CHIP* gene downregulation leads to decreased CHIP levels, resulting in SENP3 stabilization through a reduction in SENP3 degradation. In future studies it will be necessary to establish how DFP‐mediated iron chelation reduces *CHIP* gene expression.

A key finding of this study is that SENP3 is pivotal for mitophagy induction by iron chelation. SENP3 stabilization is temporally correlated with decreased levels of global SUMO‐2/3 conjugation. Consistent with this, knockdown of SENP3 reduces DFP‐induced formation of both mitophagic autophagosomes and autolysosomes, further suggesting that sufficient levels of SENP3 are required for the occurrence of mitophagy upon iron chelation. We further identify Fis1 as a key target for SENP3‐mediated deSUMOylation in mitophagy regulation since expression of a SUMOylation‐deficient mutant of Fis1 abolishes the effect of SENP3 knockdown on DFP‐induced mitophagy.

### Role of SENP3 in autophagy regulation

Deferoxamine is the only iron chelator known to cause ferritin loss through ferritinophagy‐mediated lysosomal degradation, and the mechanisms underlying the effect of the other two iron chelators DFP and deferasirox on ferritin degradation are currently unclear (De Domenico *et al*, 2009). Consistent with this, we found that treatment of HeLa cells with DFP (1mM; 24h) results in a significant loss of ferritin heavy chain 1 (FTH1), and SENP3 knockdown did not have an effect on DFP‐induced loss of FTH1 (Appendix Fig S10), suggesting a negative role for SENP3 in ferritin degradation caused by DFP.

Interestingly, we found that increased levels of SENP3 are also inducible using the mitochondrial stressors CCCP and paraquat (Appendix Figs S11 and S12). However, SENP3 knockdown did not seem to have an effect on LC3‐II induction by CCCP in HEK293 cells, implying that SENP3 may not be important for mitophagy dependent on Parkin. Nevertheless, since current evidence shows Fis1 involvement in Parkin‐dependent mitophagy (Yamano *et al*, 2014; Rojansky *et al*, 2016), in future it will be necessary to fully assess the role of SENP3‐mediated Fis1 deSUMOlyation in regulating Parkin‐dependent mitophagic autolysosome formation using the reporters used in this study. Moreover, consistent with a most recent report where SENP3 has an inhibitory role in macroautophagy through deSUMOlyating beclin 1 (Liu *et al*, 2020), SENP3 knockdown seemed to increase basal LC3‐II levels and prevented any further increase in LC3‐II in paraquat‐treated SH‐SY5Y cells, implying that SENP3 may have a role in macroautophagy and/or mitophagy induced by paraquat.

### SUMOylation and deSUMOylation of Fis1 and Fis1 mitochondrial localization

The canonical mitophagy pathway involves Pink1 and Parkin. However, mice lacking either Pink1 or Parkin show very mild defects in mitochondrial function (Whitworth & Pallanck, 2017). In contrast, genetic depletion of Fis1 in mammalian cells blocks mitophagy‐mediated disposal of dysfunctional mitochondria (Shen *et al*, 2014; Yamano *et al*, 2014; Rojansky *et al*, 2016), signifying a major role for Fis1 in mitochondrial quality control. However, it remains unclear how Fis1 mediates mitophagy. Emerging evidence indicates that mitophagy induction requires Fis1 mitochondrial localization (Rojansky *et al*, 2016). In keeping with the importance of Fis1 mitochondrial localization, our results showing that mutation of the major SUMOylated lysine (K149) enhances its mitochondrial presence indicate that preventing SUMOylation promotes Fis1 mitochondrial localization and suggest that SUMO‐2/3 modification may act as a molecular switch to regulate levels of mitochondrial Fis1. However, it should be noted that, in fact, at any given time only a small proportion of Fis1 is SUMOylated. This argues against constitutive SUMOylation being required to maintain Fis1 away from mitochondria. Rather, similar to what we have previously suggested regarding the roles for SUMOylation in Drp1 mitochondrial partitioning (Guo *et al*, 2013), deSUMOylation may act as a “mobilization factor” for Fis1 mitochondrial targeting. Moreover, the C‐terminus of Fis1 is known to be essential for its interaction with TBC1D15 (Onoue *et al*, 2013), a Rab GTPase‐activating protein (Rab‐GAP) required for mitophagic autophagosome formation (Yamano *et al*, 2014). Therefore, in future it will be necessary to establish if Fis1 SUMOylation status regulates Fis1 binding to TBC1D15 to regulate mitophagy. Furthermore, the fact that DFP treatment leads to a significant loss of Fis1 in the model cells has made it technically difficult to compare Fis1 SUMOylation levels between DFP‐treated cells and their controls. Therefore, in this respect, it will be necessary to develop new experimental tools/assays for demonstrating SENP3‐mediated deSUMOylation of Fis1 upon DFP‐mediated iron chelation in future.

In conclusion, we define a previously unidentified SUMO‐dependent pathway in which UPS‐dependent stabilization of SENP3 upon iron chelation enhances mitochondrial targeting of Fis1 to facilitate mitophagy induction. This pathway represents a novel example of a target‐specific function of SENP3 in a mitochondrial quality control programme and reveals a potential therapeutic target for restoring impaired or defective mitophagy in health and disease.

## Materials and Methods

### Cloning and mutagenesis

DNA constructs encoding Flag‐SENP3, His‐SUMO‐2 and GST‐(mouse) Fis1 were described previously (Guo *et al*, 2013, 2017). Flag‐Fis1 was generated by insertion of the relevant cDNA amplified from GST‐Fis1 into the BamH1/Not1 sites of pcDNA3. Flag‐Fis1 mutants, K67R, K119R, K149R, K151R and Δ149‐152, were made by PCR‐based mutagenesis. To potentially mimic constitutive SUMOylation of Fis1, Flag‐Fis1 was fused with SUMO‐2^ΔGG^ in tandem by insertion mutagenesis. CFP‐Fis1 was generated by sequential insertions of cDNA‐encoding CFP and cDNA‐encoding mouse Fis1 into the BamHI/EcoRI sites and EcoRI/NotI sites of pcDNA3 respectively. To visualize “mitophagic flux,” a novel probe (mito‐pHfluorin) was generated as follows: cDNA sequences encoding Super‐ecliptic pHluorin (SEP, a pH‐sensitive GFP variant) and the mitochondrial target sequence (LILAMLAIGVFSLGAFIKIIQLRKNN, termed as “A”) of the ActA protein from *Listeria monocytogenes* were amplified using PCR from SEP‐TOPO (Ashby *et al*, 2004) and GFP‐A (Guo *et al*, 2017) respectively. The plasmid encoding the fusion protein mCherry‐SEP‐A was then made by sequential insertion of the cDNA‐encoding SEP and the cDNA‐encoding A into a pmCherry‐C3 construct. To illustrate ER outline, a mammalian expression plasmid was generated by insertion of the cDNA sequence encoding the cytochrome b5 (Cb5) ER membrane‐targeting sequence (ITTVESNSSWWTNWVIPAISALVVALMYRLYMAED), which was PCR amplified from pEF6‐Bcl‐xL‐Cb5 (Eno *et al*, 2012), into EcoRI/BamHI sites of pEGFP‐C1, termed as GFP‐Cb5.

### Cell culture

HeLa, HEK293 and SH‐SY5Y cells were cultured in Dulbecco’s modified Eagle’s medium (DMEM; Lonza) containing 10% foetal bovine serum (FBS), 5 mM glutamine and 100 units/ml penicillin/streptomycin at 37°C in humidified air supplemented with 5% CO_2_, as previously described (Guo *et al*, 2013, 2017). HEK293 N3S cells stably expressing 6His‐SUMO‐2^T90K^ (Tammsalu *et al*, 2014) were a gift from R.T. Hay and were cultured in DMEM containing 10% FBS, 5 mM glutamine, 100 units/ml penicillin/streptomycin in the presence of 1 μg/ml puromycin.

### Mitophagy induction

Confluent HeLa cells grown in 6‐ or 12‐well plates were treated with deferiprone (DFP; 379409, Sigma‐Aldrich; working concentration, 1 mM) for the time durations indicated. For better LC3‐II visualization, CQ (50 μM) was added to the cells to prevent lysosomal degradation for the final 12 h of DFP treatment in the experiments shown in Figs 3A and B, 4A–C, and 7A–C and in some experiments as indicated in Appendix Figs S1, S2 and S4.

Confluent HEK293 cells grown in 12‐well plates were treated with Carbonyl cyanide 3‐chlorophenylhydrazone (CCCP; C2759, Sigma‐Aldrich; working concentration 10–100 μM) for 2 h.

Confluent SH‐SY5Y cells grown in 12‐well plates were treated with Paraquat (36541, Sigma‐Aldrich; working concentration 10 μM) for 4 h.

### DNA and siRNA transfections

For DNA, siRNA or DNA‐siRNA co‐transfection into HeLa or HEK293 cells, we used jetPRIME reagents (Polyplus Transfection) as previously described (Guo *et al*, 2013, 2017), according to the manufacturer’s instructions, and used the cells within 72 h. siRNA duplexes used were as follows: non‐specific siRNA (Eurofins Genomics), human SENP3 siRNA (I) (sc‐44451, Santa Cruz), human SENP3 siRNA (II) (duplexes to target AACGUGGACAUCUUCAAUA to silence SENP3; synthesized by Eurofins Genomics), Fis1 siRNA (a pool of two duplexes to target two sequences, CUACCGGCUCAAGGAAUAC and GGAAUACGAGAAGGCCUUA, to silence Fis1; synthesized by Eurofins Genomics) and Drp1 siRNA used previously (Guo *et al*, 2013).

To replace endogenous SENP3 with RNAi‐resistant GFP‐SENP3 wild‐type (GFP‐SENP3^R^ WT) or GFP‐SENP3 C532A mutant (GFP‐SENP3^R^ C532A), the underlined nucleotide C was changed to T to introduce a silent mutation in the SENP3 siRNA target sequence (1339‐AACGTGGACATCTTCAATA) in pEGFP‐C1‐SENP3 or pEGFP‐C1‐SENP3 C532A (Guo *et al*, 2013). The two sequences chosen to silence (human) Fis1 as mentioned above are not present in mouse Fis1, therefore, plasmids encoding mouse Fis1 are naturally resistant to the siRNA targeted against human Fis1. YFP‐Drp1^R^ WT or YFP‐Drp1^R^ 4KR was used to replace endogenous Drp1 as described previously (Guo *et al*, 2013).

### Establishment of HeLa cell line with stable expression of mito‐Keima

A mammalian expression plasmid encoding mito‐Keima (Addgene #56018) was transfected into HeLa cells. Two days post‐transfection the cells were sub‐cultured by repeated passaging in the presence of G418 (400 μg/ml; Roche) for 4 weeks, and flow cytometry‐automated cell sorting was performed to isolate cells expressing high levels of green fluorescence. Following sorting HeLa cells stably expressing mito‐Keima were cultured continuously in the presence of G418 (200 μg/ml).

### Creation of Fis1^−/−^ HeLa cells

To target human Fis1, oligos encoding a guide RNA (gRNA) against Fis1 (gRNA sequence: AGACACCAGCTCGTTCAGCA) was designed using the ATUM tool (https://www.atum.bio/eCommerce/cas9/input) and cloned into the plasmid pSpCas9(BB)‐2A‐Puro (Addgene #62988), which expresses both a gRNA and Cas9, and confers puromycin resistance on transfected cells. HeLa cells were transfected with the gRNA for 48 h, before being treated with puromycin (2 μg/ml; Sigma) for a further 48 h. Cells were then changed into media lacking puromycin and grown until 80% confluent. Clones originating from individual cells were then generated by limiting dilution into 96‐well plates, expanded and tested for Fis1 loss by Western blotting. One Fis1 lacking clone, denoted 2‐1‐3, was used for the experiments shown here.

### RNA isolation and quantitative real‐time PCR (qPCR)

The RNA extracted from cultured HeLa cells treated with DFP and control samples was purified using RNeasy (Qiagen), according to the manufacturer’s instructions. Total RNA was calculated using a nanodrop 1000 spectrophotometer and 100 ng of total RNA was reverse transcribed using a high‐capacity cDNA reverse transcription kit (Thermo Fisher Scientific), according to the manufacturer’s instructions. Quantitative polymerase chain reaction (qPCR) was performed using TaqMan™ gene expression assays as follows: 0.5 µl cDNA was amplified using 5 µl master‐mix, 3.5 µl nuclease‐free water and 0.5 µl TaqMan™ gene probe (FAM), and 0.5 µl β‐2‐Microglobulin (VIC) was used as a reference control (Thermo Fisher Scientific). Reactions were performed using the following schedule of 50°C (2 min) and 95°C (10 min) then 40 cycles of 15 s at 95°C followed by 1 min at 60°C using a Rotor‐Gene Q 2 Plex Real‐time PCR Cycler (Qiagen). The threshold cycle (Ct) was normalized against the reference gene and then fold‐changes in expression relative to the untreated group were calculated using the formula 2^−ΔΔCt^ (Livak & Schmittgen, 2001). The probe sequences used were for human *CHIP*, *SENP3* and *FIS1;* 5′‐TCTGCAGCGAGCTTACAGCCTGGCC‐3′, 5′‐ACAGTCCCTGAAAAGGTGCATTTCT‐3′ and 5′‐GGAACTACCGGCTCAAGGAATACGA‐3′ respectively.

### Subcellular fractionation

Cytosol and mitochondria fractions were prepared from HeLa cells using a Cell Fractionation Kit (Abcam), according to the manufacturer’s instructions.

### Preparation of cell lysate samples and immunoprecipitation

Treated/transfected HeLa or HEK293 cells were washed once with ice‐cold phosphate‐buffered saline and then lysed in a buffer containing 20 mM Tris, pH 7.4, 137 mM NaCl, 25 mM β‐glycerophosphate, 2 mM sodium pyrophosphate, 2 mM EDTA, 1% Triton X‐100, 10% glycerol and 1× cOmplete™ Protease Inhibitor Cocktail (Roche). N‐Ethylmaleimide (NEM; 20 mM) was added when protein samples were prepared to assess effects of SUMOylation/deSUMOylation. Following sonication, insoluble material was removed from lysates by centrifugation for 15 min at 16,000 *g* at 4°C. In experiments to detect endogenous Fis1 SUMO‐2/3‐lyation, cells were lysed in the above‐mentioned buffer plus 1% SDS, and heated at 95°C for 5 min. The lysate samples were then diluted 1:10 in the same buffer without SDS in the presence of 20 mM NEM. Using SUMO‐2/3 affinity beads (ASM24, Cytoskeleton), endogenous SUMO‐2/3 conjugates from the lysate samples were immunoprecipitated.

### Histidine pulldown

His‐SUMO‐2 conjugates were purified through histidine pulldowns (His‐PD) under denaturing conditions as previously described (Guo *et al*, 2013, 2017). Briefly, transfected HEK293 cells or HEK293 N3S cells stably expressing 6His‐SUMO‐2^T90K^ were immediately lysed in a denaturing buffer containing 6 M guanidinium‐HCl, 0.1 M Na_2_HPO_4_/NaH_2_PO_4_, 0.01 M Tris‐HCl, pH 8.0, sonicated and incubated with Ni^2+^–NTA beads (Qiagen) for 3 h at room temperature (RT). Following the incubation, the nickel beads were washed twice with the denaturing buffer, two times with a wash buffer containing 8 M urea, 0.1 M Na_2_PO_4_/NaH_2_PO_4_, 0.01 M Tris‐HCl, pH 6.3 and twice with PBS. Bound proteins were eluted in 6× SDS loading buffer and resolved by SDS–PAGE. His‐SUMO‐2 conjugates were immunoblotted using an antibody against Flag or Fis1.

### Immunoblotting

Lysate, IP or His‐PD Samples were resolved by SDS–PAGE (10–15% gels) and transferred to Immobilon‐P membranes (Millipore Inc.), which were then immunoblotted with the following antibodies against: β‐actin (Sigma), CHIP (Cell Signaling), Drp1 (Cell Signaling), Fis1 (Proteintech), Flag (Proteintech), FTH1 (Cell Signaling), GAPDH (Santa Cruz biotechnology), GST (GE Healthcare), LC3 (Cell Signaling), p62 (Cell Signaling), SENP3 (Cell Signaling), SENP5 (Proteintech), SUMO‐1 (Santa Cruz biotechnology), SUMO‐2/3 (Cell Signaling; MBL) or Tom20 (Santa Cruz biotechnology). Immune complexes were detected using either HRP‐conjugated secondary antibodies (Sigma) followed by enhanced chemiluminescence (GE Healthcare) or using fluorescent secondary antibodies (LI‐COR).

Each immunoblot presented is representative of at least three experiments carried out using different cell populations.

### Fluorescence and microscopy imaging

HeLa cells were plated on 35‐mm glass bottom dishes and transfected after 24 h. After 48 h, cells were treated with DFP (1 mM) or PBS for a further 24 h. Cells were fixed for 12 min at RT in 4% paraformaldehyde/PBS. Following three 5‐min washes with PBS to remove residual PFA, cells were permeabilized in 0.3% Triton X‐100/PBS for 15 min and then blocked for 1 h in 0.01% Triton X‐100, 0.3% Fish skin gelatin/PBS. Antibodies were diluted in blocking buffer and incubated for 1.5 h at RT. Immunostaining was performed using the following antibodies: COX IV (3E11, 1:250; Cell signaling;), Fis1 (10956‐1‐AP, 1:400; Proteintech), Flag (66008‐3‐Ig, 1:500; Proteintech), Mff (17090‐1‐AP; 1:200; Proteintech) and SENP3 (D20A10, 1:400; Cell signaling). Cells were analysed with a confocal microscope (Zeiss LSM 880 Airyscan) within the Wolfson Light Microscope Facility at the University of Sheffield. Adjustments of Brightness and contrast using Fjji ImageJ (https://imagej.net/software/fiji/) were applied to each individual confocal image as a whole, as little as possible and under conditions the adjustments do not alter any information contained in the original image.

To detect mitophagy in living HeLa cells stably expressing mito‐Keima under different conditions, unfixed cells grown in six‐well plates were analysed with an EVOS‐fl inverted LED fluorescence microscope (AMG). For each condition, cells in three random microscopic fields were counted manually.

To detect the co‐localization of red‐alone puncta visualized by mito‐pHfluorin and lysosomes, LysoTracker (Blue DND‐22; Invitrogen) was used for staining HeLa cells expressing mito‐pHfluorin.

### Establishment of criteria to quantify autolysosome formation using mito‐pHfluorin

**Figure EV5:**
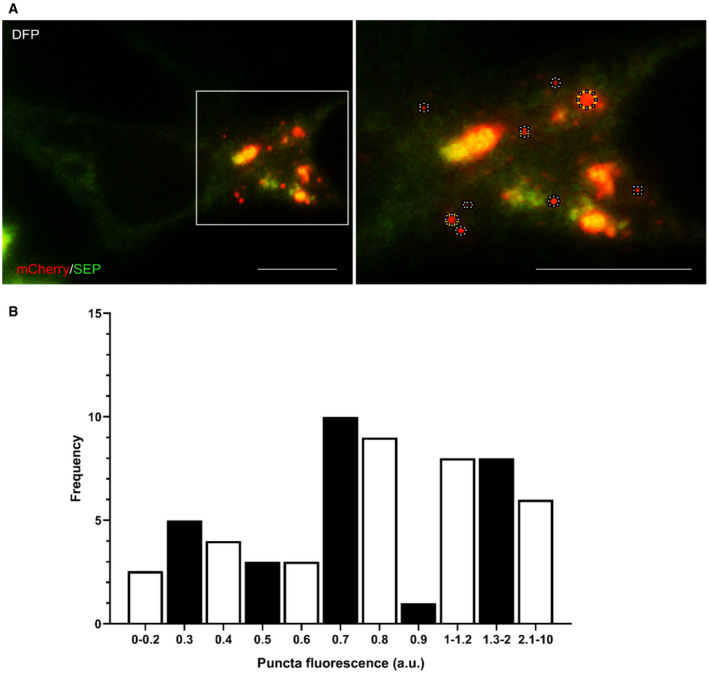
Criteria used for quantifying mitophagy using Mito‐pHfluorin Mito‐pHfluorin was transfected into HeLa cells for 48 h. DFP (1 mM; 24 h) was then added to induce mitophagy. The left panel shows a representative image, displaying the heterogeneity of the autolysosome population in a DFP‐treated cell. Using ImageJ software, autolysosome intensity was measured by drawing around the red puncta (the right panel showing the boxed area of interest in the left panel; dotted circles; Scale bar, 10 μm).A frequency histogram showing that the intensities of the autolysosomes range considerably, from ≤ 0.2 A.U to 10 A.U: quantification of the frequency of red puncta intensity (*n* = 44) with a median determined to be 0.7 A.U. This was taken as the threshold for an autolysosome. The median was chosen to better reflect the central tendency and reduce the effect that outliers may have. In addition, the mode, as depicted in the frequency histogram as the highest peak, was also 0.7 A.U, suggesting that 0.7 A.U is frequent intensity for the red puncta and is likely to represent autolysosomes. Any autolysosomes detected that were double this size (1.4 A.U) were counted as two autolysosomes etc. Together, this confirms the heterogeneity of autolysosomes and establishes a criterion for autolysosome detection by mito‐pHfluorin.

To successfully conduct quantitative analysis using mito‐pHfluorin, validation of the tool was first performed to define the criteria for autolysosome detection. Autolysosomes are heterogeneous in size ranging from several hundred nanometres to micrometre range, and it is known that multiple autophagosomes can fuse with the same lysosome (Rusten *et al*, 2004). To observe the heterogeneity of autolysosome formation, HeLa cells were transfected with mito‐pHfluorin and treated with 1 mM DFP for 24 h to induce mitophagy. Within each cell, there was a heterogeneous population of autolysosomes that ranged in diameter. One representative image, displaying the heterogeneity of the autolysosome population, is shown in Fig EV5. ImageJ analysis was used to measure the area of the autolysosomes detected upon DFP treatment to produce a reading of fluorescence intensity, as shown by the dotted circles surrounding the red puncta (Fig EV5A). It can be seen from the frequency histogram that the intensities of the autolysosomes range considerably, from ≤ 0.2 fluorescence Arbitrary Unit (A.U) to 10 A.U (Fig EV5B). From this, the median was calculated to be 0.7 A.U and thus, this was taken as the threshold for an autolysosome. The median was chosen to better reflect the central tendency and reduce the effect that outliers may have. In addition, the mode, as depicted in the frequency histogram as the highest peak, was also 0.7 A.U, suggesting that 0.7 A.U is a frequent intensity for the red puncta and likely represents autolysosomes (Fig EV5). Any autolysosomes detected that were double this size (1.4 A.U) were counted as two autolysosomes etc. These criteria were then used for all mito‐pHfluorin‐based analyses presented here.

### Statistics

For comparison of conjugates of SUMO‐2/3 or SUMO‐1, and levels of SENP3, SENP5, CHIP or LC3‐II in lysate or His‐PD samples between time‐matched two groups, paired Student's *t*‐test with a two‐tail *P*‐value was performed. For analysis of mitophagic autolysosomes, unpaired Student’s *t*‐tests with two‐tail *P*‐value was performed comparing mean of red punctum numbers. Following quantification using ImageJ software, or LI‐COR Image Studio for blots developed with fluorescence, levels of proteins of interest were normalized to β‐actin or GAPDH control levels. Each value is presented as mean ± SEM and is expressed as percentage of control value. To avoid bias all experiments were performed blind.

## Author contributions

CG and EW conceived the project with valuable input from KAW. EW, KAW and CG designed and performed most biochemical and molecular biological experiments, EW and DR performed all cell imaging assays in cell culture, KAW generated Fis1 knockout cell lines, REC conducted some cell culture and sample preparation work, and ALH and HEC conducted RNA extraction and qPCR analysis. CG provided project management and wrote the manuscript with hypothesis development, experimental design and data interpretation contributed by all authors. The funders had no role in study design, data collection and analysis, decision to publish or preparation of the manuscript.

## Supporting information



Appendix

Expanded View Figures PDF

Source Data for Figure 1

Source Data for Figure 2

Source Data for Figure 3

Source Data for Figure 4

Source Data for Figure 5

Source Data for Figure 6

Source Data for Figure 7

## Data Availability

This study includes no data deposited in public repositories.
