# Supplementary material for: The SUMO protease SENP3 regulates mitochondrial autophagy mediated by Fis1
Source: EMBO Rep. 2022 Jan 7;23(2):e48754. doi: 10.15252/embr.201948754 (PMC8811651; doi:10.15252/embr.201948754)
Supplement: Supplementary file 1 — Appendix [file EMBR-23-e48754-s004.pdf]

The SUMO Protease SENP3 regulates Mitochondrial Autophagy mediated by Fis1

Emily Waters[1§], Kevin A. Wilkinson[2§], Amy L. Harding[3], Ruth E. Carmichael[2], Darren Robinson[1], Helen E. Colley[3], and Chun Guo[1*]

[1]School of Biosciences, University of Sheffield, Firth Court, Western Bank, Sheffield, S10 2TN, U.K.

[2]School of Biochemistry, Medical Sciences Building, University of Bristol, University Walk, Bristol, BS8 1TD, U.K.

[3]School of Clinical Dentistry, University of Sheffield, Sheffield, S10 2TA, U.K.

[§] These authors contributed equally to this work.

*Correspondence to:

Chun Guo, School of Biosciences, University of Sheffield, Firth Court, Western Bank, Sheffield, S10 2TN, U.K. Tel.: +44 114 222 3648; E-mail: c.guo@sheffield.ac.uk

**Content**	**Page number**
Appendix Figure S1	2
Appendix Figure S2	3
Appendix Figure S3	4
Appendix Figure S4	5
Appendix Figure S5	6
Appendix Figure S6	7
Appendix Figure S7	8
Appendix Figure S8	9
Appendix Figure S9	10
Appendix Figure S10	11
Appendix Figure S11	12
Appendix Figure S12	13
Appendix Table S1	14

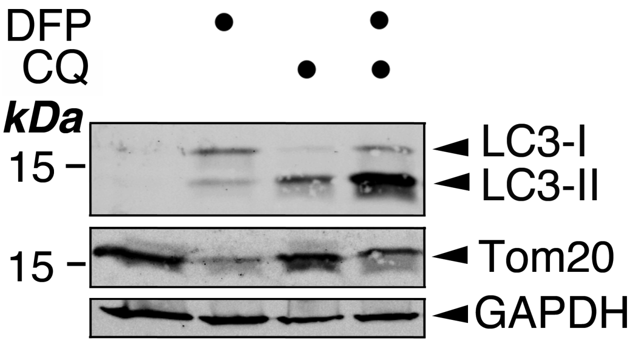

Appendix Figure S1. Chloroquine inhibits DFP-induced lysosomal degradation. DFP (1mM for 24 h) induces LC3-II in HeLa cells. To increase visualization of the LC3 lipidation marker LC3-II, as a widely used autophagy inhibitor, chloroquine (CQ; 50μM for final 12 h of DFP treatment) was added to the cells to prevent lysosomal degradation after 12 h of DFP treatment. Whole cell lysate samples were blotted as indicated.

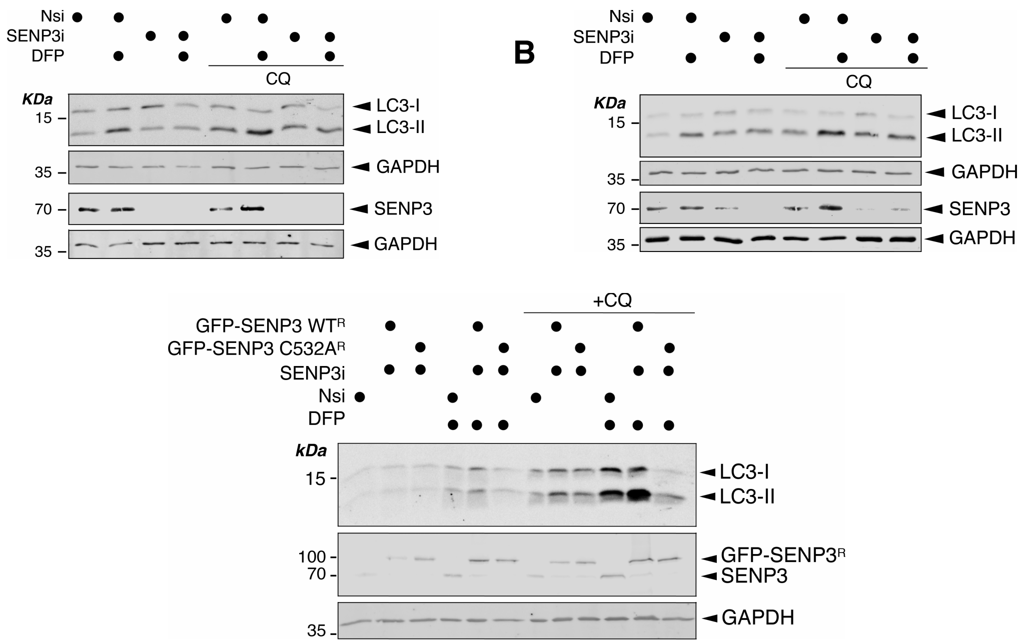

Appendix Figure S2. The deSUMOylating activity of SENP3 is required for DFP-mediated LC3-II induction.

A. Effects of siRNA duplex I-mediated SENP3 knockdown on LC3-II induction by DFP. Nsi or SENP3i (I) was transfected into HeLa cells. Two days post-transfection the cells were treated with DFP (1mM) for a further 24 h in the absence or presence of CQ (50µM for final 12 h of DFP treatment). Whole cell lysate samples were blotted as indicated.

B. Effects of siRNA duplex II-mediated SENP3 knockdown on LC3-II induction by DFP. Nsi or SENP3i (II) was transfected into HeLa cells. Two days post-transfection the cells were treated with DFP (1mM) for a further 24 h in the absence or presence of CQ (50µM for final 12 h of DFP treatment). Whole cell lysate samples were blotted as indicated.

C. Effects of replacement of endogenous SENP3 with GFP-SENP3^R WT or GFP-SENP3^R C532A on LC3-II induction by DFP. GFP-SENP3^R WT or SENP3^R C532A was expressed in HeLa cells after knockdown of endogenous SENP3 using siRNA (II). The cells were treated with DFP (1mM for 24 h) in the absence or presence of CQ (50µM; for final 12 h of DFP treatment). Whole cell lysate samples were blotted as indicated. This panel is the full blot of data shown in **Figure 3B**.

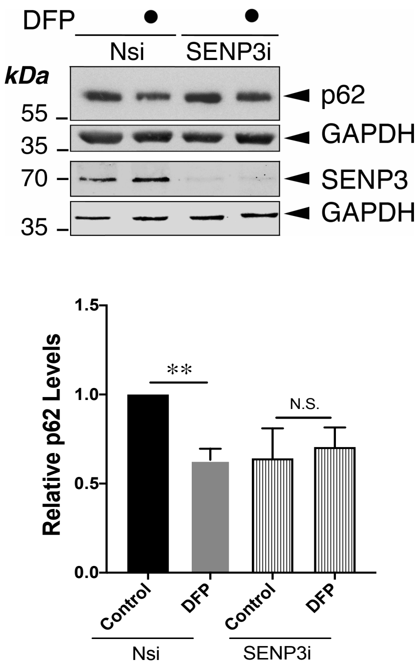

Appendix Figure S3. Knockdown of SENP3 prevents the DFP-induced reduction in p62.

Nsi or SENP3i (I) was transfected into HeLa cells. Two days post-transfection the cells were treated with DFP (1mM) for a further 24 h. Whole cell lysate samples were blotted as indicated. (n=6, biological replicates; **p<0.01; N.S., not statistically significant (P>0.05); Repeated measures analysis of variance followed by Tukey's multiple comparisons test).

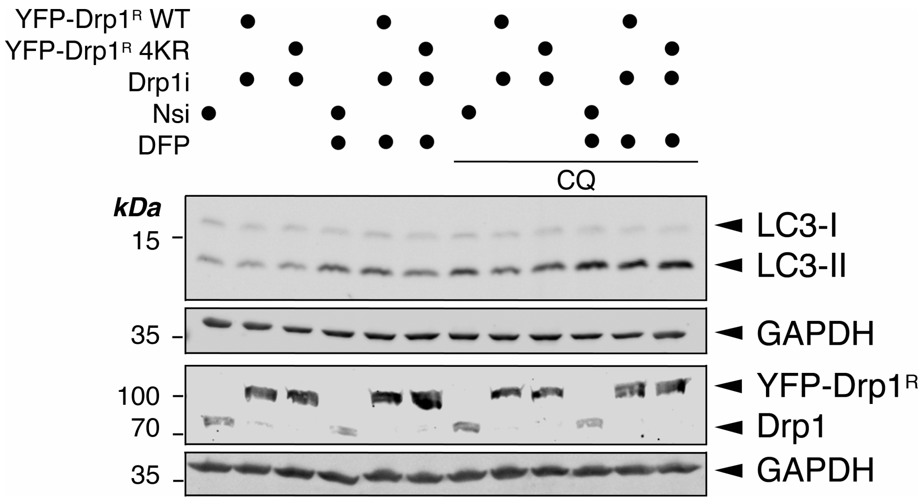

Appendix Figure S4. Drp1 SUMOylation status does not regulate LC3-II induction by DFP

YFP-Drp1^R WT or non-SUMOylatable YFP-Drp1^R 4KR was expressed in HeLa cells after siRNA-mediated knockdown of endogenous Drp1 (Drp1i, Drp1 siRNA; Nsi, non-specific siRNA). The cells were treated with DFP (1mM for 24 h) in the absence or presence of CQ (50µM; for final 12 h of DFP treatment). Whole cell lysate samples were blotted as indicated.

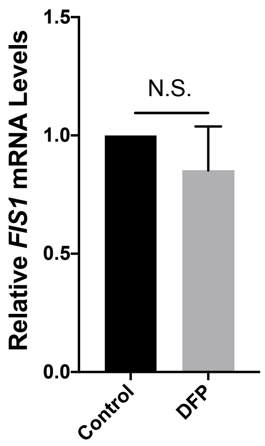

Appendix Figure S5. Treatment of HeLa cells with DFP does not affect *FIS1* mRNA levels

Treatment of HeLa cells with DFP (1mM for 24 h) does not result in changes in levels of *FIS1* mRNA, as determined by qPCR, and *FIS1* mRNA level values are presented as mean ± SEM and are normalised to the control value. (n=3, biological replicates; N.S., not statistically significant ($p>0.05$); unpaired t-test).

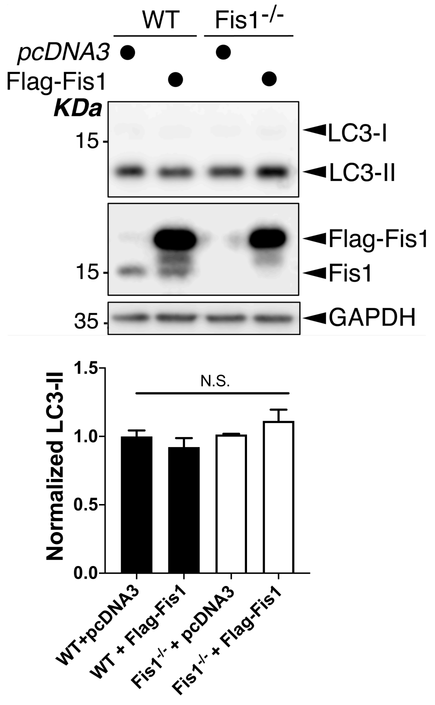

Appendix Figure S6. Fis1 overexpression does not cause changes in LC3-II levels in HeLa cells

WT or Fis1 KO HeLa cells were transfected with pcDNA3 or Flag-Fis1 for 48 h. Whole cell lysate samples were blotted as indicated (n=3, biological replicates; $p>0.05$; One-way ANOVA).

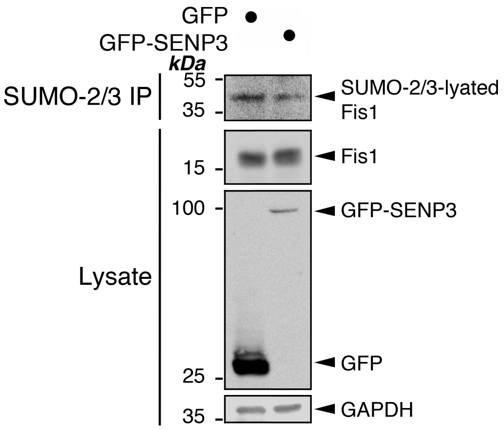

Appendix Figure S7. SENP3 overexpression decreases endogenous Fis1 SUMO-2/3-ylation in HeLa cells.

SUMO-2/3 conjugates were immunoprecipitated (IP) from whole cell lysates prepared from HeLa cells expressing GFP or GFP-SENP3 using SUMO-2/3 affinity beads. IP and lysate samples were blotted as indicated.

A

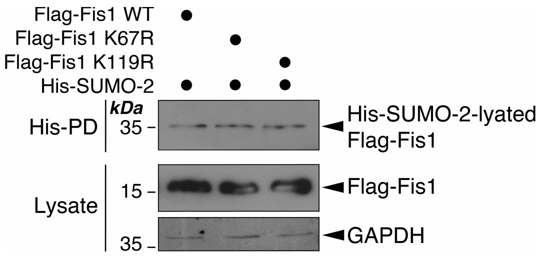

B

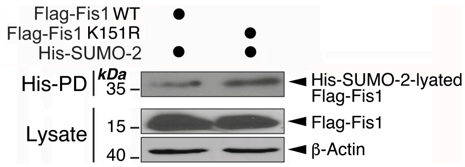

Appendix Figure S8. Mutation of lysine 67, lysine 119 or lysine 151 to arginine does not reduce Fis1 SUMOylation.

A. Flag-Fis1 WT, Flag-Fis1 K67R or Flag-Fis1 K119R mutants, together with His-SUMO-2, were transfected into HEK293 cells expressing Ubc9 for 48 h.
B. Flag-Fis1 WT, or the Flag-Fis1 K151R mutant, together with His-SUMO-2, were transfected into HEK293 cells expressing Ubc9 for 48 h.

In (A) and (B), His-pulldown and lysate samples were detected by immunoblotting for Flag or β-actin.

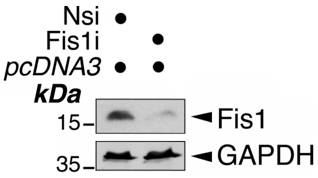

Appendix Figure S9. Knockdown of Fis1 in HeLa cells

Plasmid *pcDNA3* (the control plasmid for Flag-Fis1 or Flag-Fis1 K149R) was transfected into Fis1 knockdown HeLa cells for 48 h. Knockdown of Fis1 was confirmed by immunoblotting (Nsi, non-specific siRNA; Fis1i, Fis1 siRNA).

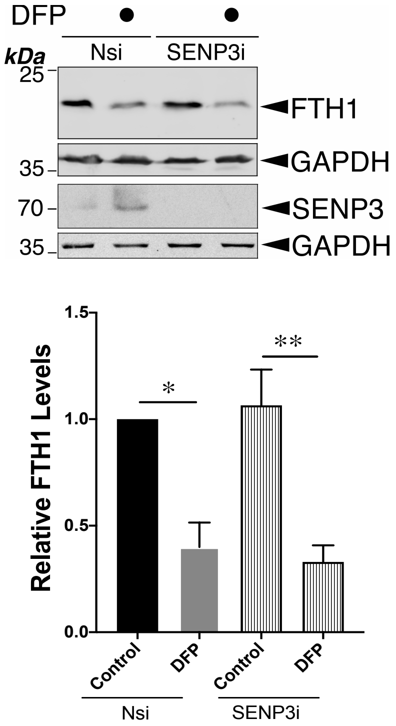

Appendix Figure S10. Absence of SENP3 does not affect FTH1 reduction by DFP.

Nsi or SENP3i (I) was transfected into HeLa cells. Two days post-transfection the cells were treated with DFP (1mM) for a further 24 h. Whole cell lysate samples were blotted as indicated, and FTH1 level values are presented as mean ± SEM and are normalised to the control value. (n=5, biological replicates; *, $p<0.05$; **, $p<0.01$; Repeated measures analysis of variance followed by Tukey's multiple comparisons test).

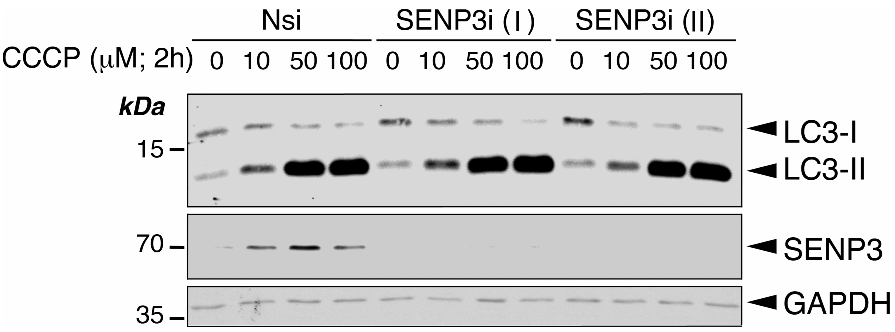

Appendix Figure S11. Absence of SENP3 does not seem to affect LC3-II induction by CCCP

Nsi, SENP3i (I) or SENP3i (II) (synthesized by Eurofins MWG Operon) was transfected into HEK293 cells. Two days post-transfection the cells were treated with CCCP at indicated concentrations for 2 h. Whole cell lysate samples were blotted as indicated.

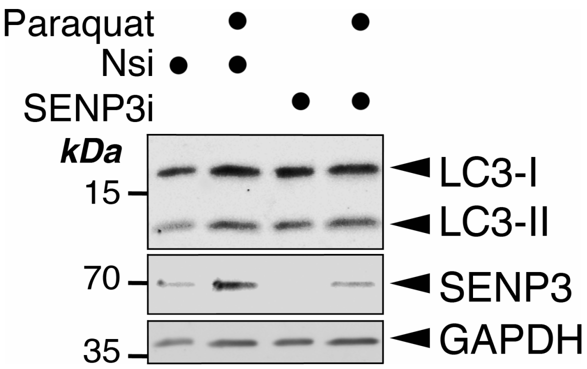

Appendix Figure S12. Absence of SENP3 appears to affect LC3-II induction by paraquat.

Nsi or SENP3i (I) was transfected into SH-SY5Y cells. Two days post-transfection the cells were treated with paraquat (10 μM) for 4 h. Whole cell lysate samples were blotted as indicated.

Mitophagic cell number/Total cell number						
Field	Nsi+PBS	Nsi+DFP	SENP3i+PBS	SENP3i+DFP	Nsi+PBS+CQ	Nsi+DFP+CQ
1	19/517	105/376	30/597	66/385	33/587	27/444
2	43/555	162/402	34/716	40/471	40/491	79/464
3	17/604	154/363	24/534	35/389	48/437	62/491

Appendix Table S1. SENP3 is required for DFP-induced mitophagy in living HeLa cells.

The table shows the raw numbers of mitophagic cells and total cells counted from three randomly chosen fields of view under the microscope, which were quantitative data used for plotting the histogram shown in **Figure 3D**.
