# Supplementary material for: The SUMO protease SENP3 regulates mitochondrial autophagy mediated by Fis1
Source: EMBO Rep. 2022 Jan 7;23(2):e48754. doi: 10.15252/embr.201948754 (PMC8811651; doi:10.15252/embr.201948754)
Supplement: Supplementary file 2 — Expanded View Figures PDF [file EMBR-23-e48754-s010.pdf]

Expanded View Figures

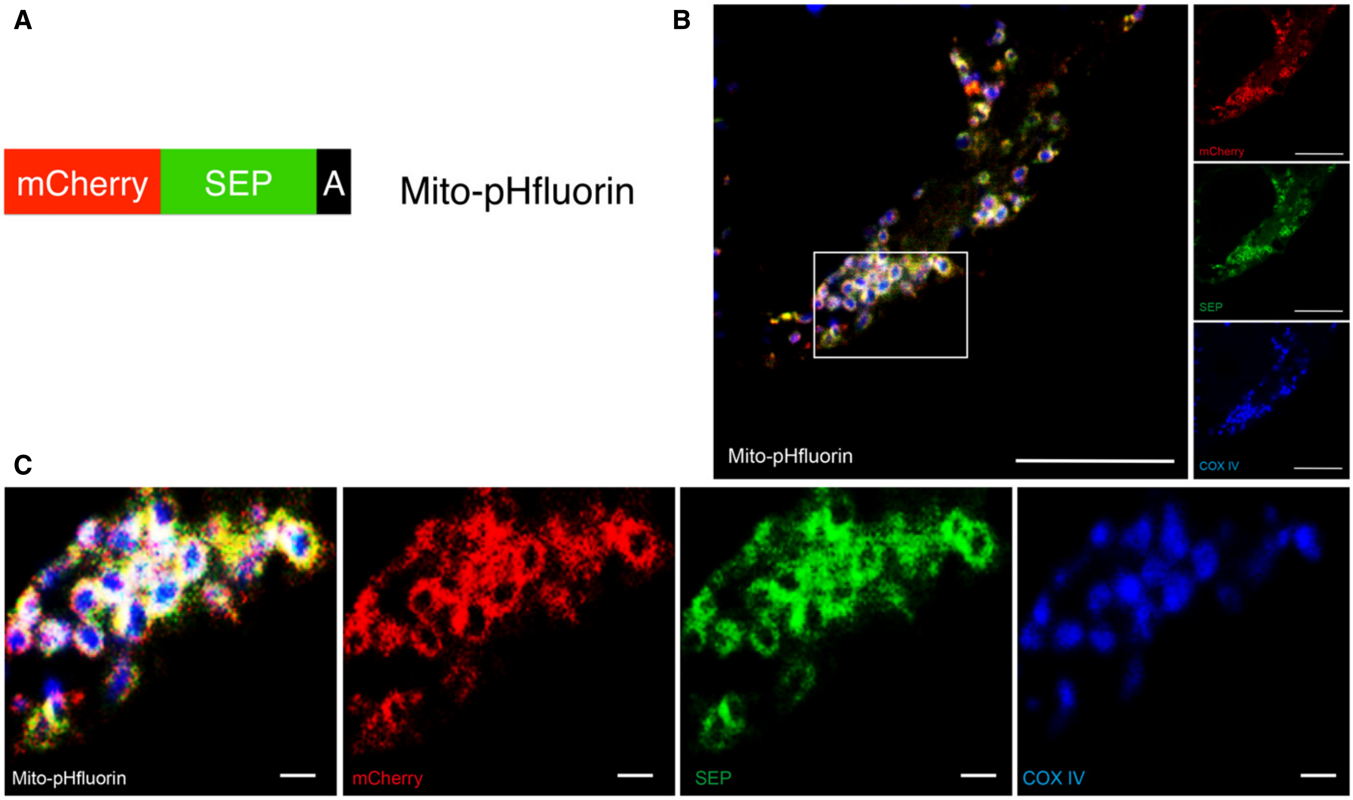

Figure EV1. Mito-pHfluorin outlines the mitochondria.

A Schematic illustrates Mito-pHfluorin, a tandem-tagged construct encoding mCherry-SEP-A (SEP, Super-ecliptic pHluorin (a pH-dependent GFP variant); A, the mitochondrial targeting sequence derived from the ActA protein fused to the C-terminus of SEP).

B Mito-pHfluorin was transfected into HeLa cells for 48 h. Cells were fixed with 4% PFA, and immunocytochemistry against the mitochondrial inner membrane protein COX IV was performed (Red: mCherry; Green: SEP; Blue: COX IV; Scale bar, 10 µm).

C Magnified views of the boxed area to show the co-localization of mito-pHfluorin (outer ring) and COX IV (inner circle) (Scale bar, 1 µm).

© 2022 The Authors

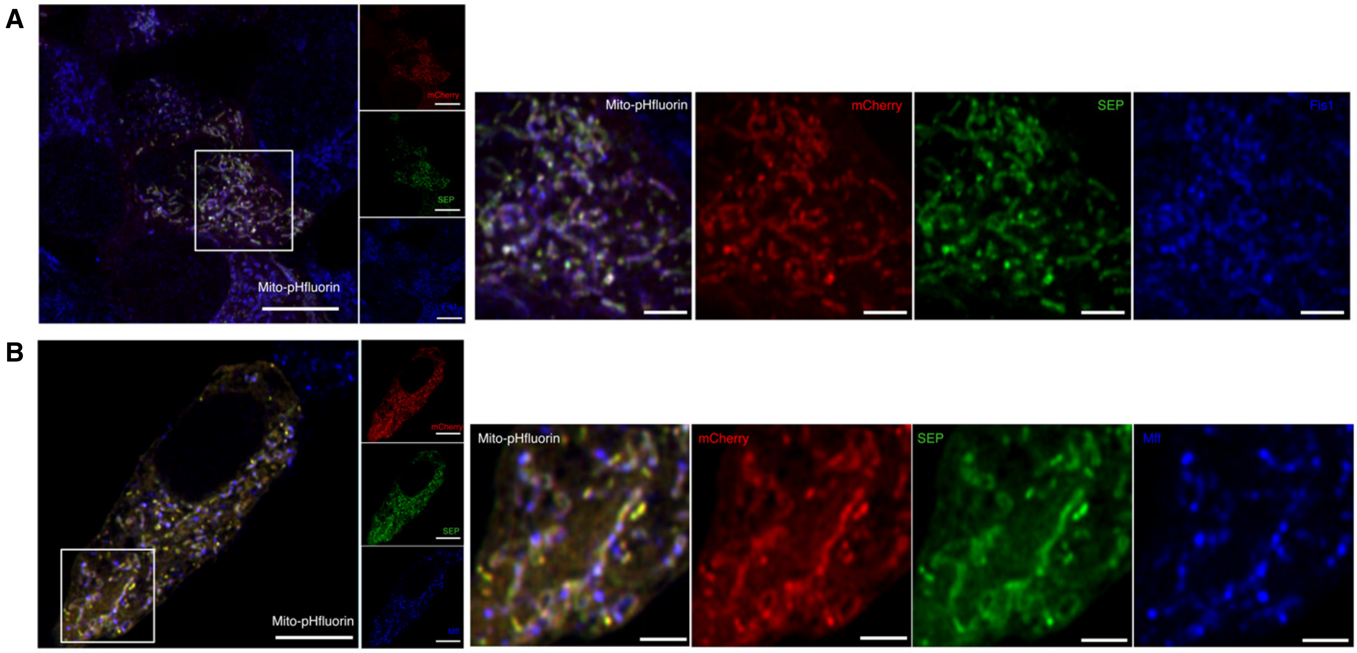

Figure EV2. Mito-pHfluorin is targeted to the mitochondria outer membrane containing Fis1 and Mff.

A Mito-pHfluorin was transfected into HeLa cells for 48 h. Cells were fixed with 4% PFA, and immunocytochemistry against the mitochondrial outer membrane protein Fis1 was performed (Left panel; Red: mCherry; Green: SEP; Blue: Fis1; Scale bar, 10 μm). Magnified views of the boxed area to show the co-localization of mito-pHfluorin and Fis1 (Light panel; Scale bar, 2.5 μm).

B Mito-pHfluorin was transfected into HeLa cells for 48 h. Cells were fixed with 4% PFA, and immunocytochemistry against the mitochondrial outer membrane protein Mff was performed (Left panel; Red: mCherry; Green: SEP; Blue: Mff; Scale bar, 10 μm). Magnified views of the boxed area to show the localization of Mff within the mitochondrial outer membrane outlined by mito-pHfluorin (Light panel; Scale bar, 2.5 μm).

 © 2022 The Authors

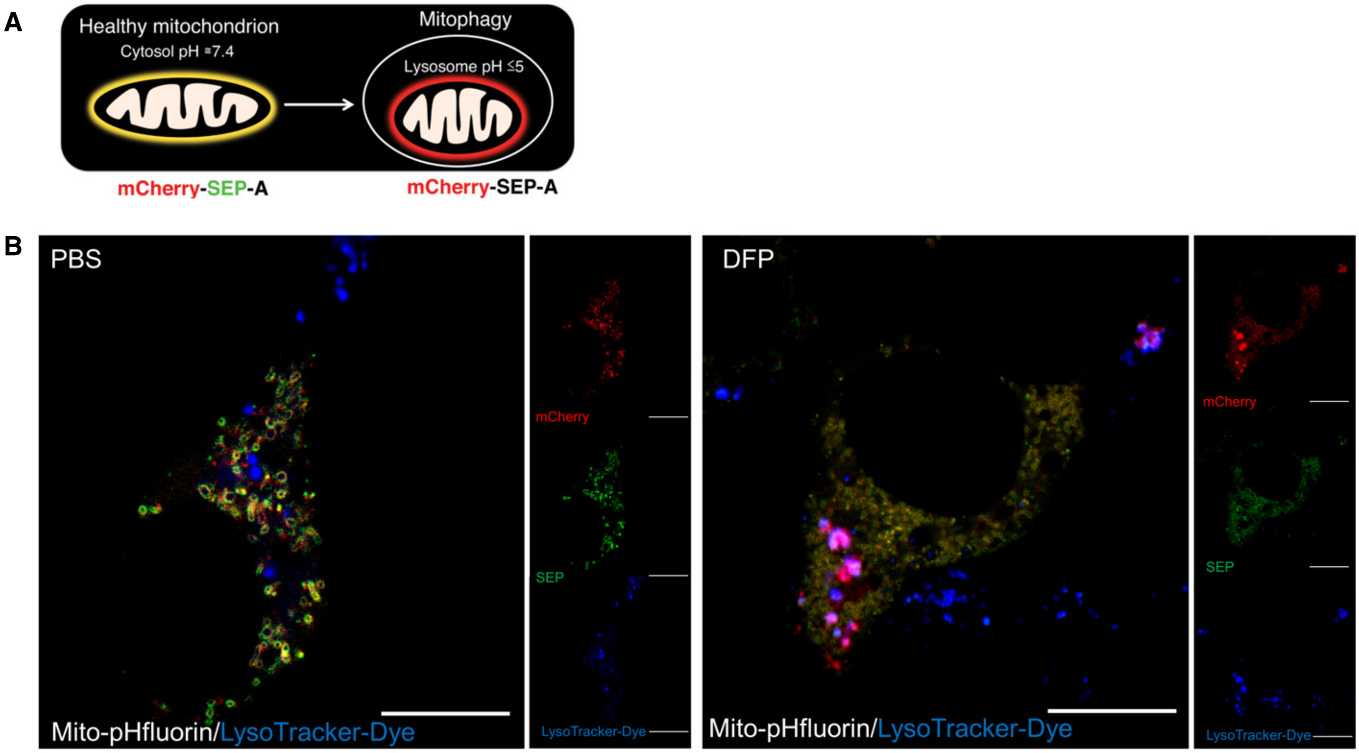

Figure EV3. Mito-pHfluorin is a probe for DFP-induced mitophagy.

A Schematic illustrates a mitophagy assay using the mito-pHfluorin construct. Under basal conditions, in green/red merged images mitochondria are labelled as yellow structures while upon mitophagy, mitochondria are sequestered within autolysosomes where fluorescence of SEP is quenched due to the acidic environment and therefore outlined as red structures.

B Co-localization of red-alone puncta detected by mito-pHfluorin and lysosomes stained by LysoTracker (Red: mCherry; Green: SEP; Blue: lysosomes; Scale bar, 10 μm).

© 2022 The Authors

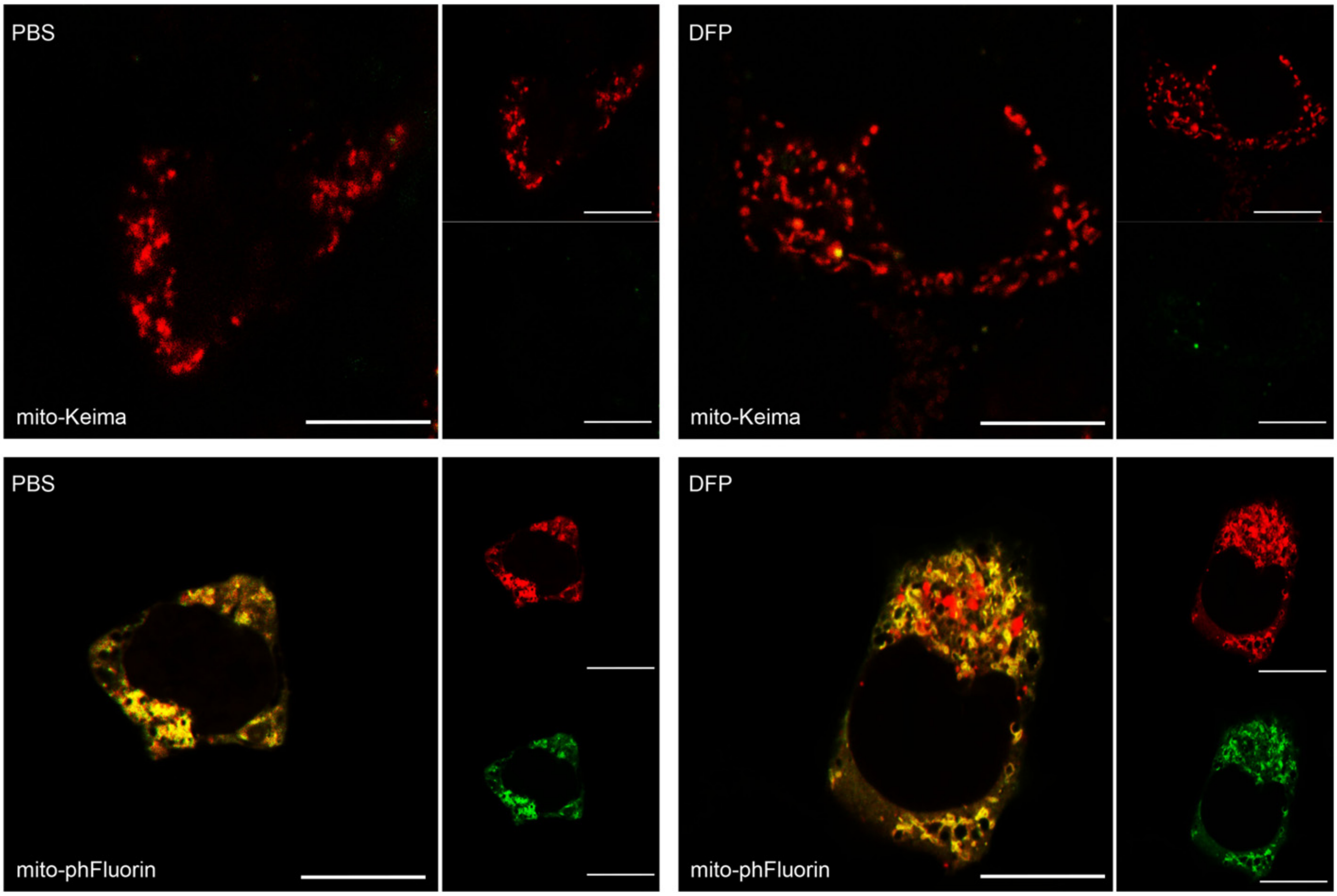

Figure EV4. Mito-pHfluorin is suitable for detecting DFP-induced mitophagy in fixed HeLa cells.

Mito-Keima or mito-pHfluorin were transfected into HeLa cells for 48 h. Cells were treated with either PBS or DFP (1 mM; 24 h) and fixed with 4% PFA prior to imaging analysis. Images of mito-Keima under control (PBS) or DFP treatment conditions are shown in the upper panel (Red: Mito-Keima; Green, residual green fluorescence emission from Mito-Keima; Scale bar, 10 μm). Images of mito-pHfluorin under the two conditions are shown in the lower panel. Red: mCherry; Green, SEP; Scale bar, 10 μm. In mito-pHfluroin-expressing cells, mitochondria are visualized in the merged images as yellow structures, and red, SEP-quenched, puncta are present in DFP-treated cells.

 © 2022 The Authors

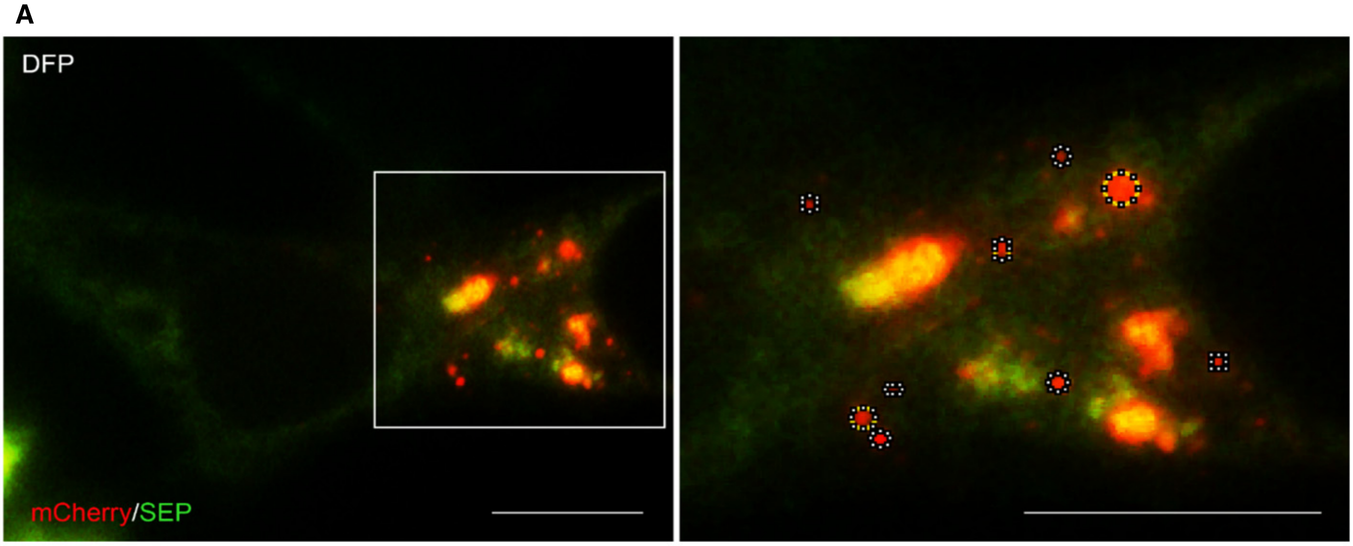

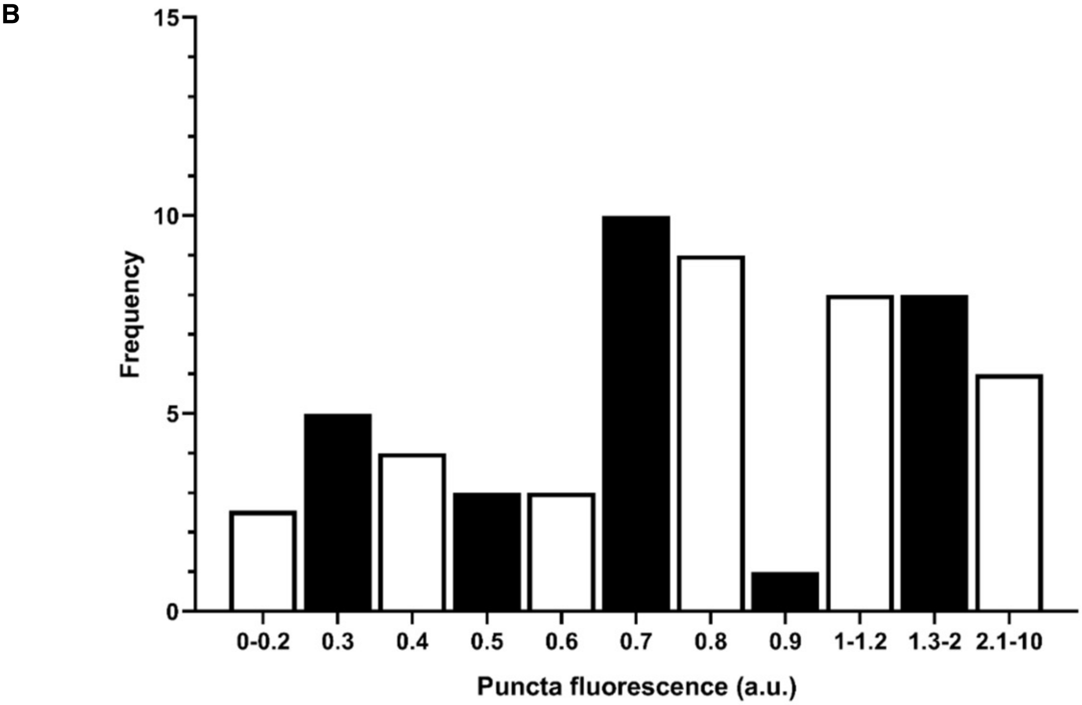

Figure EV5. Criteria used for quantifying mitophagy using Mito-pHfluorin.

A Mito-pHfluorin was transfected into HeLa cells for 48 h. DFP (1 mM; 24 h) was then added to induce mitophagy. The left panel shows a representative image, displaying the heterogeneity of the autolysosome population in a DFP-treated cell. Using ImageJ software, autolysosome intensity was measured by drawing around the red puncta (the right panel showing the boxed area of interest in the left panel; dotted circles; Scale bar, 10 μm).

B A frequency histogram showing that the intensities of the autolysosomes range considerably, from ≤ 0.2 A.U to 10 A.U: quantification of the frequency of red puncta intensity ($n = 44$) with a median determined to be 0.7 A.U. This was taken as the threshold for an autolysosome. The median was chosen to better reflect the central tendency and reduce the effect that outliers may have. In addition, the mode, as depicted in the frequency histogram as the highest peak, was also 0.7 A.U, suggesting that 0.7 A.U is frequent intensity for the red puncta and is likely to represent autolysosomes. Any autolysosomes detected that were double this size (1.4 A.U) were counted as two autolysosomes etc. Together, this confirms the heterogeneity of autolysosomes and establishes a criterion for autolysosome detection by mito-pHfluorin.

© 2022 The Authors
