# Supplementary figures and images for: The SUMO protease SENP3 regulates mitochondrial autophagy mediated by Fis1
Source: EMBO Rep. 2022 Jan 7;23(2):e48754. doi: 10.15252/embr.201948754 (PMC8811651; doi:10.15252/embr.201948754)

Figure 1

A

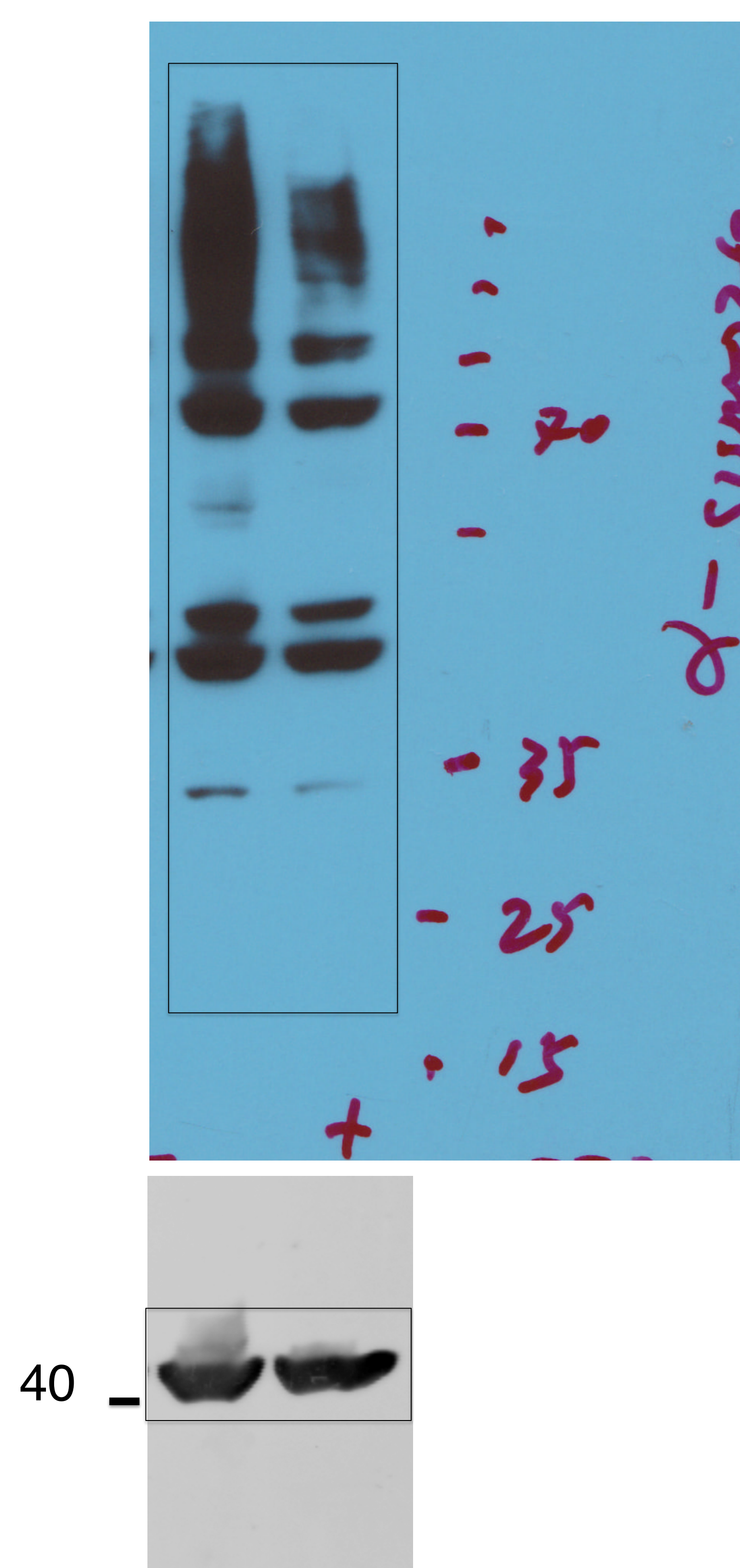

B

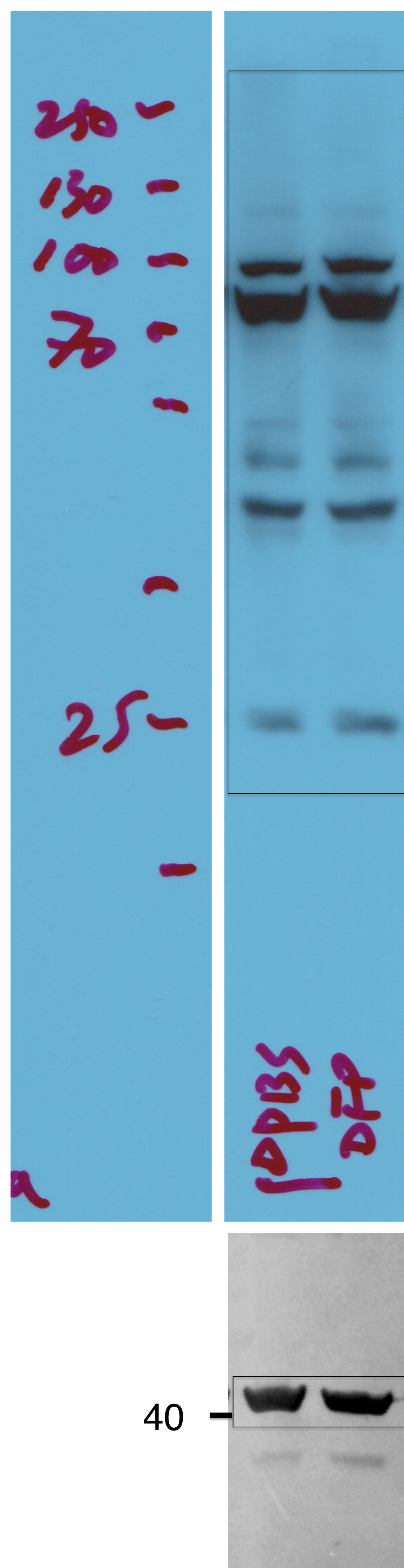

C

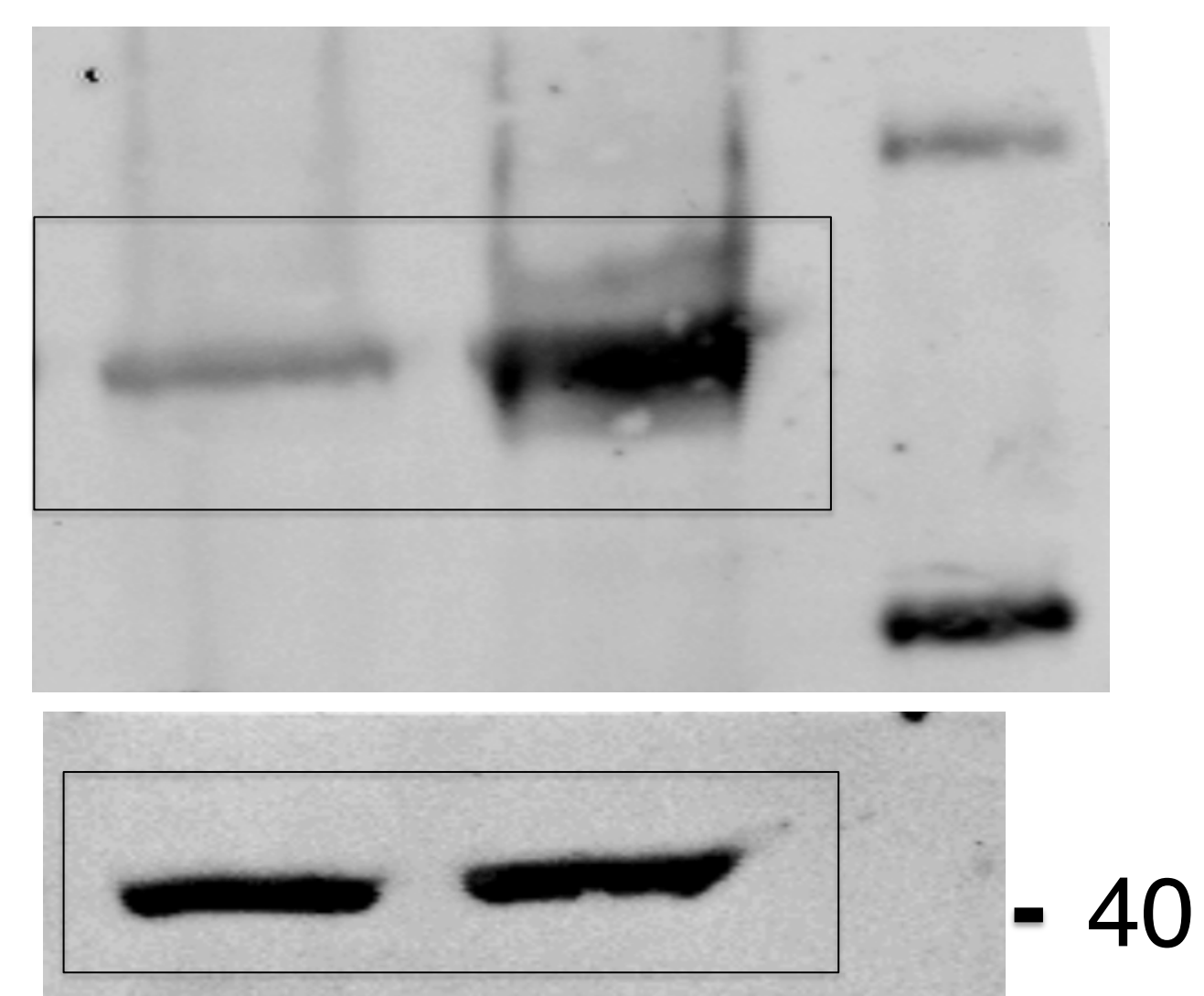

D

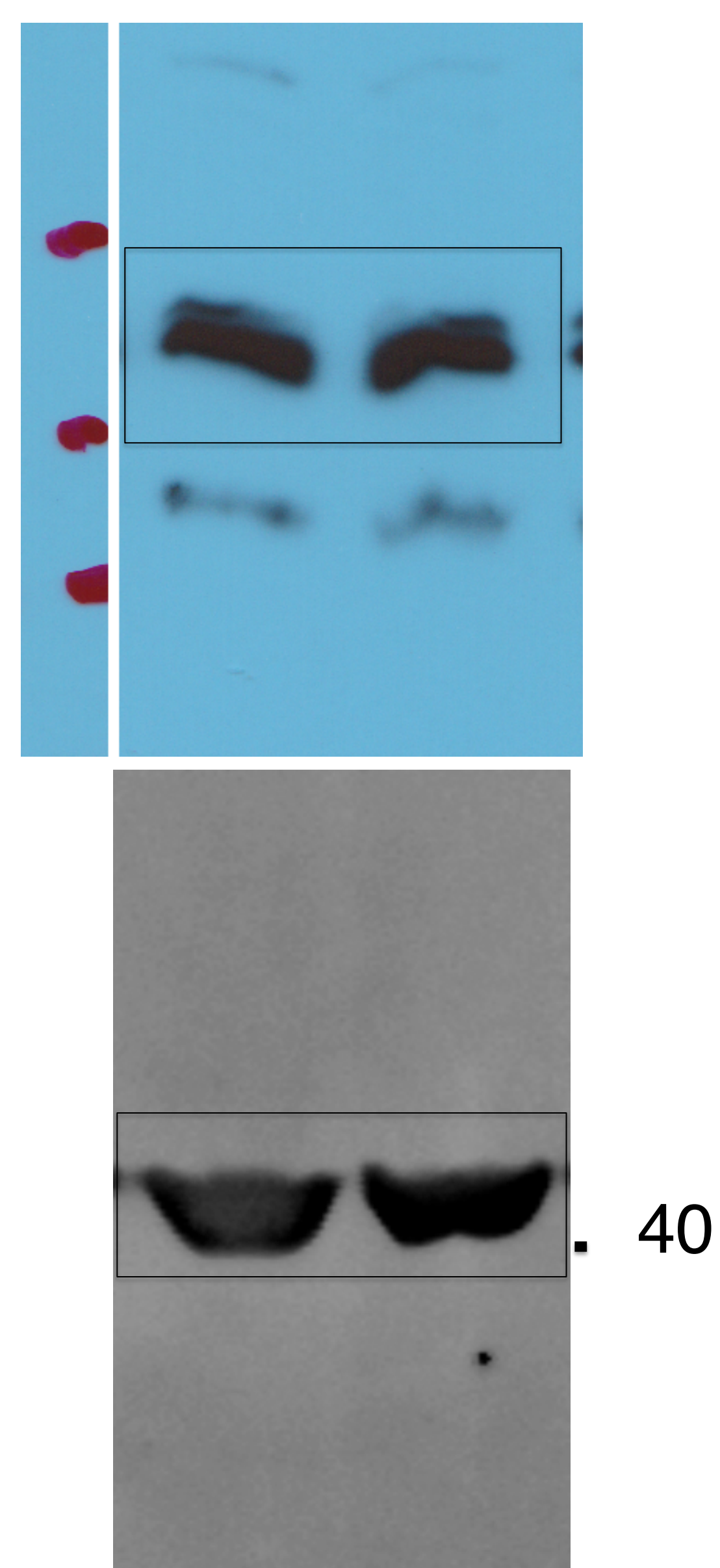

F

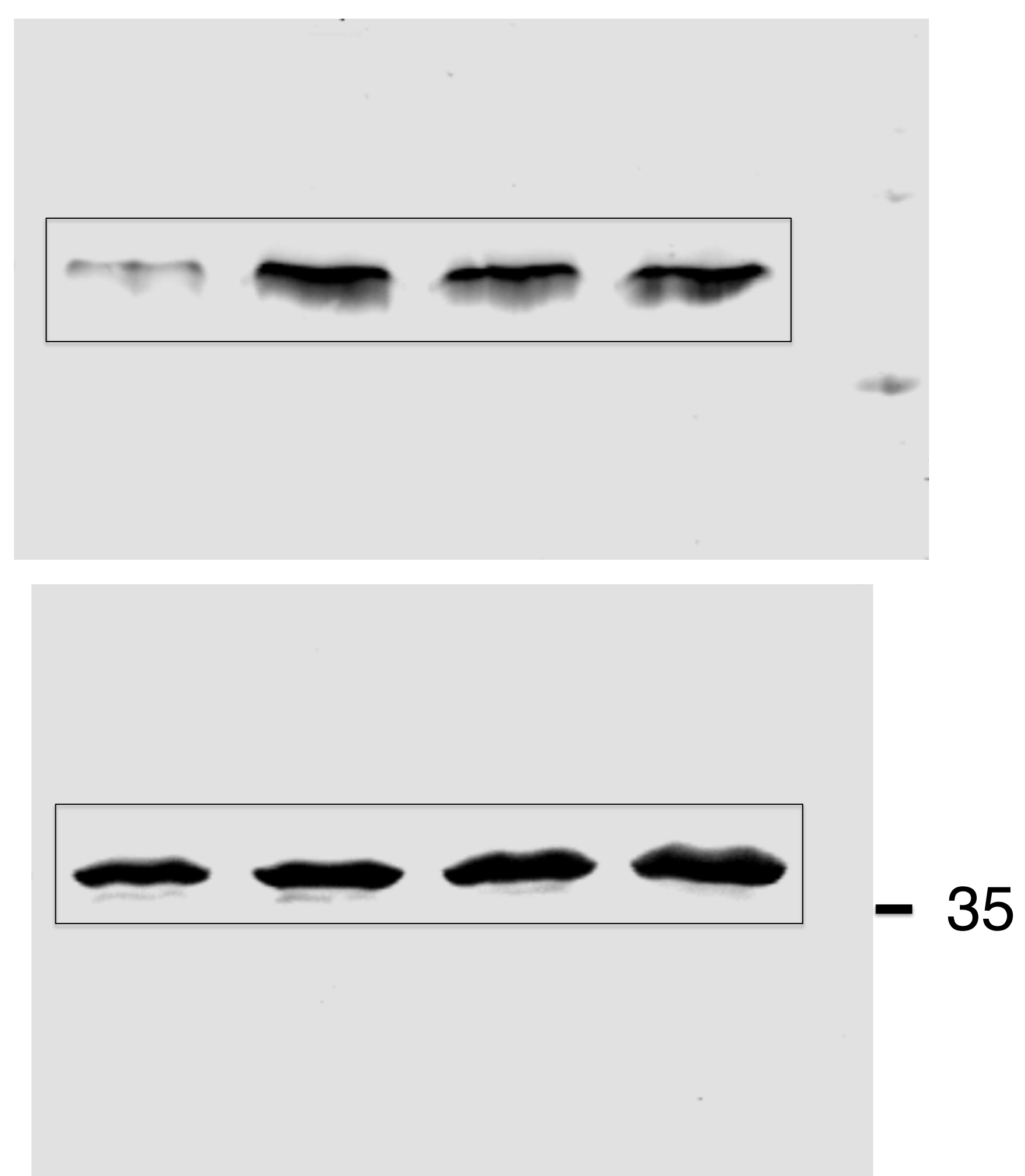

G

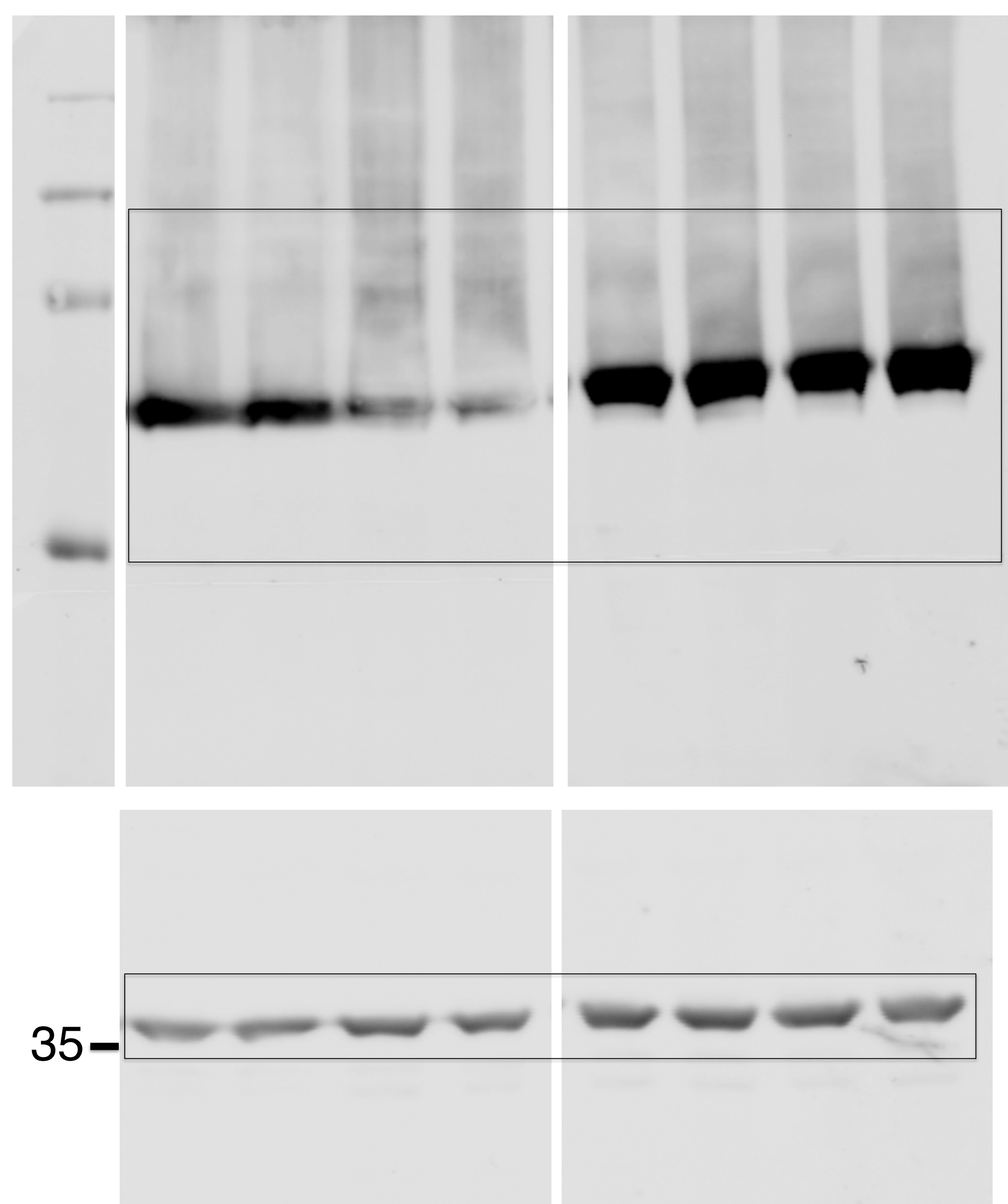

Supplement: Supplementary file 3 — Source Data for Figure 1 [file EMBR-23-e48754-s005.pdf]

**Figure 2**

**A**

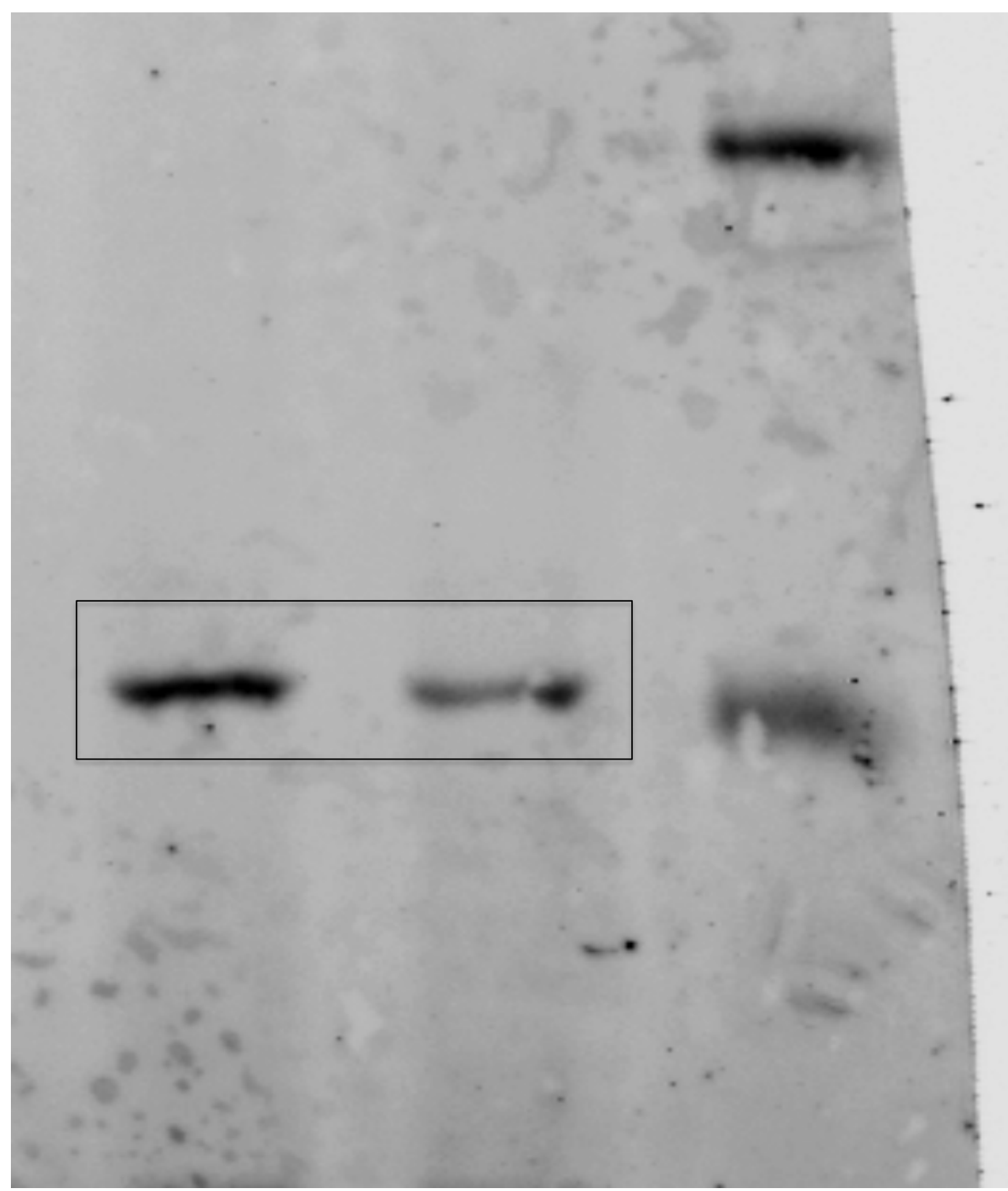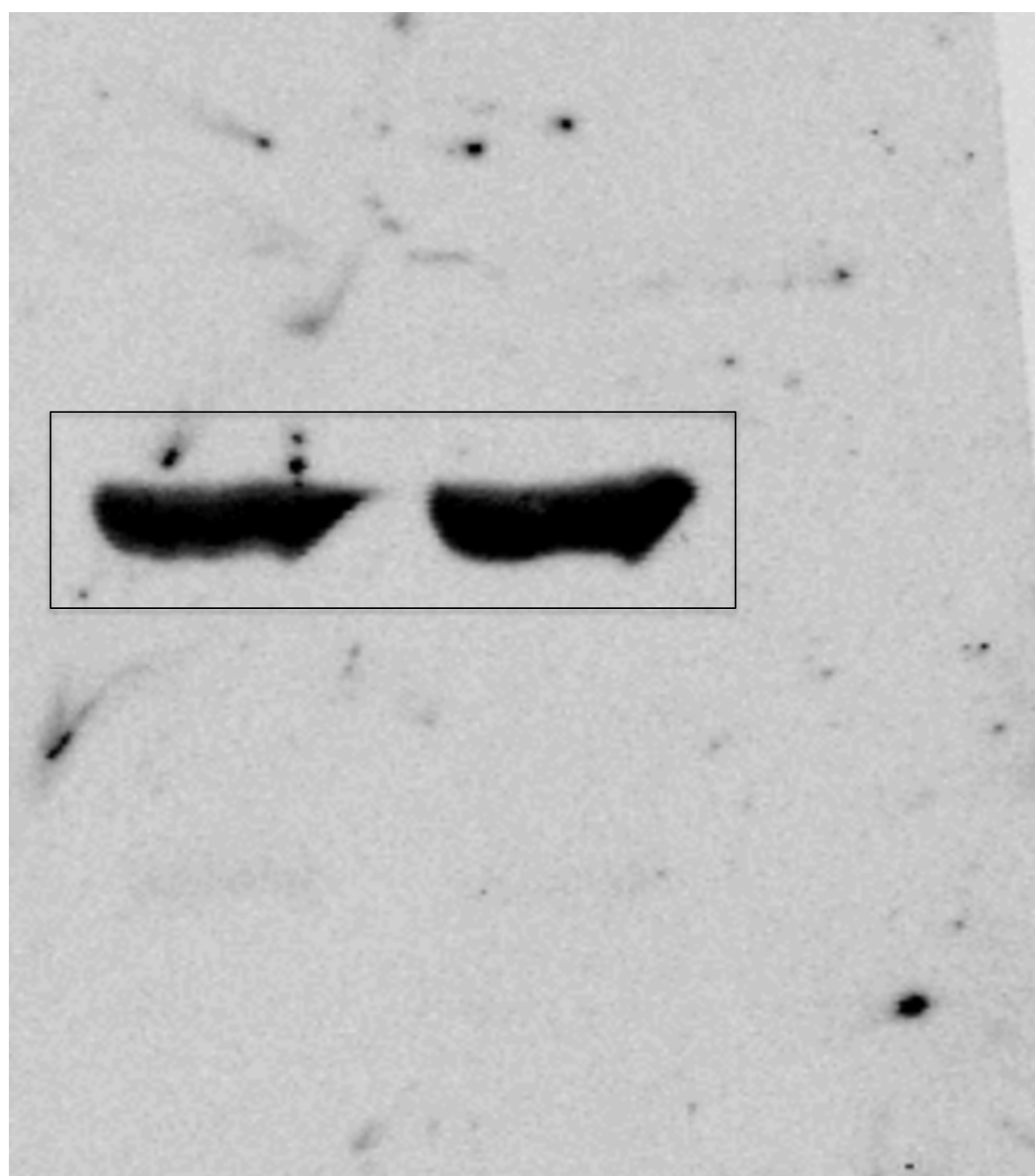

**C**

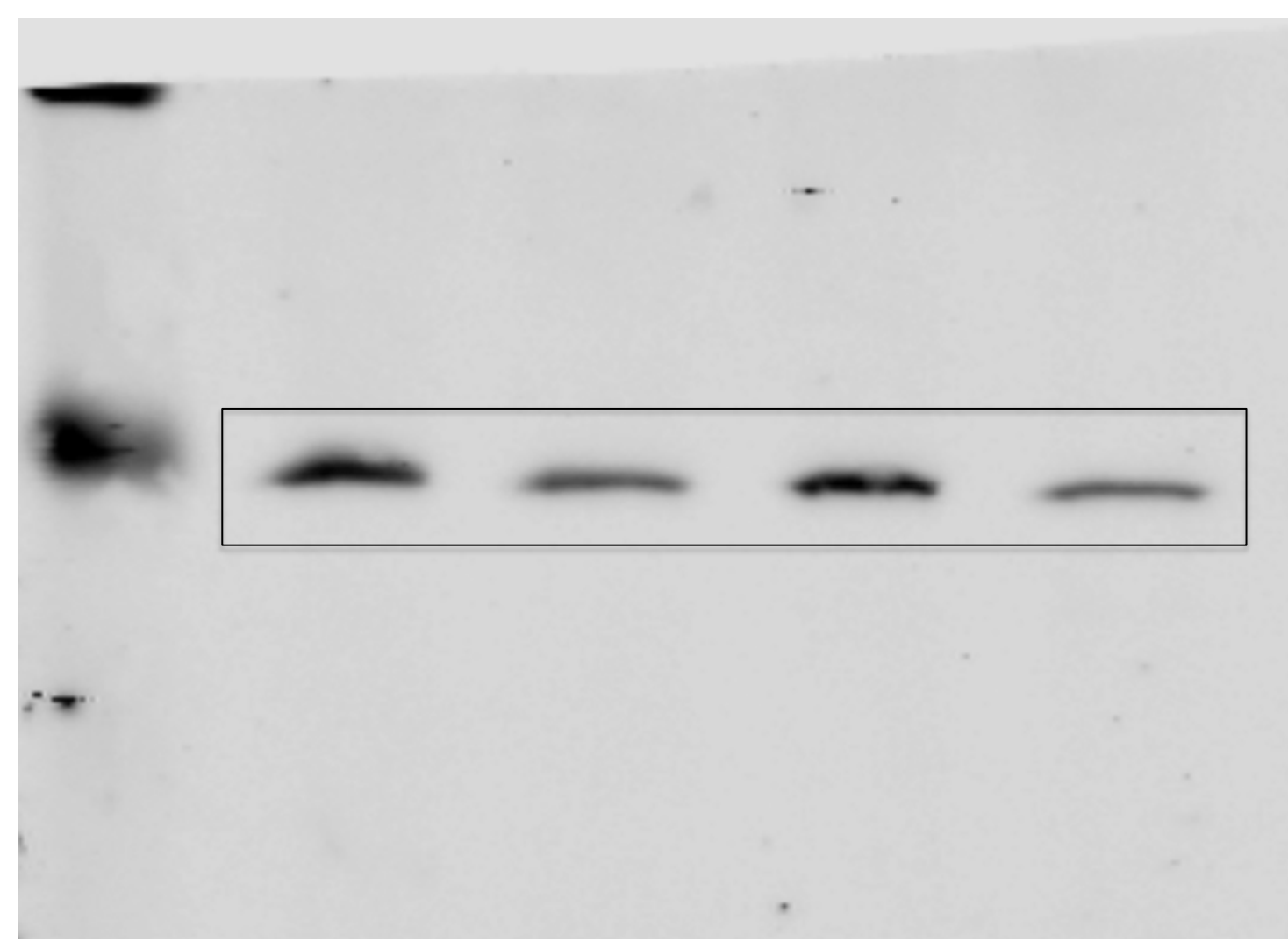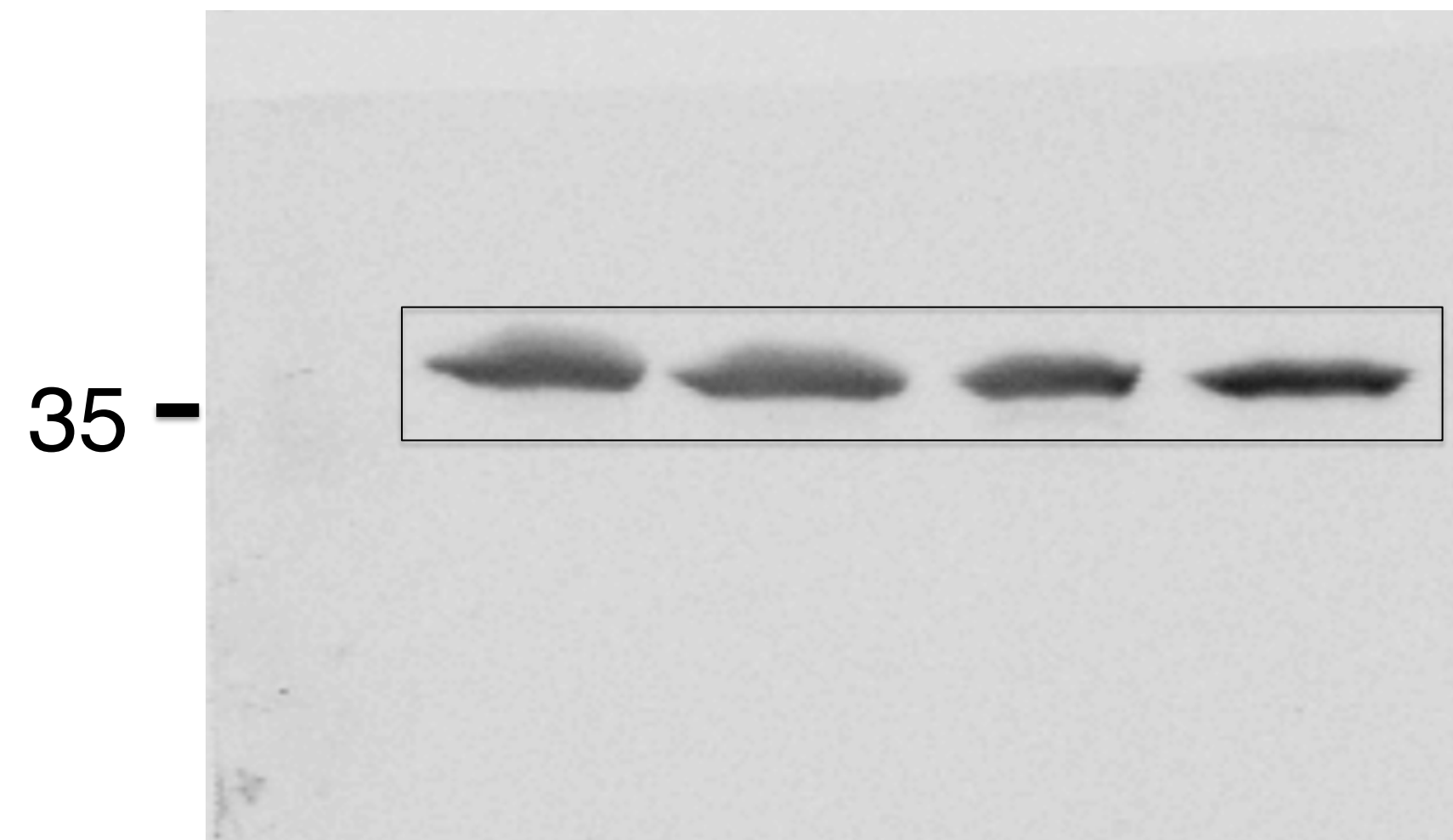

**D**

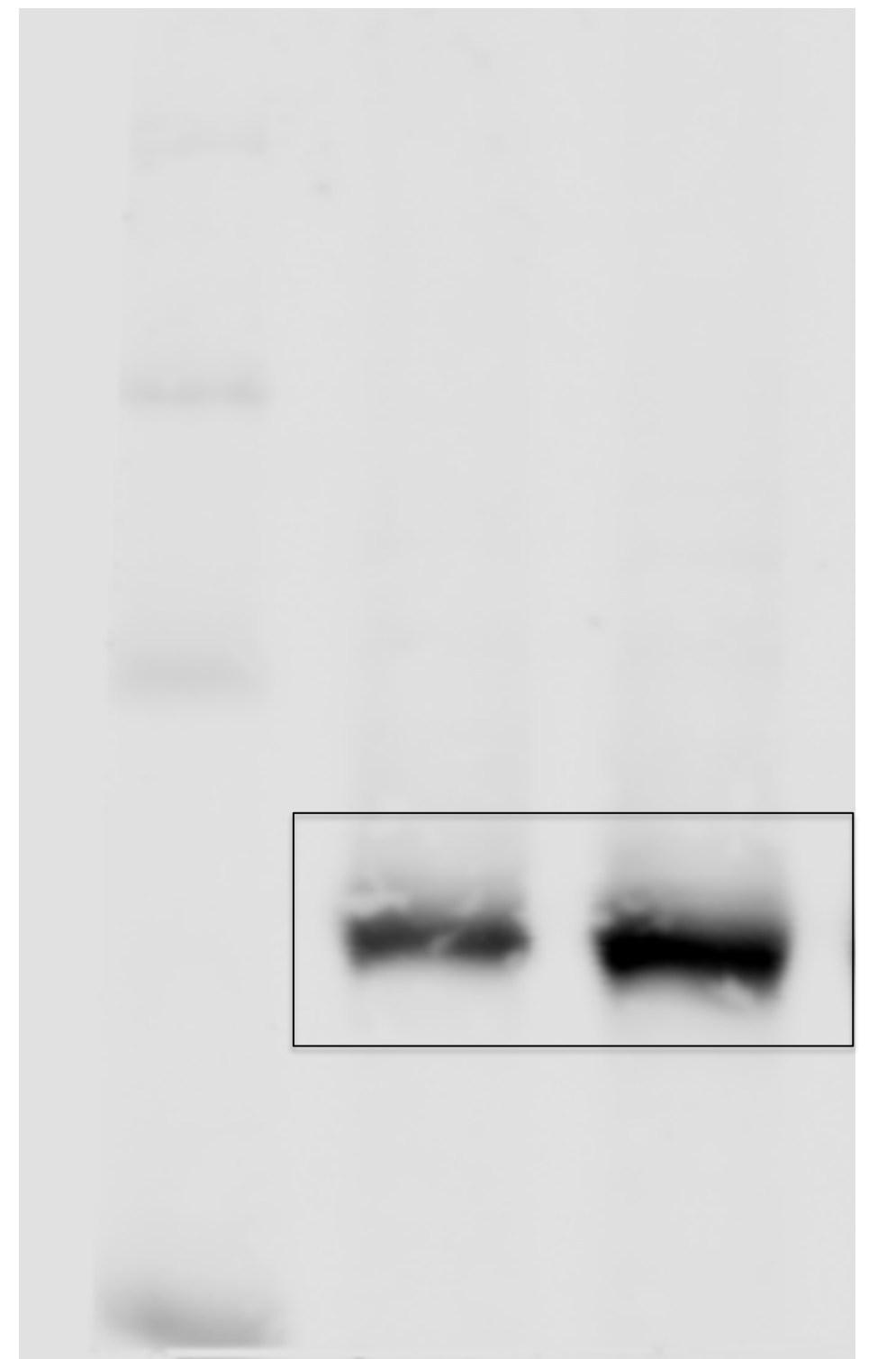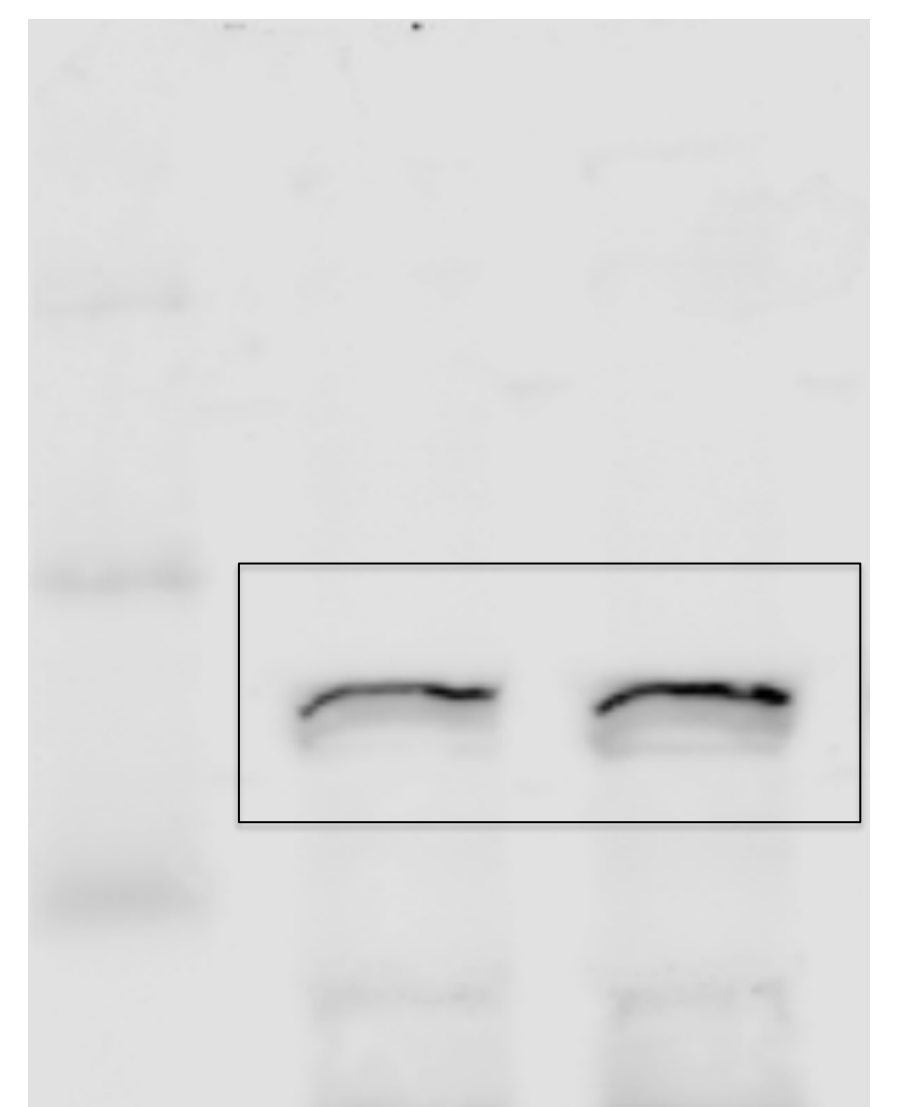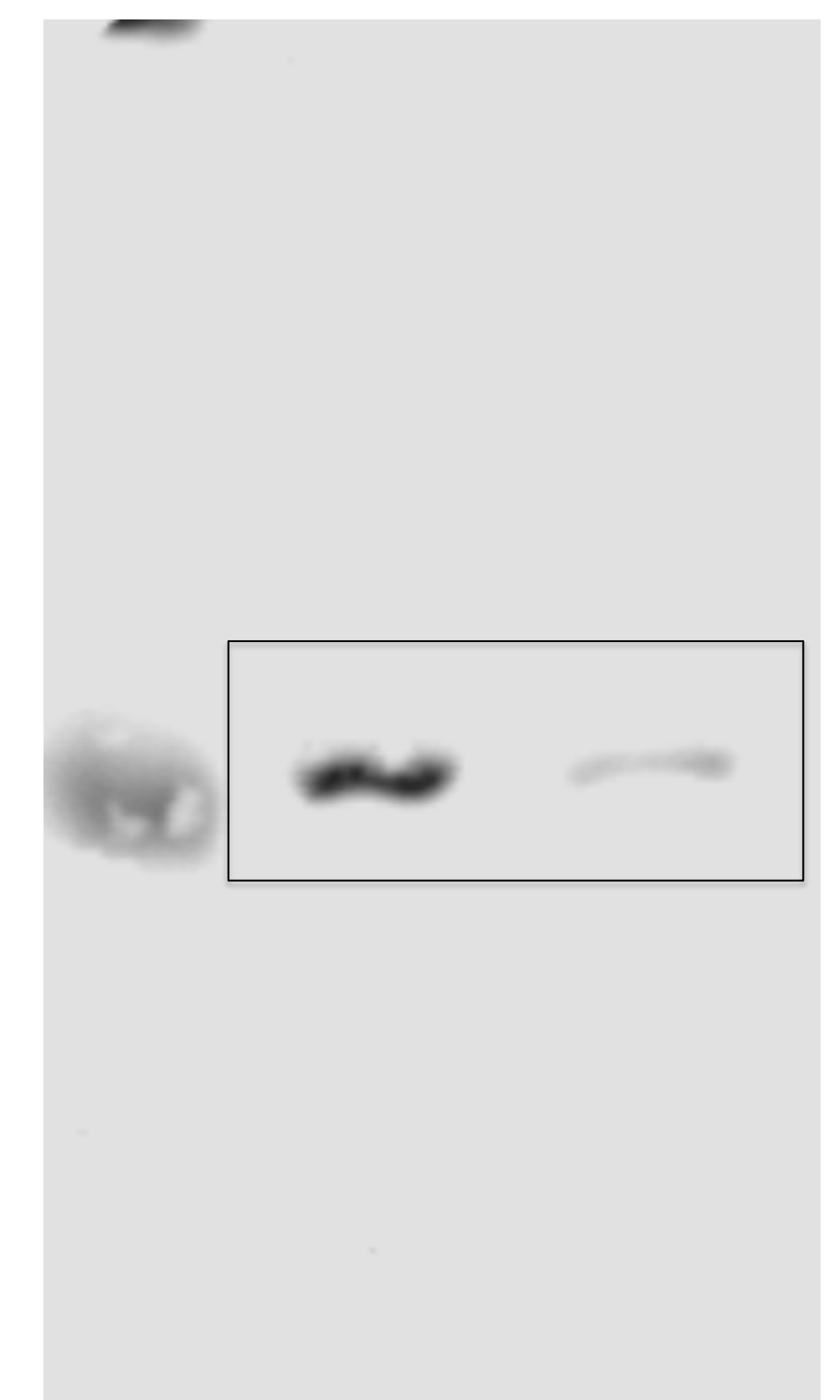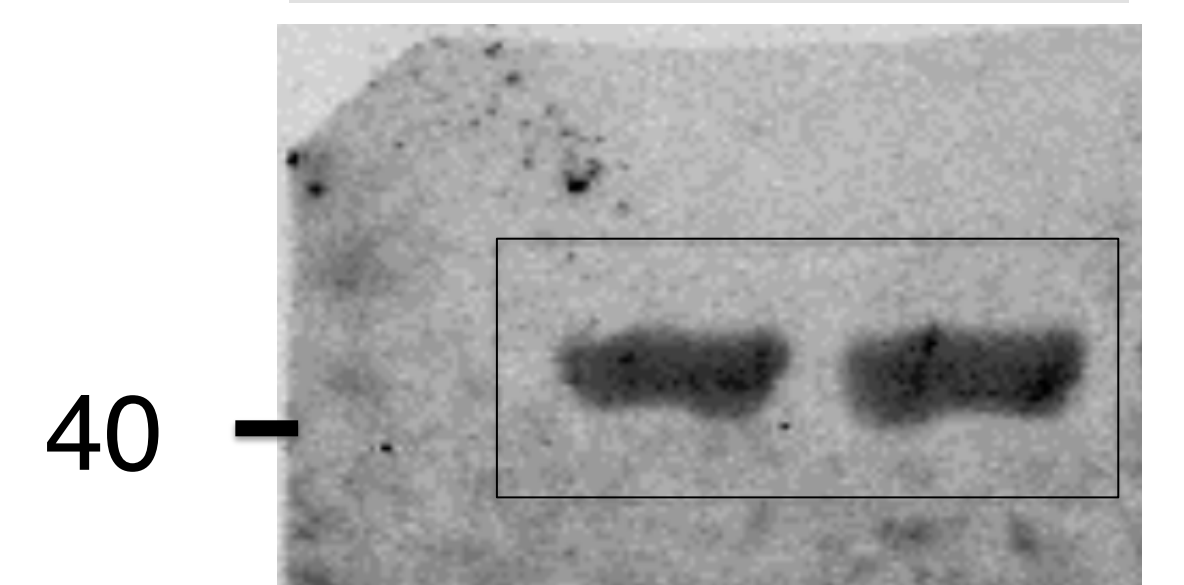

**E**

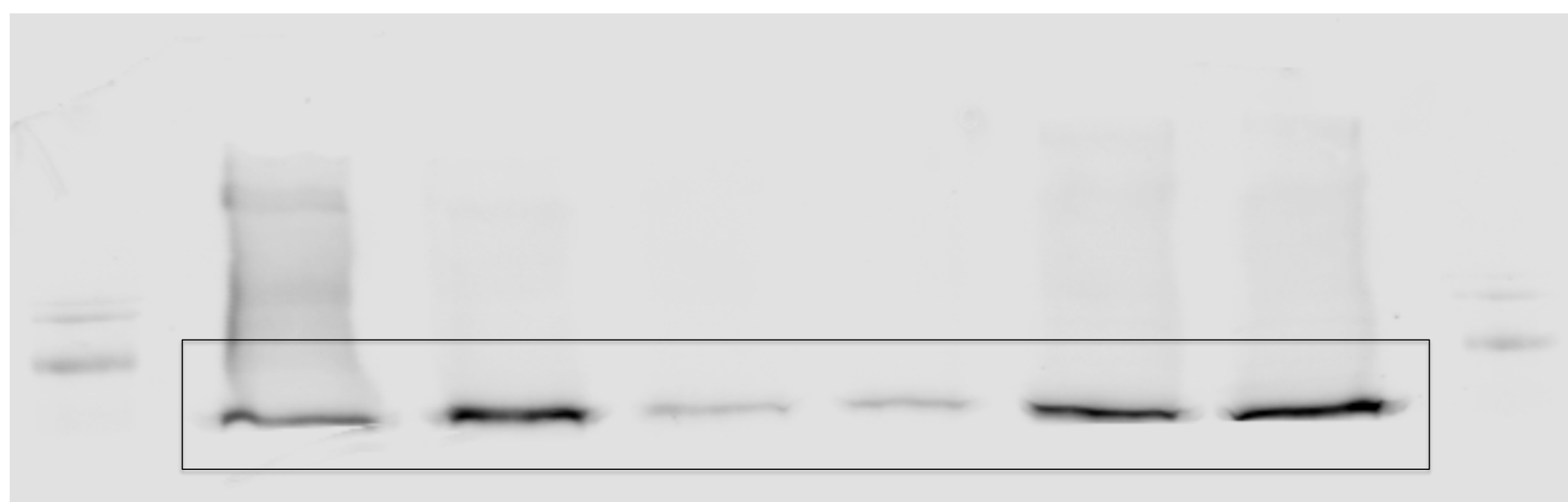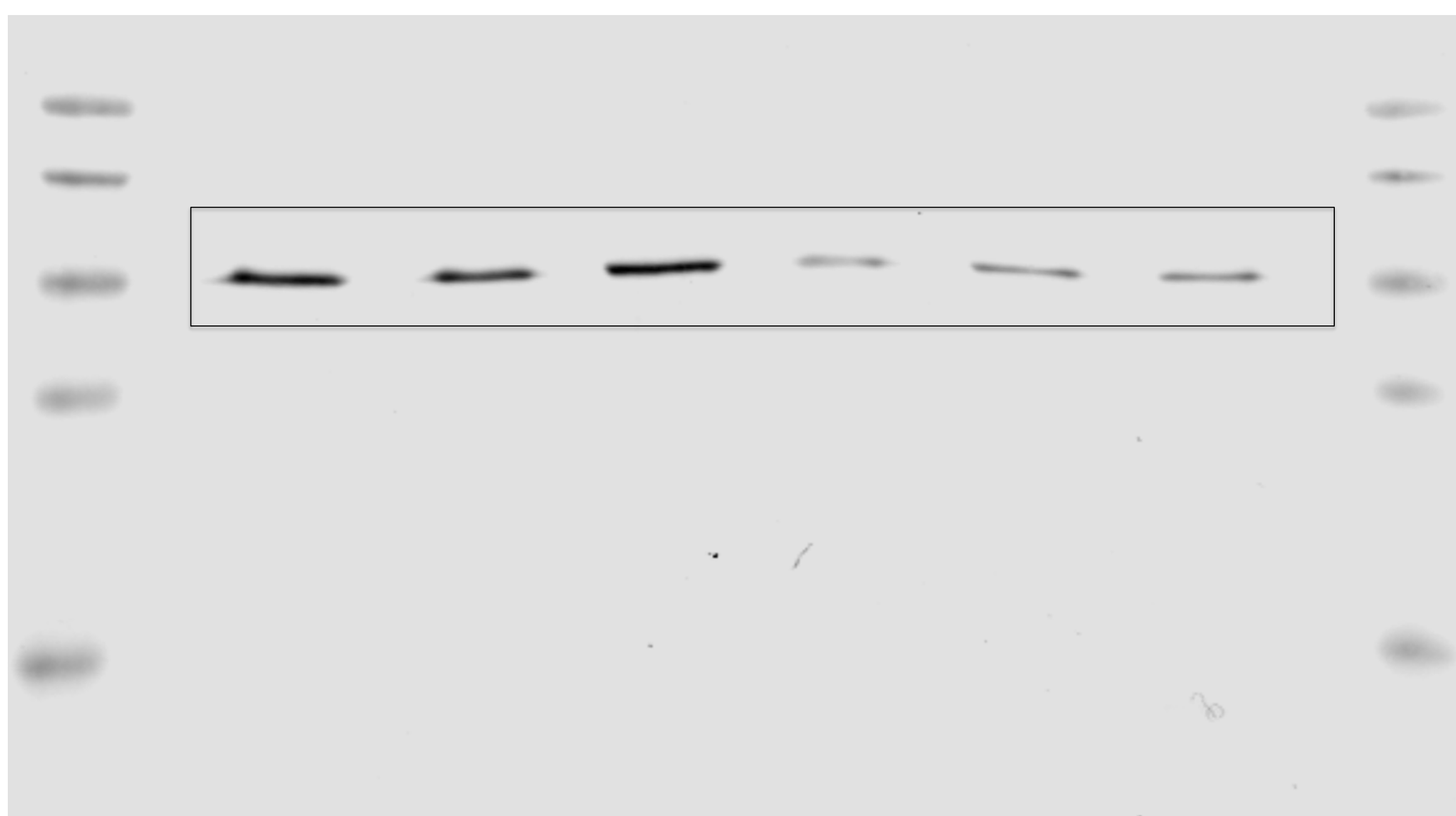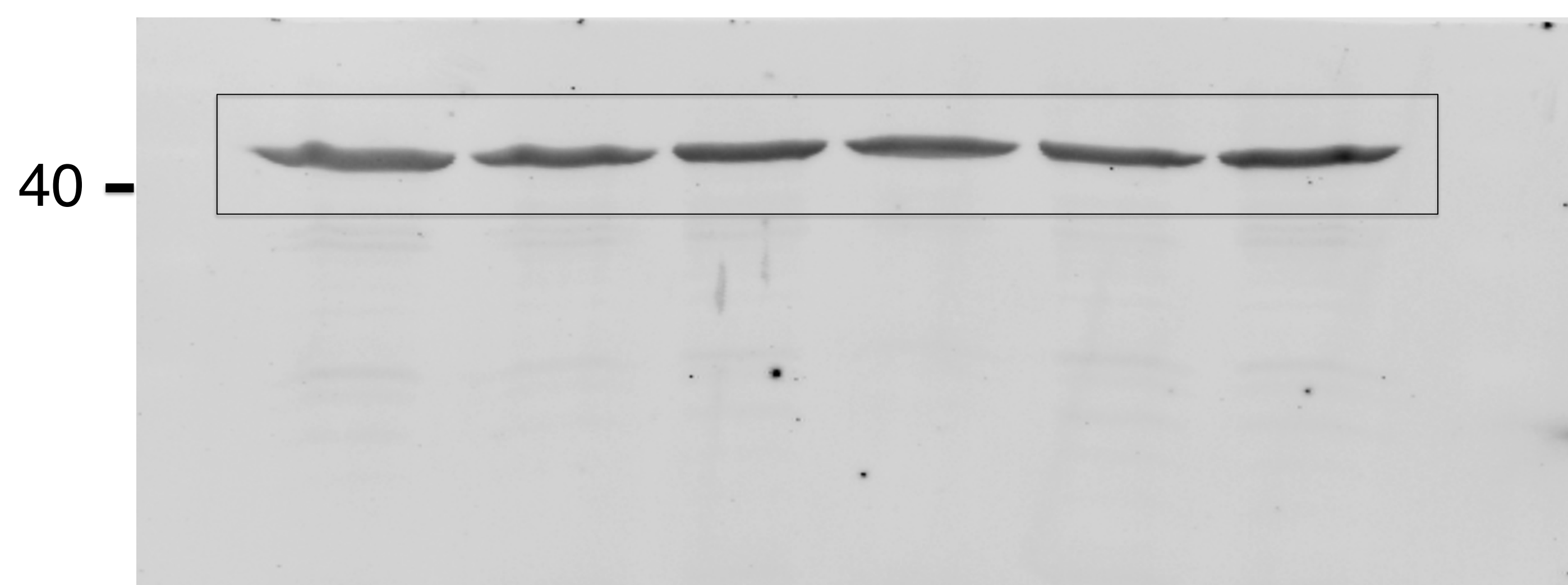

Supplement: Supplementary file 4 — Source Data for Figure 2 [file EMBR-23-e48754-s008.pdf]

Figure 3

A

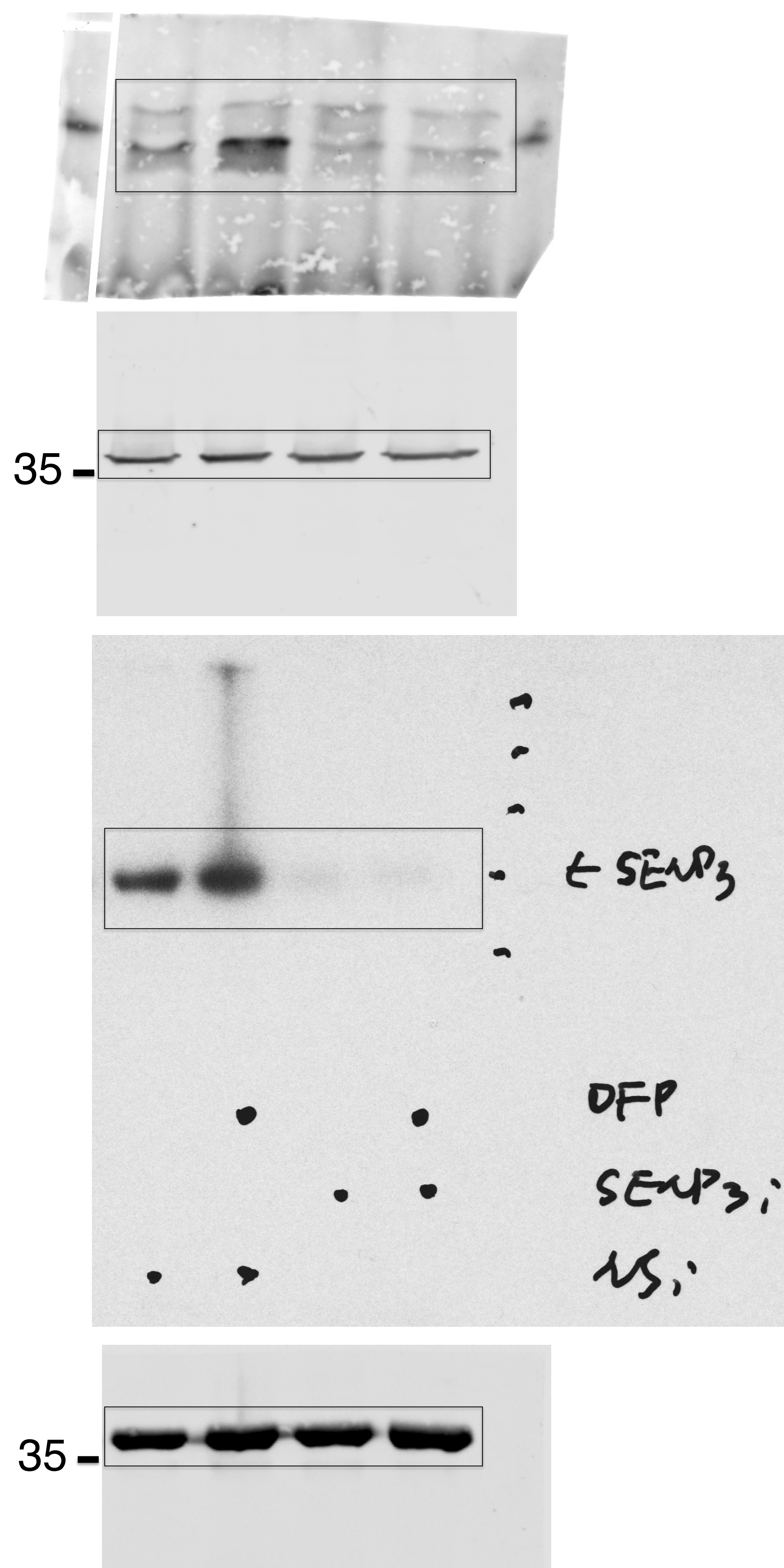

B

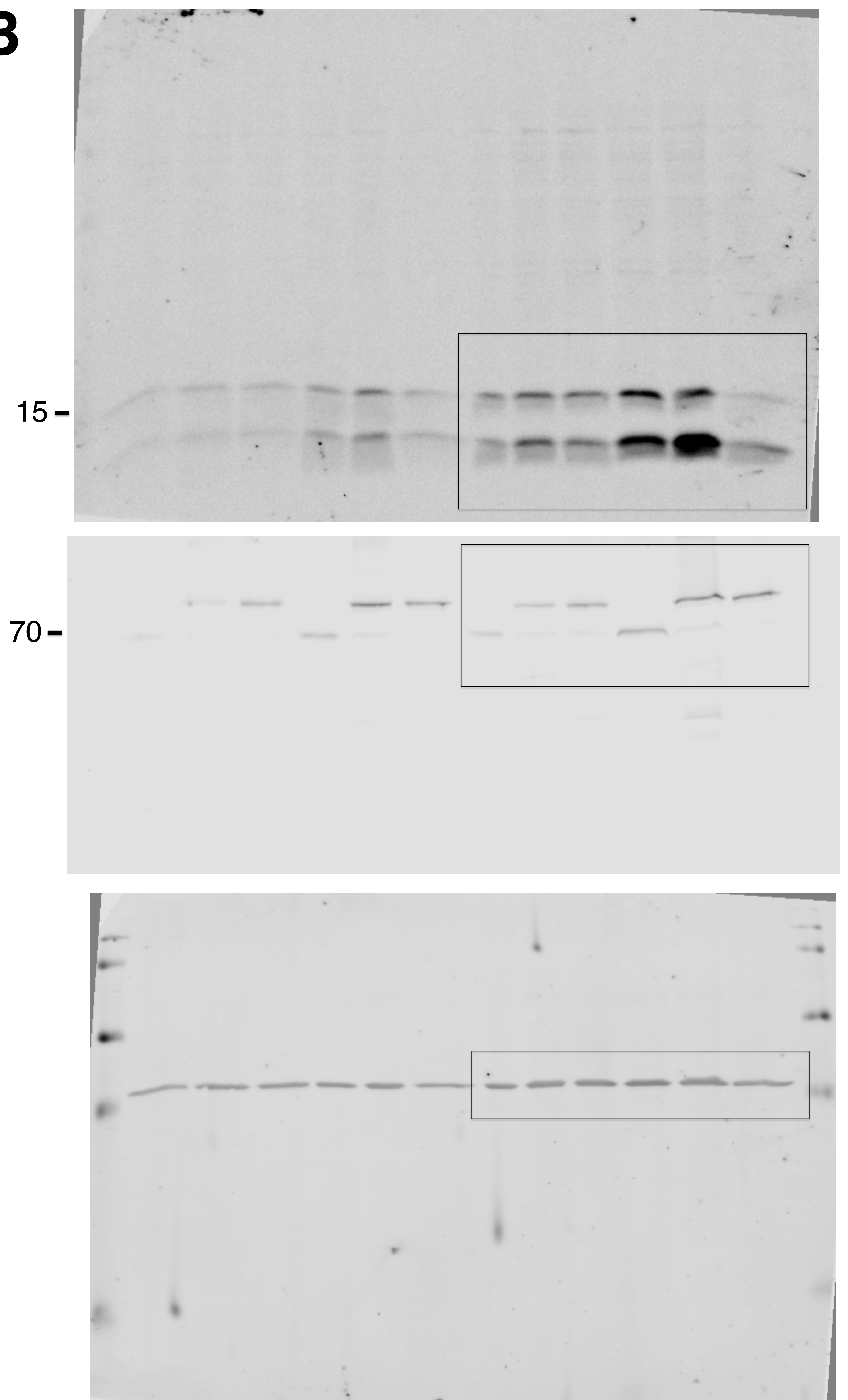

Supplement: Supplementary file 5 — Source Data for Figure 3 [file EMBR-23-e48754-s003.zip › Figure3_Source_Data/Raw Blots for EMBO Reports Figure 3.pdf]

Figure 4

**A**

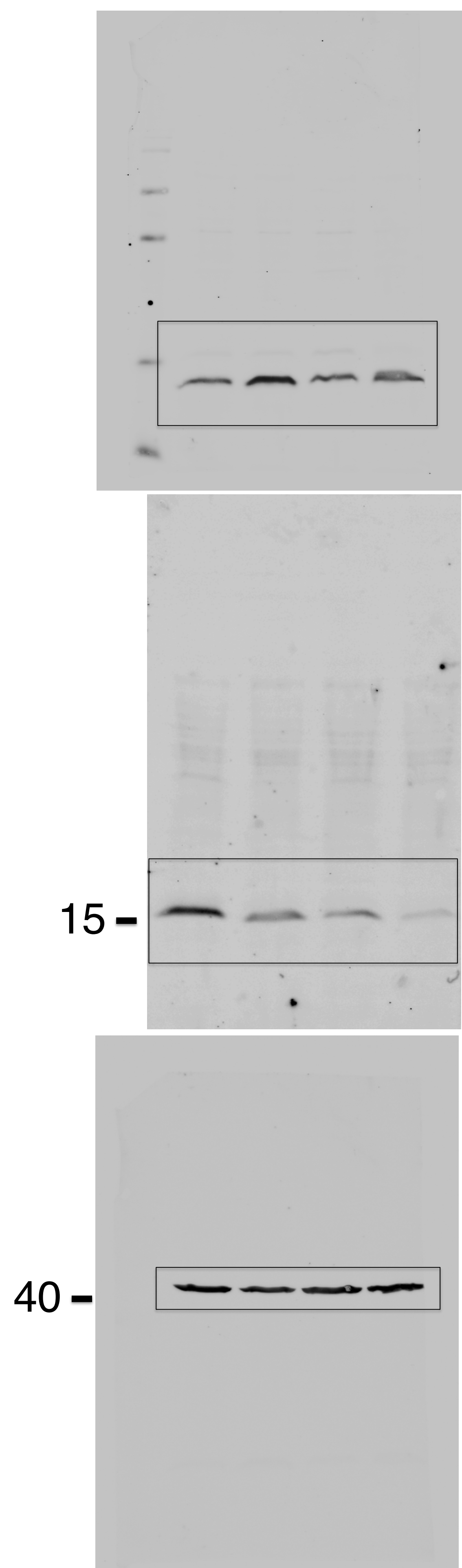

**B**

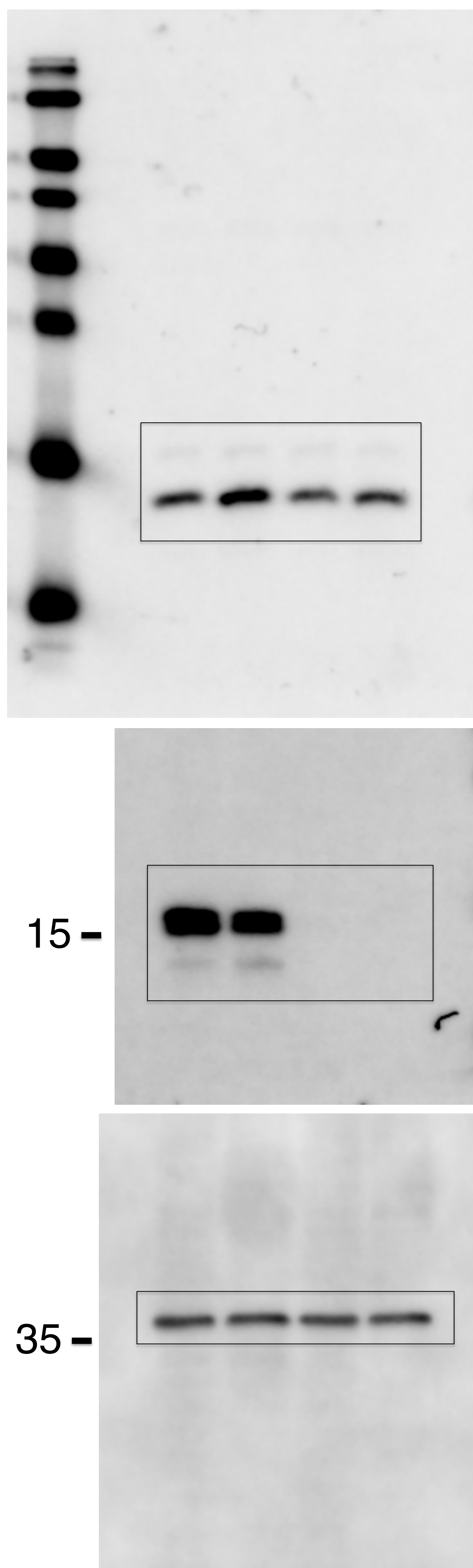

**C**

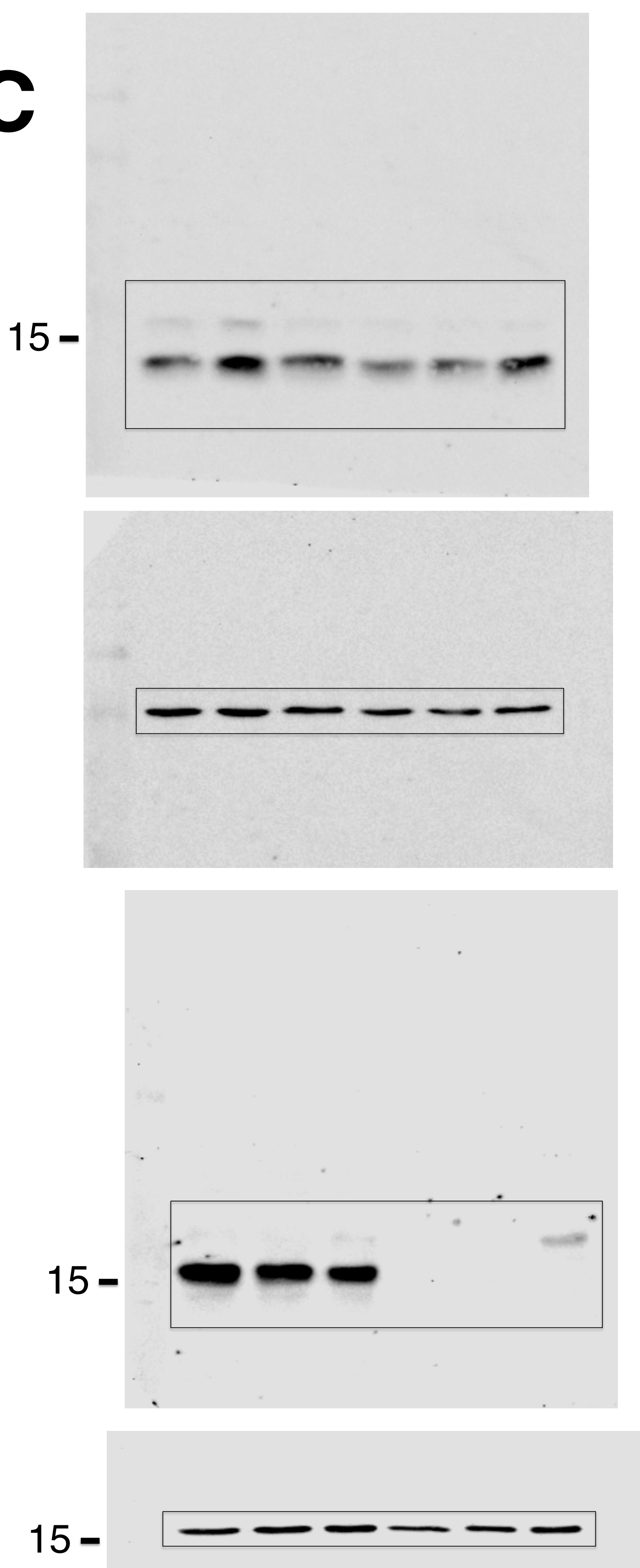

Supplement: Supplementary file 6 — Source Data for Figure 4 [file EMBR-23-e48754-s001.zip › Figure4_Source_Data/Raw_Blots_for_EMBO_Reports_Figure_4.pdf]

Figure 5

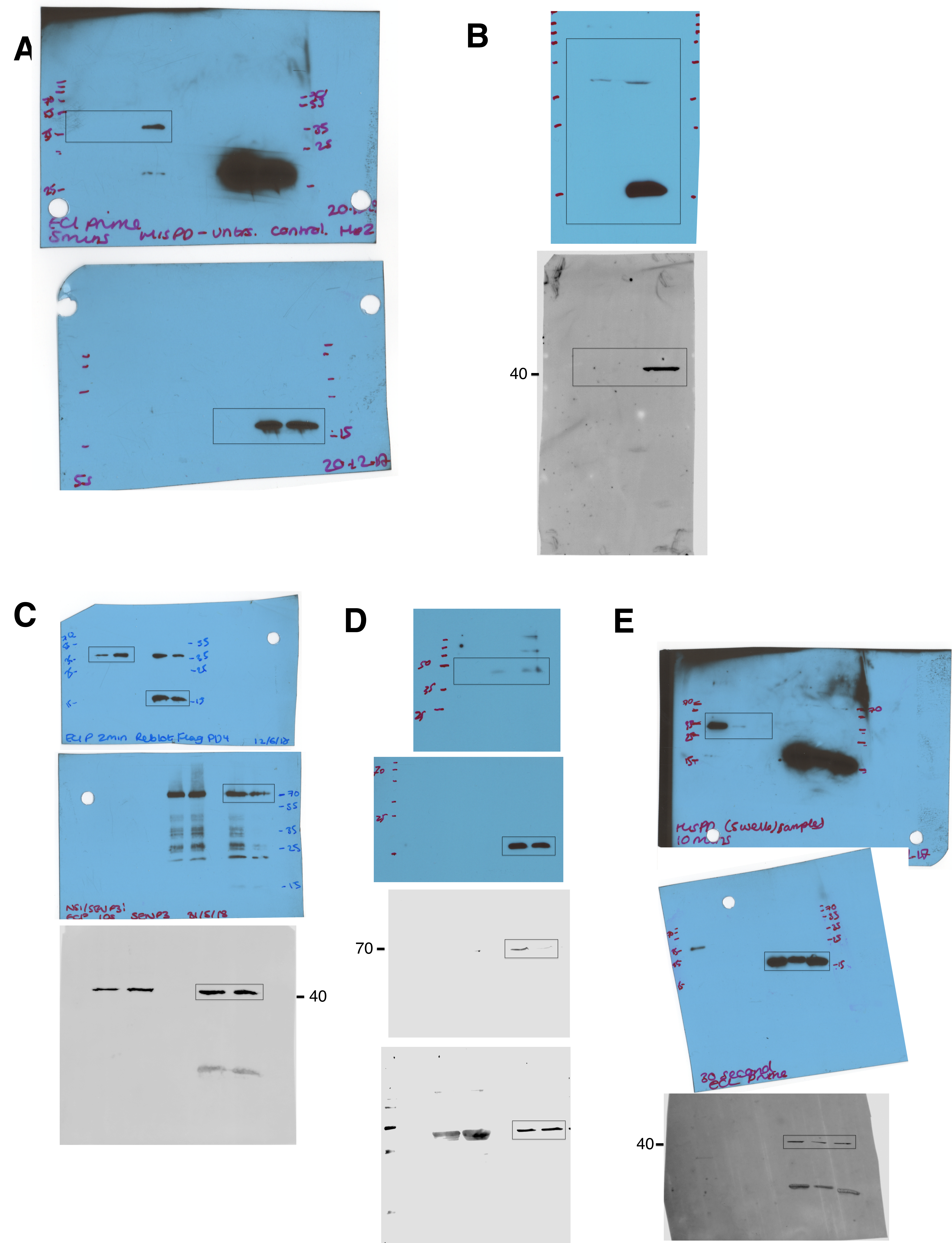

Supplement: Supplementary file 7 — Source Data for Figure 5 [file EMBR-23-e48754-s002.pdf]

**Figure 6**

**A**

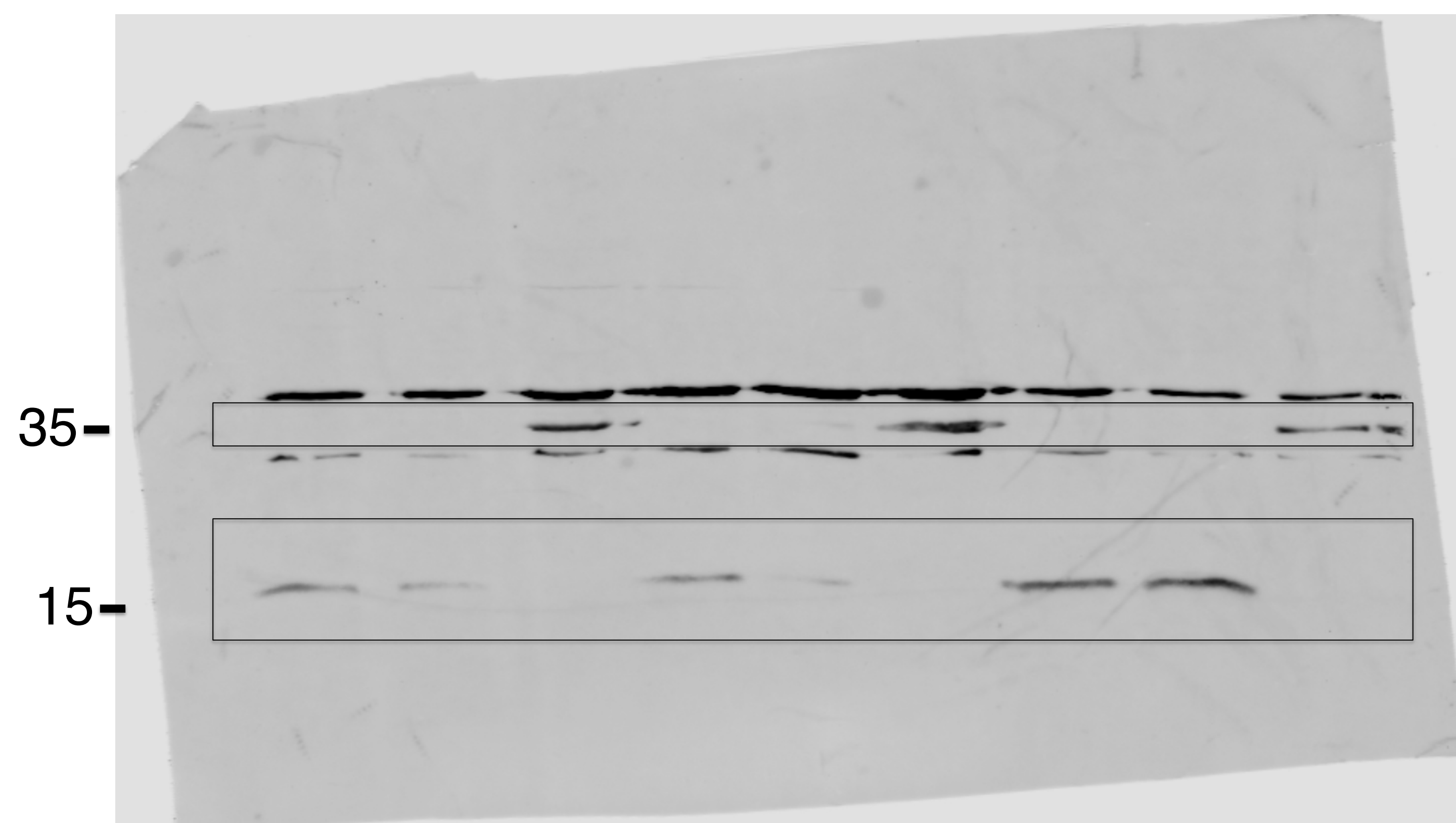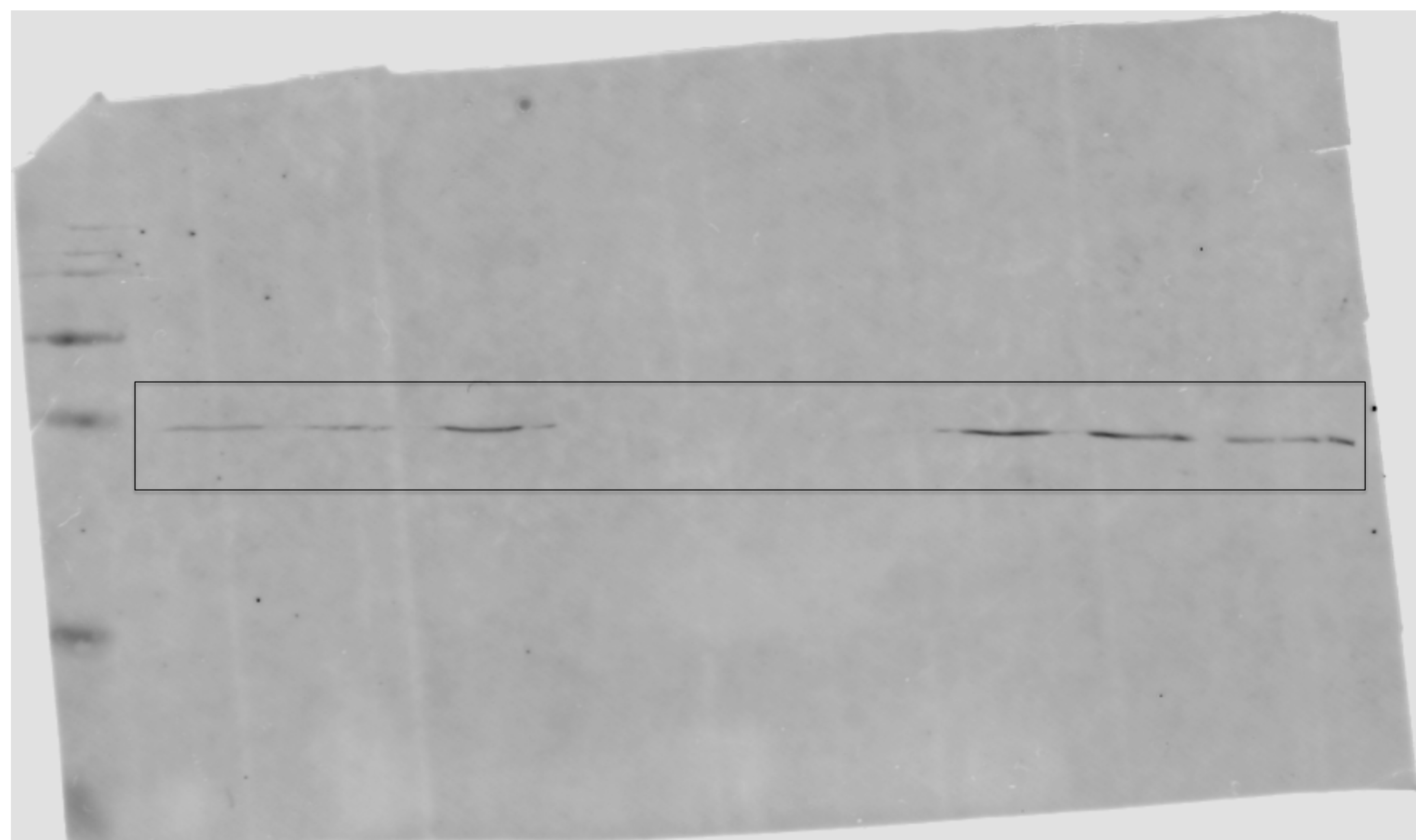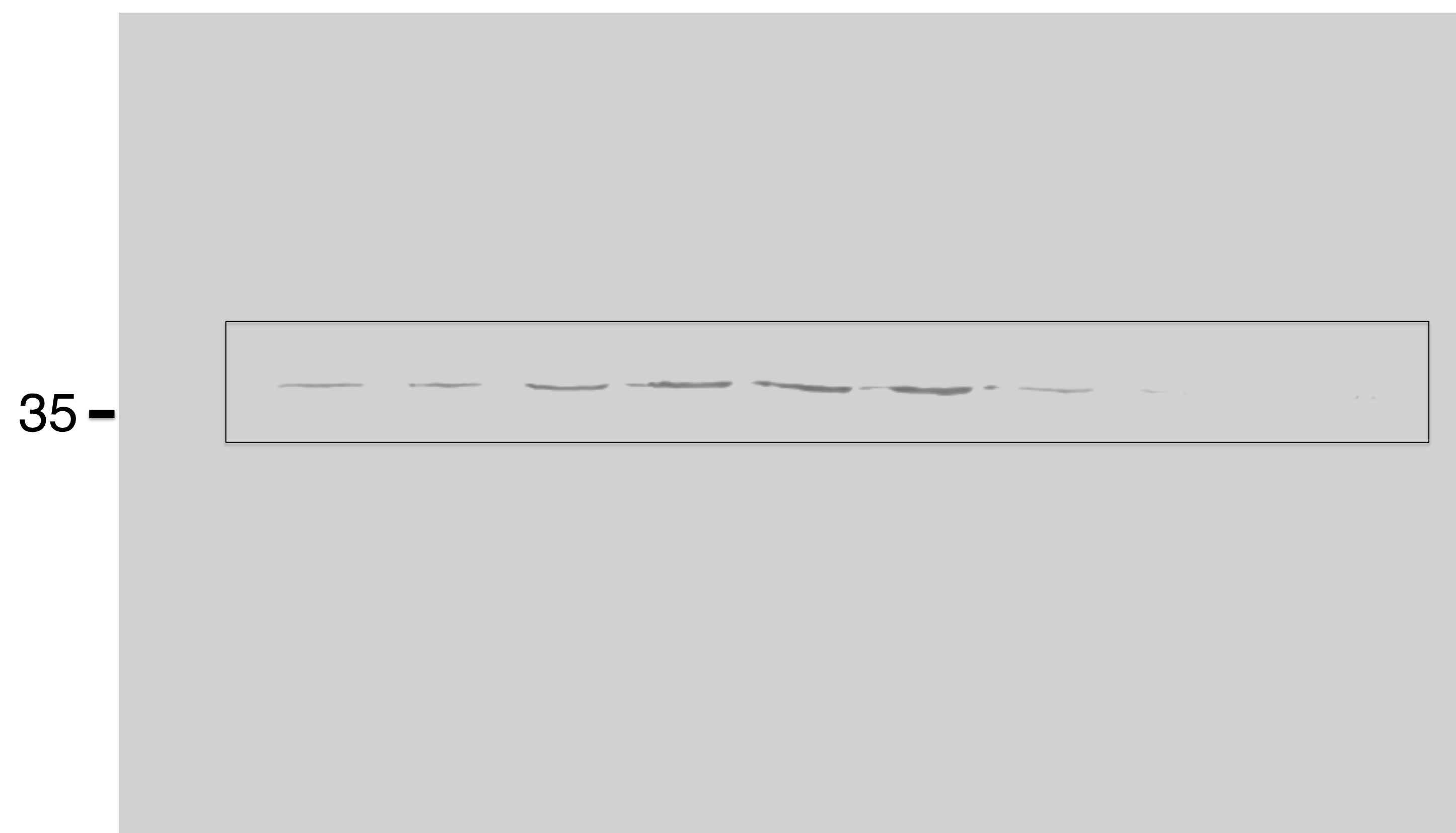

Supplement: Supplementary file 8 — Source Data for Figure 6 [file EMBR-23-e48754-s007.zip › Figure6_Source_Data/Raw Blots for EMBO Reports Figure 6.pdf]

**Figure 7**

**A**

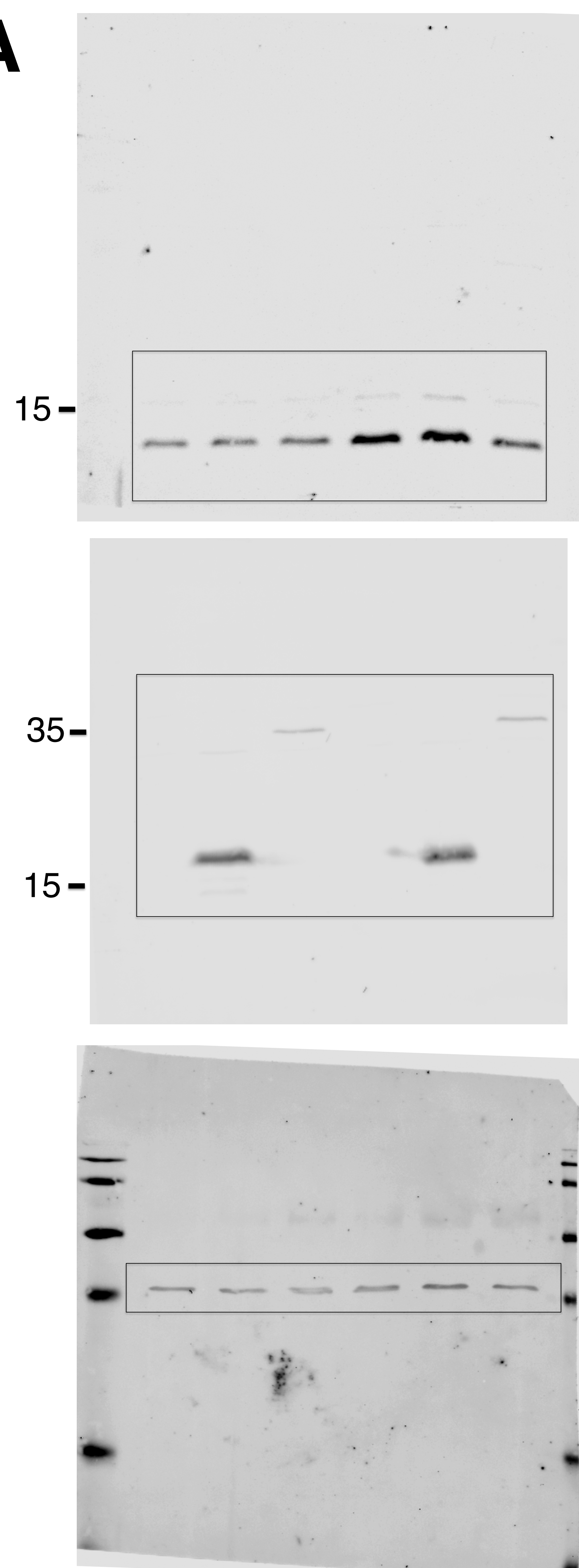

**B**

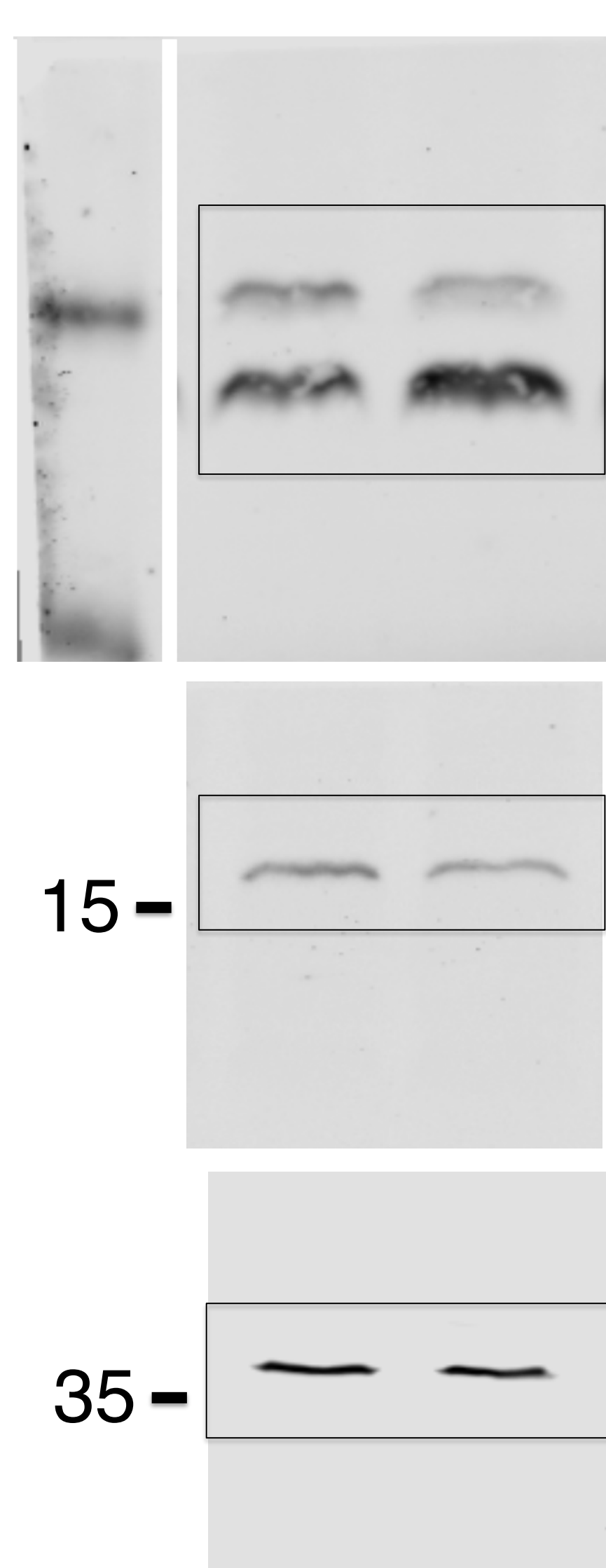

**D**

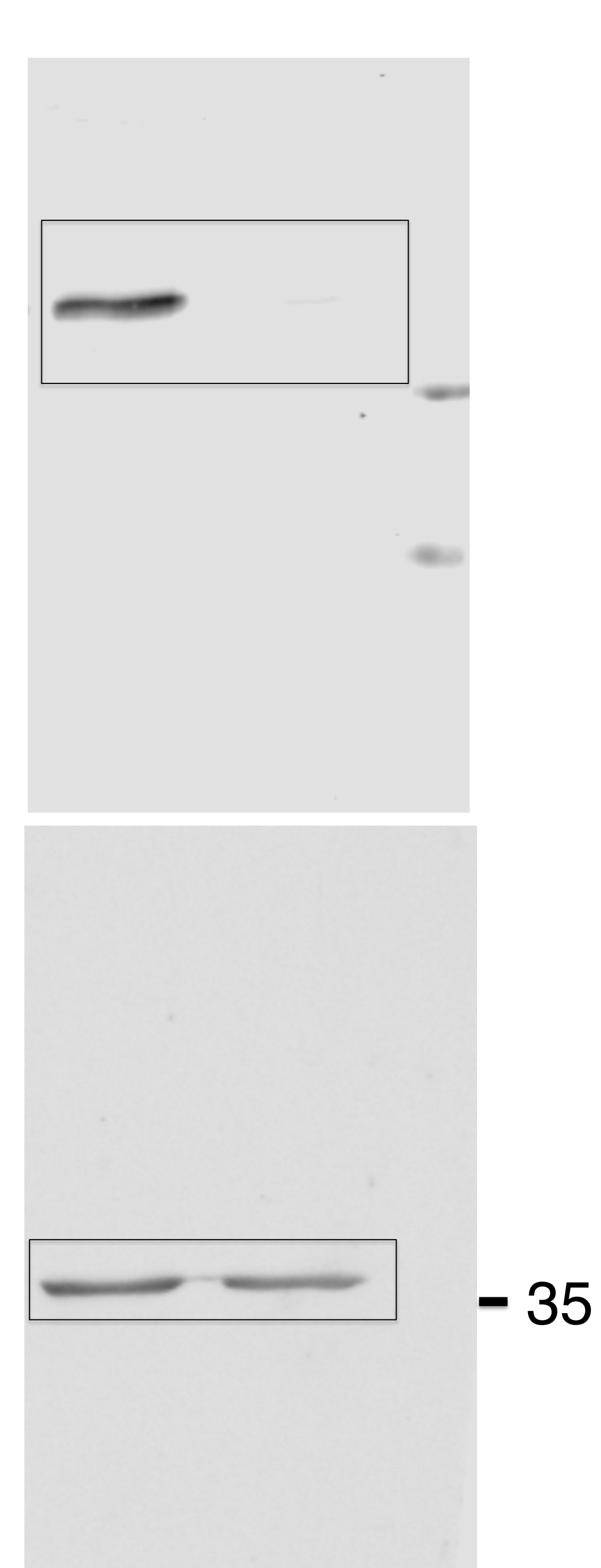

**C**

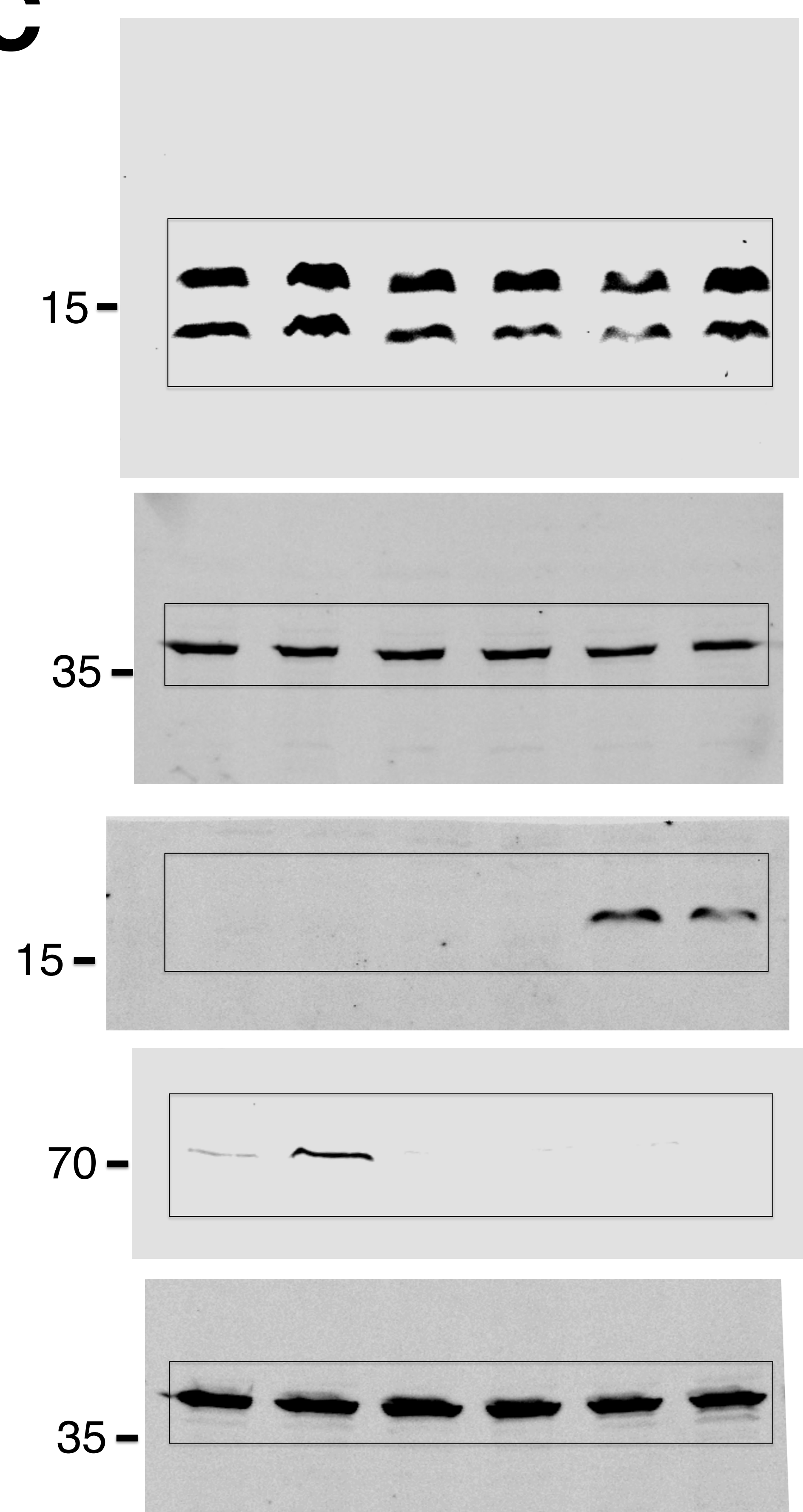

Supplement: Supplementary file 9 — Source Data for Figure 7 [file EMBR-23-e48754-s009.zip › Figure7_Source_Data/Raw Blots for EMBO Reports Figure 7.pdf]
