# Supplementary material for: The SUMO protease SENP3 regulates mitochondrial autophagy mediated by Fis1
Source: EMBO Rep. 2022 Jan 7;23(2):e48754. doi: 10.15252/embr.201948754 (PMC8811651; doi:10.15252/embr.201948754)
Supplement: Supplementary file 10 [file EMBR-23-e48754-s006.pdf]

EMBO reports

The SUMO Protease SENP3 regulates Mitochondrial Autophagy mediated by Fis1

Emily Waters, Kevin Wilkinson, Amy Harding, Ruth Carmichael, Darren Robinson, Helen Colley, and Chun Guo
DOI: 10.15252/embr.202052022

Corresponding author(s): Chun Guo (c.guo@sheffield.ac.uk)

Review Timeline:

Submission Date:	26th Jun 19
Editorial Decision:	1st Aug 19
Revision Received:	3rd Nov 20
Editorial Decision:	16th Feb 21
Revision Received:	14th Jul 21
Editorial Decision:	18th Oct 21
Revision Received:	31st Oct 21
Accepted:	24th Nov 21

Editor: Martina Rembold

Transaction Report:

(Note: With the exception of the correction of typographical or spelling errors that could be a source of ambiguity, letters and reports are not edited. Depending on transfer agreements, referee reports obtained elsewhere may or may not be included in this compilation. Referee reports are anonymous unless the Referee chooses to sign their reports.)

1st Editorial Decision — 1st Aug 2019

Dear Dr. Guo

Thank you for the submission of your research manuscript to EMBO reports. We have now received the two enclosed reports on it.

I am sorry to say, that the evaluation of your manuscript is not a positive one. As you will see, although both referees acknowledge that the findings are potentially interesting, they raise a number of largely overlapping criticisms of the manuscript and consider the data insufficient to support the main conclusions.

Given the nature of these concerns, the amount of work required to address them, the uncertain outcome of these experiments, and the fact that EMBO reports can only invite revision of papers that receive enthusiastic support from a majority of referees, I am sorry to say that we cannot offer to publish your manuscript.

I am sorry to disappoint you on this occasion, and hope that the referee comments will be helpful in your continued work in this area.

Yours sincerely

Martina Rembold, PhD
Editor
EMBO reports

****

Referee #1:

The reversible post-translational modification of proteins with the ubiquitin related SUMO modifier controls many central cell functions, including DNA damage response, gene expression or cell cycle progression. SUMO isopeptidases/proteases of the SENP family catalyze demodification of SUMO from target proteins. In this manuscript Waters and co-workers define a novel regulatory role of the SUMO isopeptidase SENP3 in mitochondrial autophagy (mitophagy). They propose that SENP3 is required for stress-induced mitophagy by the iron-chelator deferiprone. They further conclude that this is due to SENP3-mediated SUMO2/3 deconjugation from the mitochondrial fission protein Fis1, whose mitochondrial targeting is facilitated by desumoylation. While this is an interesting concept, I do feel that at this stage the data is very preliminary and not conclusive. As detailed below a series of control experiments and assay validation would be needed to support the proposed model.

1) The mitophagy assays rely on a Cherry/GFP-based pH-sensitive reporter system, termed MitopHfluorin. While these pH-sensitive reporters are becoming standard in the autophagy field, the authors generate a system, in which mitochondrial targeting is achieved through fusing the fluorescent proteins to the mitochondrial targeting sequence of the ActA protein of Listeria monocytogenes. Since this is a so far uncharacterized reporter system (with respect to mitochondrial targeting and autophagosomal/lysosomal degradation), the authors need to fully characterize and validate this system. Proper co-localization with respective marker proteins must be shown. The experiment provided in Supplementary Figure 1 is not sufficient and informative. Alternatively, the well-established and characterized mito-Keima reporter system could be used.
2) Along this line the datasets provided with the above mentioned reporter appear to be very variable and inconsistent. For example, in Figure 2B in the control (Nsi DFP) an average of 15 red puncta/cell are detected, while in Figure 3A only 6 puncta/cell are seen under the same conditions. This questions reproducibility of the data. More quantitative approaches, such as FACS analysis (e.g. with mitoKeima), are suggested.
3) The authors need to show whether SENP3 is also involved in Pink/parkin-mediated mitophagy or contributes to mitophagy induced by other stressors (paraquat etc.)
4) Generally the assays monitoring autophagy by LC3 lipidation need to be done in the presence and absence of bafilomycinA. Monitoring p62 levels is also highly recommended.
5) It remains to be demonstrated that knock-down of SENP3 induces SUMO2/3 conjugation to endogenous Fis1. So far all data rely on overexpression experiments (for example Figure 4D).
6) Only a single siRNA is used for depletion of SENP3. The phenotypes must be confirmed with at least one other siRNA directed against SENP3.
7) Knock-down complementation experiments with wild type and catalytic-dead SENP3 should be done to validate whether the catalytic activity of SENP3 is needed for its function in mitophagy.
8) In previous work the authors have shown that SENP3 acts as a SUMO2/3 deconjugase on Drp1. Even though the authors claim that Drp1 is not essential for Parkin-independent mitophagy, they need to validate this experimentally in their setup. Moreover, they should explore the contribution of Drp1 SUMOylation in this context.
9) The manuscript falls short in mechanistic data. How does deferiprone affect CHIP levels? How does SUMOylation of Fis1 determine mitochondrial targeting of Fis1? Does a linear fusion of SUMO2-Fis1 phenocopy the mitophagy defect?

10) The experiment shown in Suppl. Figure 2A is of low technical quality.

Referee #2:

The manuscript reports a novel role of the SUMO protease SENP3 and mitochondrial fission factor Fis1 in iron-chelator deferiprone (DFP) induced mitophagy. They report that DFP treatment induces downregulation of the E3 ubiquitin ligase CHIP, of which SENP3 is a substrate. SENP3 deSUMOylates Fis1 and, as a result, DFP induces Fis1 mitochondrial localization to induce mitophagy.

While the involvement of SENP3 and CHIP in DFP-induced mitophagy is potentially interesting, the authors fail to provide solid evidence to link Fis1 as the key inducer of mitophagy. Many figures are pretty thin without proper controls. Most importantly, no rescue data are provided for any of their siRNA experiments, which is a must. Some WB data are of poor quality and need to be repeated with better images.

Major points:
1. The authors show that Fis1 K149 is the residue sumoylated by SUMO-2/3. However, K149 resides at the very C-terminus after the transmembrane domain. Based on the Fis1 structure, the Fis1 C-tail is inside the mitochondrial inner membrane space. So how does SUMO-2/3 act on Fis1 tail in this location?
2. The authors claim that deSUMOylation of Fis1 by SENP3 stabilizes Fis1 on mitochondria. However, the fractionation data shown in Fig.4G is not convincing and improperly presented. There is no difference on Fis1 level in the lysate between WT Fis1 and Fis1 K149R, but there seems to be more Fis1 K149R in both the cytosol and mitochondria panels. It's surprising to see Fis1 in the cytosol. A proper presentation should have each fractionation run side by side on the same gel and probed with GAPDH and VDAC as the preparation control. Since it is transiently transfected, the data could be simply interpreted as K149R is expressed better.
3. The mitoPHfluorin reporter seems to be very robust in supplementary figure 1 (with DFP treatment, most mitochondria seem to be red) but less efficient in other figures such as 5C, 2B. Nonetheless, most red-only mitoPHfluorin spots appear to be still on mitochondria. Are all the mitochondria inside autolysosomes? A better reporter is mito-mKeima, which has been widely used and can be objectively quantified with FACS.
4. CRISPR/Cas9 has been widely used to make knockout cell lines, which has become routine in many labs. The authors should try to generate Fis1 KO and SENP3 KO cells to avoid off-target effects of siRNA and also boost the phenotypes for better study.
5. Iron chelation also leads to ferritinophagy. The authors should check if SENP3 or Fis1 knockdown affects DFP-induced ferritinophagy.
Minor points:
1. Fig.5B WB quality is unacceptable with the fuzzy bands. Same as supplementary figure 2A
2. In many figures, transfections were done in the absence or presence of DFP (24h) and then analyzed 48h or 72h post-transfection. DFP treatment should be done after transfection.
3. In the discussion, it is mentioned that "preventing SUMOylation promotes Fis1 mitochondrial localization". Is there any literature showing Fis1 is also localized in the cytosol?

** As a service to authors, EMBO Press provides authors with the ability to transfer a manuscript that one journal cannot offer to publish to another journal, without the author having to upload the manuscript data again. To transfer your manuscript to another EMBO Press journal using this service, please click on
Link Not Available

Referee Reports

Dear Dr. Guo

Thank you for the submission of your research manuscript to EMBO reports. We have now received the two enclosed reports on it.

I am sorry to say, that the evaluation of your manuscript is not a positive one. As you will see, although both referees acknowledge that the findings are potentially interesting, they raise a number of largely overlapping criticisms of the manuscript and consider the data insufficient to support the main conclusions.

Given the nature of these concerns, the amount of work required to address them, the uncertain outcome of these experiments, and the fact that EMBO reports can only invite revision of papers that receive enthusiastic support from a majority of referees, I am sorry to say that we cannot offer to publish your manuscript.

I am sorry to disappoint you on this occasion, and hope that the referee comments will be helpful in your continued work in this area.

Yours sincerely

Martina Rembold, PhD
Editor
EMBO reports

We thank the EMBO Reports reviewers for their interest, time and constructive and insightful critiques. By acting on all of their suggestions, we have carried out a substantial amount of additional experimental work and extensive revisions to address their concerns.

We have now addressed each of the concerns raised by the reviewers, and respond to their queries in a point-by-point response below. We feel strongly that the new manuscript contains more compelling data to support the main conclusions. Overall we feel it is significantly improved and we very much hope it will be acceptable for publication in ***EMBO Reports***.

Referee #1:

The reversible post-translational modification of proteins with the ubiquitin related SUMO modifier controls many central cell functions, including DNA damage response, gene expression or cell cycle progression. SUMO isopeptidases/proteases of the SENP family catalyze demodification of SUMO from target proteins. In this manuscript Waters and co-workers define a novel regulatory role of the SUMO isopeptidase SENP3 in mitochondrial autophagy (mitophagy). They propose that SENP3 is required for stress-induced mitophagy by the iron-chelator deferiprone. They further conclude that this is due to SENP3-mediated SUMO2/3 deconjugation from the mitochondrial fission protein Fis1, whose mitochondrial targeting is facilitated by desumoylation. While this is an interesting concept, I do feel that at this stage the data is very preliminary and not conclusive. As detailed below a series of control experiments and assay validation would be needed to support the proposed model.

We thank the reviewer for their interest in the novel role for SENP3 in mitophagy demonstrated in our work. We greatly appreciate her/his helpful comments on the data presented in the last version of our manuscript.

1) The mitophagy assays rely on a Cherry/GFP-based pH-sensitive reporter system, termed MitopHfluorin. While these pH-sensitive reporters are becoming standard in the autophagy field, the authors generate a system, in which mitochondrial targeting is achieved through fusing the fluorescent proteins to the mitochondrial targeting sequence of the ActA protein of Listeria monocytogenes. Since this is a so far uncharacterized reporter system (with respect to mitochondrial targeting and autophagosomal/lysosomal degradation), the authors need to fully characterize and validate this system. Proper co-localization with respective marker proteins must be shown. The experiment provided in Supplementary Figure 1 is not sufficient and informative. Alternatively, the well-established and characterized mito-Keima reporter system could be used.

We thank the reviewer for this constructive advice. As requested, we have conducted further characterisation experiments to address the issues raised. Our new results are included in the revised manuscript:

i) We now confirm mitochondrial localisation of mito-pHfluorin by demonstrating colocalisation with the mitochondrial marker COX IV (see Supplementary Figures 2B and 2C);
ii) We confirm that mitochondria-containing lysosomes are detected using mito-pHfluorin in DFP-treated HeLa cells by colocalisation of SEP-quenched mito-pHfluorin with LysoTracker dye (see Supplementary Figure 3B);
iii) We establish criteria for quantifying mitophagic red punta using mito-pHfluorin in DFP-treated HeLa cells (see Supplementary Figure 4);
iv) As suggested, we now confirm our findings using the well-characterised mito-Keima reporter system. While this system confirms induction of mitophagy by DFP in live cells (see Figures 3C and 3D and Supplementary Table 1), our new data indicate that mito-pHfluorin, but not mito-Keima, is suitable for the detection of DFP-induced mitophagy in HeLa cells fixed with 4% PFA (see Supplementary Figure 7).

2) Along this line the datasets provided with the above mentioned reporter appear to be very variable and inconsistent. For example, in Figure 2B in the control (Nsi DFP) an average of 15 red puncta/cell are detected, while in Figure 3A only 6 puncta/cell are seen under the same conditions. This questions reproducibility of the data. More quantitative approaches, such as FACS analysis (e.g. with mitoKeima), are suggested.

We thank the reviewer for raising this important point. Based on our quantifying criteria for DFP-induced mitophagy (see Supplementary Figure 4), we have now conducted further experiments to address this issue. The updated figures referred to by the reviewer are now shown in Figure 4C.

In fixed HeLa cells transfected with non-specific siRNA (Nsi) and treated with DFP approximately 10-15 red puncta/cell were detected using mito-pHfluorin. To supplement these findings, as suggested, we have established a HeLa cell line stably expressing mito-Keima through G418 (Geneticin) selection followed by FACS sorting (see Figure 3C). To the best of our knowledge, so far no publication has reported using mito-Keima-based FACS for detecting DFP-induced mitophagy, which is generally accepted to be milder than CCCP-induced Parkin-dependent mitophagy (Sargsyan et al., 2015). Potentially due to this issue, we were unable to detect the shift of Keima green to Keima red in DFP-treated HeLa cells using FACS. However, in agreement with our findings with mito-pHfluorin, we were able to quantify DFP-induced mitophagy in living HeLa cells with stable Keima expression under different conditions. Following DFP treatment (1mM; 24 h), approximately 36% of cells became mitophagic (as determined by a shift of mito-Keima from green to red), and this was prevented by either SENP3 knockdown or autophagy inhibition using chloroquine (50μM; 24 h) (see Figures 3C and 3D and Supplementary Table 1).

3) The authors need to show whether SENP3 is also involved in Pink/parkin-mediated mitophagy or contributes to mitophagy induced by other stressors (paraquat etc.)

We thank the reviewer for raising this interesting point. Although in the present study our efforts have been focused on elucidating the role of SENP3-mediated deSUMOylation in DFP-induced mitophagy, which is generally regarded as Parkin-independent, we have now examined the functional consequences of SENP3 loss on mitophagic autophagsome formation (LC3-II induction) by CCCP, which is one of the most well-characterised

inducers of Pink/Parkin-dependent mitophagy (Narendra et al., 2008) or paraquat, which is capable of inducing both macroautophagy and mitophagy (Georgakopoulos et al., 2017, González-Polo et al., 2007). While treatment of cells with either inducer resulted in elevated SENP3 levels, RNAi-mediated SENP3 knockdown did not affect CCCP-induced LC3-II induction in HEK293 cells (Supplementary Figure 13), suggesting that SENP3 is not required for Pink/Parkin-dependent mitophagy. Interestingly, consistent with a recent study showing an inhibitory role for SENP3 in macroautophagy (Liu et al., 2019), RNAi-mediated SENP3 knockdown increased basal LC3-II levels, and prevented a further increase in LC3-II induction by paraquat in SH-SY5Y cells (see Supplementary Figure 14), suggesting a potential role for SENP3 in paraquat-induced autophagy (see text in the Discussion of the revised manuscript, under the title 'Role of SENP3 in autophagy regulation'). Future work is needed to further clarify the precise role for SENP3 in macroautophagy and mitophagy induced by various stressors.

4) Generally the assays monitoring autophagy by LC3 lipidation need to be done in the presence and absence of bafilomycinA. Monitoring p62 levels is also highly recommended.

We thank the reviewer for raising these two important points.

At the beginning of this project we first asked if DFP-induced LC3-II induction is due to enhanced autophagosome formation. Consistent with the original report of DFP-induced mitophagy, where treatment of cells with the autophagy inhibitor bafilomycinA increased LC3-II levels in both control and DFP-treated cells (Allen et al., 2013), in our study addition of chloroquine (another widely used autophagy inhibitor) to control or DFP-treated cells increased LC3-II visualisation, indicating upregulation of autophagosome formation upon DFP treatment (see Supplementary Figure 1). This has been further confirmed in the experiments concerning LC3-II levels in the absence or presence of chloroquine (see Supplementary Figures 5 and 8). For better LC3-II detection, some experiments concerning the changes in LC3-II levels were performed in the presence of chloroquine (see Figures 3A, 4A, 4B, 7A, 7B and 7C).

As suggested, we have now examined p62 levels in control and DFP-treated HeLa cells. In our hands treatment of cells with DFP resulted in a decrease in p62 levels (see Supplementary Figure 6). This is in contrast with the finding from a previous study (Allen et al., 2013), but consistent with that from two other studies (Sargsyan et al., 2015, Yamashita et al., 2016), supporting a potential role for p62 in DFP-induced mitophagy.

5) It remains to be demonstrated that knock-down of SENP3 induces SUMO2/3 conjugation to endogenous Fis1. So far all data rely on overexpression experiments (for example Figure 4D).

We thank the reviewer for providing this constructive advice. In the revised manuscript, in addition to the results shown in Figure 4D of the original manuscript (now Figure 5C), we have provided evidence showing increased endogenous Fis1 SUMO-2/3-lyation upon RNAi-mediated SENP3 knockdown in HeLa cells (see Figure 5D).

6) Only a single siRNA is used for depletion of SENP3. The phenotypes must be confirmed with at least one other siRNA directed against SENP3.

We thank the reviewer for raising this very important point. We have now performed additional experiments using an alternative siRNA duplex for targeting *SENP3* (target sequence AACGUGGACAUCUUCAAUA) to address the issues raised. Our new results (see Figures 3B and 3D and Supplementary Figures 5B and 5C) have confirmed our finding that SENP3 is essential for DFP-induced mitophagy.

7) Knock-down complementation experiments with wild type and catalytic-dead SENP3 should be done to validate whether the catalytic activity of SENP3 is needed for its function in mitophagy.

This is a very important point to be addressed. Accordingly, we have carried out additional experiments where we have knocked down endogenous SENP3 and replaced it with either WT or a catalytically inactive mutant. Importantly, our new results show that the catalytic activity of SENP3 is essential for its function in mitophagy regulation and exclude the possibility that an off-target effect of SENP3 knockdown results in the abolished LC3-II induction by DFP (see Figure 3B and Supplementary Figure 5C).

8) In previous work the authors have shown that SENP3 acts as a SUMO2/3 deconjugase on Drp1. Even though the authors claim that Drp1 is not essential for Parkin-independent mitophagy, they need to validate this experimentally in their setup. Moreover, they should explore the contribution of Drp1 SUMOylation in this context.

This is an interesting point. Whether Drp1 is essential for Parkin-independent mitophagy is a fundamental broad question, since there are various types of mitophagy that do not seem to require Parkin involvement (Villa et al., 2018). We therefore feel that answering this question more generally is beyond the scope of this manuscript. Nonetheless, we have now examined the contribution of Drp1 SUMOylation to DFP-induced mitophagy, which is the primary focus of this manuscript. Our results indicate that Drp1 SUMOylation is not required for DFP-induced mitophagy, since replacement of endogenous Drp1 with either RNAi-resistant YFP-Drp1^{R} wild-type or non-SUMOylatable YFP-Drp1^{R} 4KR mutant does not alter the extent of LC3-II induction by DFP (see Supplementary Figure 8).

9) The manuscript falls short in mechanistic data. How does deferiprone affect CHIP levels? How does SUMOylation of Fis1 determine mitochondrial targeting of Fis1? Does a linear fusion of SUMO2-Fis1 phenocopy the mitophagy defect?

These questions are very insightful, and we have performed additional experiments to address them. However, we contend that our data demonstrating the role of SENP3-mediated Fis1 deSUMOylation in DFP-induced mitophagy, which is the major focus of our manuscript, is mechanistic in nature.

Nonetheless, to directly address the reviewer's specific questions, we have now shown by quantitative (q) PCR that DFP decreases *CHIP* mRNA levels in HeLa cells, indicating that the DFP-induced decrease in levels of CHIP are most likely due to downregulation of *CHIP* gene expression (see Figure 2B). Since DFP treatment also results in decreased *SENP3* mRNA levels (see Figure 1E) the increased SENP3 protein level is most likely due to increased SENP3 stability (see Figures 1F and 1G). Thus, in this respect, we believe we have now provided additional mechanistic data regarding the altered levels of CHIP and SENP3 in response to DFP.

While we have been unable to fully elucidate the mechanism of how SUMOylation regulates mitochondrial targeting of Fis1, nevertheless, our results from subcellular fractionation and imaging experiments using Flag-Fis1 wild-type, Flag-Fis1 K149R SUMOylation deficient mutant or, as suggested by the reviewer, a Flag-Fis1-SUMO-2$^{\Delta GG}$ fusion protein suggest that SUMOylation decreases Fis1 mitochondrial localization, perhaps via increasing Fis1 distribution in the endoplasmic reticulum (see Figure 6). Finally, as expected, expression of a Flag-Fis1-SUMO-2$^{\Delta GG}$ fusion, but not Flag-Fis1 WT, prevents DFP-mediated induction of LC3-II, supporting the importance of Fis1 deSUMOylation in this form of mitophagy (see Figures 7A).

10) The experiment shown in Suppl. Figure 2A is of low technical quality.

We thank the reviewer for pointing out this. This Figure has been updated with a better example (now Supplementary Figure 10A).

Referee #2:

The manuscript reports a novel role of the SUMO protease SENP3 and mitochondrial fission factor Fis1 in iron-chelator deferiprone (DFP) induced mitophagy. They report that DFP treatment induces downregulation of the E3 ubiquitin ligase CHIP, of which SENP3 is a substrate. SENP3 deSUMOylates Fis1 and, as a result, DFP induces Fis1 mitochondrial localization to induce mitophagy.

We thank the reviewer for this clear, concise and accurate précis of the original manuscript submitted to EMBO Reports.

While the involvement of SENP3 and CHIP in DFP-induced mitophagy is potentially interesting, the authors fail to provide solid evidence to link Fis1 as the key inducer of mitophagy. Many figures are pretty thin without proper controls. Most importantly, no rescue data are provided for any of their siRNA experiments, which is a must. Some WB data are of poor quality and need to be repeated with better images.

We greatly appreciate these helpful comments on the data presented in the last version of our manuscript. We have now provided rescue/knockout data for the major findings of the manuscript and replaced some of the representative Western blots with better examples. Through our revision work, we believe that the manuscript is much improved.

Major points:

1. The authors show that Fis1 K149 is the residue sumoylated by SUMO-2/3. However, K149 resides at the very C-terminus after the transmembrane domain. Based on the Fis1 structure, the Fis1 C-tail is inside the mitochondrial inner membrane space. So how does SUMO-2/3 act on Fis1 tail in this location?

We thank the reviewer for this important and interesting question. As the reviewer notes, when Fis1 is mitochondrially located, K149 lies inside the mitochondrial inner membrane space, raising the question of how the cytosolic SUMOylation machinery can act upon it in this location. However, our data suggest that SUMOylation acts as a mobilisation factor that controls the extent of Fis1 delivery to mitochondria. Thus, we hypothesise that SUMOylation acts on Fis1 not at the mitochondrial inner membrane, but instead targets the non-mitochondrial population, reducing its mitochondrial delivery. Indeed, our data support a model whereby *deSUMOylation* of Fis1 promotes its mitochondrial targeting.

Recent studies have revealed that in addition to mitochondria, two organelles, peroxisomes and endoplasmic reticulum (ER), harbor subpopulations of Fis1, which form a non-mitochondrial pool of Fis1 in cultured mammalian cells (Ji et al., 2017, Stojanovski et al., 2004). The C-terminal tail of Fis1 is important for the targeting of Fis1 to the mitochondria since without the C-terminal tail Fis1 fails to be localized to the mitochondrial outer membrane (Rojansky et al., 2016). We reasoned that SUMOylation at the C-terminus of Fis1 may inhibit mitochondrial targeting and, as a potential mechanism, deSUMOylation of Fis1 would allow targeting of the non-mitochondrial pool of Fis1 to the mitochondria to initiate mitophagy. Importantly, the non-SUMOylatable K149R Fis1 mutant was more strongly associated with the mitochondrial fraction. However, the levels of Fis1 WT and Fis1 K149R in the cytosolic fraction, which likely contains Fis1-associated peroxisomes, are comparable (see Figure 6A), implying that deSUMOylation does not affect Fis1 targeting to the peroxisomes. Considering that perinuclear ER continuous with the nucleus could be a 'contaminant' component in the nuclear fraction in the traditional hypotonic buffer-based preparation used here (Huber et al., 2003, Shaiken & Opekun, 2014), we performed immunocytochemistry to examine ER distributions of exogenously expressed Fis1. A Fis1-SUMO-2$^{\Delta GG}$ fusion seemed to be more co-localised with ER than did Fis1 WT, and SUMOylation-deficient Fis1 K149R did not seem to co-localise with ER (see Figure 6B), suggesting that Fis1 in its SUMOylated state is more likely to be localized to areas of the cell in which ER is located. Together, these results suggest that the SUMOylation status of Fis1 may serve as a key molecular switch to regulate its mitochondrial localization.

2. The authors claim that deSUMOylation of Fis1 by SENP3 stabilizes Fis1 on mitochondria. However, the fractionation data shown in Fig.4G is not convincing and improperly presented. There is no difference on Fis1 level in the lysate between WT Fis1 and Fis1 K149R, but there seems to be more Fis1 K149R in both the cytosol and mitochondria panels. It's surprising to see Fis1 in the cytosol. A proper presentation should have each fractionation run side by side on the same gel and probed with GAPDH and VDAC as the preparation control. Since it is transiently transfected, the data could be simply interpreted as K149R is expressed better.

The original Figure 4G has been replaced with a new figure (see Figure 6A) as discussed above. In fact, when same amount of DNA is transfected, the Fis1 K149R mutant is not expressed as well as its wild-type counterpart, potentially due to increased mitophagic lysosomal degradation.

3. The mitopHfluorin reporter seems to be very robust in supplementary figure 1 (with DFP treatment, most mitochondria seem to be red) but less efficient in other figures such as 5C, 2B. Nonetheless, most red-only mitoPHfluorin spots appear to be still on mitochondria. Are all the mitochondria inside autolysosomes? A better reporter is mito-mKeima, which has been widely used and can be objectively quantified with FACS.

First of all, we greatly appreciate the reviewer's comment on the potential difference in the usage of our mito-pHfluorin probe under different conditions. The image showing the detection of DFP-induced mitophagy with mito-pHfluorin in Supplementary Figure 1 of the previous version of the manuscript was taken using an

inverted fluorescence microscope (Axiovert 200 M; Carl Zeiss Microimaging, Inc.) whilst the other images, including the ones shown in Figures 2B and 5C in the previous version, were taken using a confocal microscope (Zeiss LSM 880 Airyscan). For consistency, the image previously in Supplementary Figure 1 has been removed.

Nonetheless, to supplement our findings we have established quantifying criteria for using the mito-pHfluorin reporter to detect DFP-induced mitophagy (see Supplementary Figure 4), and we now use this methodology throughout the manuscript. Our results indicate that in fixed HeLa cells transfected with non-specific siRNA (Nsi) and treated with DFP, approximately 10-15 red puncta per cell were detected using mito-pHfluorin (see Figures 3E and 4C). Furthermore, using LysoTracker staining of DFP-treated HeLa cells we have confirmed that mito-pHfluorin is suitable for detecting mitochondria-containing autolysosomes (see Supplementary Figure 3B).

We thank the reviewer, like reviewer 1, for their suggestion that we supplement our findings by using the widely-characterised mito-Keima reporter. To the best of our knowledge, so far no publication has reported using mito-Keima-based FACS for detecting DFP-induced mitophagy, which seems to be less dramatic than CCCP-induced Parkin-dependent mitophagy (Sargsyan et al., 2015). Potentially for this reason, we were unable to detect the shift of Keima green to Keima red in DFP-treated HeLa cells using FACS. Nonetheless, to quantify DFP-induced mitophagy directly we counted living HeLa cells stably expressing mito-Keima under different conditions. Consistent with our mito-pHfluorin findings, our results show that following DFP treatment (1mM; 24 h) approximately 36% of cells became mitophagic, and both SENP3 knockdown, or treatment of cells with the autophagy inhibitor chloroquine (50 μM; 24 h), significantly reduced the extent of DFP-induced mitophagy (see Figures 3C and 3D and Supplementary table 1).

In addition to this, and consistent with previous studies (Sun et al., 2017), we found that mito-Keima does not seem to be suitable for detecting mitophagy in fixed cells (see Supplementary Figure 7). In this study our work has been focused on determining the specific roles of both SENP3 and Fis1 in DFP-induced mitophagy; therefore, immunocytochemistry is essential for pinpointing the localization of SENP3/Fis1 whilst monitoring mitophagy in cells fixed with PFA, conditions under which the mito-pHfluorin appears to be superior to mito-Keima.

4. CRISPR/Cas9 has been widely used to make knockout cell lines, which has become routine in many labs. The authors should try to generate Fis1 KO and SENP3 KO cells to avoid off-target effects of siRNA and also boost the phenotypes for better study.

We greatly appreciate the reviewer's concern regarding the potential advantages of using CRISPR/Cas9-mediated knockout (KO) of Fis1 or SENP3 for this study.

Accordingly, we have created Fis1 KO HeLa cells using this advanced technique. As expected, similar to our observation using Fis1 knockdown HeLa cells, DFP failed to induce an increase in LC3-II in cells lacking Fis1 (see Figure 4B), substantiating the importance of Fis1 in DFP-induced mitophagy.

Our effort to generate SENP3 KO HeLa cells using CRISPR/Cas9 has been hampered by a cell viability issue, which is most likely to be due to significant DNA damage caused by genetic ablation of this SUMO protease (Xu et al., 2019). Alternatively, we have carried out additional experiments using a new independent siRNA duplex (SENP3i (II)) to knockdown SENP3, and our results reconfirm that SENP3 is required for DFP-induced mitophagy (see Figures 3B and 3D and Supplementary Figures 5B and 5C). Furthermore, our knockdown plus replacement experiments indicate that SENP3 deSUMOlyating activity is essential for the DFP-induced increase in LC3-II levels, minimising the possibility of artefactual off-target effects of the SENP3 siRNAs used (see Figure 3B and Supplementary Figure 5C).

5. Iron chelation also leads to ferritinophagy. The authors should check if SENP3 or Fis1 knockdown affects DFP-induced ferritinophagy.

We greatly appreciate this important point raised by the reviewer. Current evidence indicates that treatment of mammalian cells with the iron chelators deferoxamine, deferasirox or DFP leads to ferritin loss and, among the three drugs, only deferoxamine has proved to be capable of inducing ferritinophagy (De Domenico et al., 2009). However, we have now performed further experiments to test this directly, and show that RNAi-mediated

SENP3 depletion does not affect the DFP-induced loss of ferritin heavy chain 1 (FTH1), suggesting SENP3 is not involved in DFP-induced ferritinophagy (see Supplementary Figure 12).

Minor points:
1. Fig.5B WB quality is unacceptable with the fuzzy bands. Same as supplementary figure 2A

Figure 5B (now Figure 7C) and supplementary Figure 2A (now Supplementary Figure 10A) have been updated.

2. In many figures, transfections were done in the absence or presence of DFP (24h) and then analyzed 48h or 72h post-transfection. DFP treatment should be done after transfection.

We apologise if we were not clear on this point in our original manuscript. All DFP treatments were done 2 days post-transfection, as described in the Figure legends.

3. In the discussion, it is mentioned that "preventing SUMOylation promotes Fis1 mitochondrial localization". Is there any literature showing Fis1 is also localized in the cytosol?

As discussed above, three subpopulations of Fis1 are associated with mitochondria, peroxisomes and ER in cultured mammalian cells (Ji et al., 2017, Stojanovski et al., 2004). The cytosolic fractions prepared here likely contain Fis1 associated with peroxisomes which, along with the ER, constitute the 'non-mitochondrial' pool of Fis1. Our data demonstrate the non-SUMOylatable K149R Fis1 mutant was more strongly associated with the mitochondrial fraction. However, the levels of Fis1 WT and Fis1 K149R in the cytosolic fraction are comparable (see the histogram of Figure 6A), implying that deSUMOylation does not affect Fis1 targeting to peroxisomes. Building on these findings we performed imaging of a Fis1-SUMO-$2^{\Delta GG}$ fusion, which displayed greater colocalization with ER than did Fis1 WT, while SUMOylation-deficient Fis1 K149R did not seem to co-localise with ER (see Figure 6B). Together, these results suggest that the SUMOylation status of Fis1 may serve as a key molecular switch to regulate the balance between the mitochondrial and non-mitochondrial pool of Fis1, with deSUMOylation of Fis1 favouring mitochondrial localization.

Reference

Allen GF, Toth R, James J, Ganley IG (2013) Loss of iron triggers PINK1/Parkin-independent mitophagy. EMBO Rep 14: 1127-35

De Domenico I, Ward DM, Kaplan J (2009) Specific iron chelators determine the route of ferritin degradation. Blood 114: 4546-51

Georgakopoulos ND, Wells G, Campanella M (2017) The pharmacological regulation of cellular mitophagy. Nature chemical biology 13: 136-146

González-Polo RA, Niso-Santano M, Ortíz-Ortíz MA, Gómez-Martín A, Morán JM, García-Rubio L, Francisco-Morcillo J, Zaragoza C, Soler G, Fuentes JM (2007) Inhibition of paraquat-induced autophagy accelerates the apoptotic cell death in neuroblastoma SH-SY5Y cells. Toxicological sciences : an official journal of the Society of Toxicology 97: 448-58

Huber LA, Pfaller K, Vietor I (2003) Organelle proteomics: implications for subcellular fractionation in proteomics. Circulation research 92: 962-8

Ji WK, Chakrabarti R, Fan X, Schoenfeld L, Strack S, Higgs HN (2017) Receptor-mediated Drp1 oligomerization on endoplasmic reticulum. J Cell Biol 216: 4123-4139

Liu K, Guo C, Lao Y, Yang J, Chen F, Zhao Y, Yang Y, Yang J, Yi J (2019) A fine-tuning mechanism underlying self-control for autophagy: deSUMOylation of BECN1 by SENP3. Autophagy: 1-16

Narendra D, Tanaka A, Suen DF, Youle RJ (2008) Parkin is recruited selectively to impaired mitochondria and promotes their autophagy. J Cell Biol 183: 795-803

Rojansky R, Cha MY, Chan DC (2016) Elimination of paternal mitochondria in mouse embryos occurs through autophagic degradation dependent on PARKIN and MUL1. eLife 5

Sargsyan A, Cai J, Fandino LB, Labasky ME, Forostyan T, Colosimo LK, Thompson SJ, Graham TE (2015) Rapid parallel measurements of macroautophagy and mitophagy in mammalian cells using a single fluorescent biosensor. Scientific reports 5: 12397

Shaiken TE, Opekun AR (2014) Dissecting the cell to nucleus, perinucleus and cytosol. Scientific reports 4: 4923

Stojanovski D, Koutsopoulos OS, Okamoto K, Ryan MT (2004) Levels of human Fis1 at the mitochondrial outer membrane regulate mitochondrial morphology. J Cell Sci 117: 1201-10

Sun N, Malide D, Liu J, Rovira, II, Combs CA, Finkel T (2017) A fluorescence-based imaging method to measure in vitro and in vivo mitophagy using mt-Keima. Nature protocols 12: 1576-1587

Villa E, Marchetti S, Ricci JE (2018) No Parkin Zone: Mitophagy without Parkin. Trends Cell Biol 28: 882-895

Xu R, Yu S, Zhu D, Huang X, Xu Y, Lao Y, Tian Y, Zhang J, Tang Z, Zhang Z, Yi J, Zhu HH, Zheng X (2019) hCINAP regulates the DNA-damage response and mediates the resistance of acute myelocytic leukemia cells to therapy. Nature communications 10: 3812

Yamashita SI, Jin X, Furukawa K, Hamasaki M, Nezu A, Otera H, Saigusa T, Yoshimori T, Sakai Y, Mihara K, Kanki T (2016) Mitochondrial division occurs concurrently with autophagosome formation but independently of Drp1 during mitophagy. J Cell Biol 215: 649-665

1st Revision - Editorial Decision 16th Feb 2021

Dear Dr. Guo

Thank you for the submission of your research manuscript to our journal. Please accept my apologies for the unusual delay in handling your manuscript. I have asked the same reviewers, who had evaluated your manuscript when it was first submitted to also assess the resubmitted version. Please find their reports copied below.

As you will see, both referees acknowledge that the study has been significantly improved but they also point out a number of concerns that need to be addressed to substantiate the data. Some of the data obtained using the mito-Keima system seem to be missing and need to be provided. Co-localization of mito-pHfluorin with a marker of the MOM would further substantiate this reporter. Rescue experiments for the Fis1 KO cells need to be provided and the effect of SUMOylation of just a fraction of Fis1 needs to be explained.

Given these constructive comments, we would like to invite you to address these few remaining concerns. Please address all referee concerns (as detailed above and in their reports) and take their suggestions taken on board. Please address all referee concerns in a complete point-by-point response. Acceptance of the manuscript will depend on a positive outcome of a second round of review. It is EMBO reports policy to allow a single round of revision only and acceptance or rejection of the manuscript will therefore depend on the completeness of your responses included in the next, final version of the manuscript.

We invite you to submit your manuscript within three months of a request for revision. This would be May 16th in your case. However, we are aware of the fact that many laboratories are not fully functional due to COVID-19 related shutdowns and we have therefore extended the revision time for all research manuscripts under our scooping protection to allow for the extra time required to address essential experimental issues. Please contact us to discuss the time needed and the revisions further.

****
IMPORTANT NOTE: we perform an initial quality control of all revised manuscripts before re-review. Your manuscript will FAIL this control and the handling will be DELAYED if the following APPLIES:

1) A data availability section is missing.
2) Your manuscript contains error bars based on n=2. Please use scatter blots showing the individual datapoints in these cases. The use of statistical tests needs to be justified.
****

When submitting your revised manuscript, please carefully review the instructions that follow below. Failure to include requested items will delay the evaluation of your revision.

When submitting your revised manuscript, we will require:

1) a .docx formatted version of the manuscript text (including legends for main figures, EV figures and tables). Please make sure that the changes are highlighted to be clearly visible.

2) individual production quality figure files as .eps, .tif, .jpg (one file per figure).
Please download our Figure Preparation Guidelines (figure preparation pdf) from our Author Guidelines pages https://www.embopress.org/page/journal/14693178/authorguide for more info on how to prepare your figures.

3) a .docx formatted letter INCLUDING the reviewers' reports and your detailed point-by-point responses to their comments. As part of the EMBO Press transparent editorial process, the point-by-point response is part of the Review Process File (RPF), which will be published alongside your paper.

4) a complete author checklist, which you can download from our author guidelines (<https://www.embopress.org/page/journal/14693178/authorguide>). Please insert information in the checklist that is also reflected in the manuscript. The completed author checklist will also be part of the RPF.

5) Please note that all corresponding authors are required to supply an ORCID ID for their name upon submission of a revised manuscript (<https://orcid.org/>). Please find instructions on how to link your ORCID ID to your account in our manuscript tracking system in our Author guidelines
(<https://www.embopress.org/page/journal/14693178/authorguide#authorshipguidelines>)

6) We replaced Supplementary Information with Expanded View (EV) Figures and Tables that are collapsible/expandable online. A maximum of 5 EV Figures can be typeset. EV Figures should be cited as 'Figure EV1, Figure EV2" etc... in the text and their respective legends should be included in the main text after the legends of regular figures.

- For the figures that you do NOT wish to display as Expanded View figures, they should be bundled together with their legends in a single PDF file called *Appendix*, which should start with a short Table of Content. Appendix figures should be referred to in the main text as: "Appendix Figure S1, Appendix Figure S2" etc. See detailed instructions regarding expanded view here: <https://www.embopress.org/page/journal/14693178/authorguide#expandedview>

- Additional Tables/Datasets should be labeled and referred to as Table EV1, Dataset EV1, etc. Legends have to be provided in a separate tab in case of .xls files. Alternatively, the legend can be supplied as a separate text file (README) and zipped together with the Table/Dataset file.

7) Please note that a Data Availability section at the end of Materials and Methods is now mandatory. In case you have no data that requires deposition in a public database, please state so instead of refereeing to the database.
See also < https://www.embopress.org/page/journal/14693178/authorguide#dataavailability>). Please note that the Data Availability Section is restricted to new primary data that are part of this study.

8) We would also encourage you to include the source data for figure panels that show essential data. Numerical data should be provided as individual .xls or .csv files (including a tab describing the data). For blots or microscopy, uncropped images should be submitted (using a zip archive if multiple images need to be supplied for one panel). Additional information on source data and instruction on how to label the files are available
<https://www.embopress.org/page/journal/14693178/authorguide#sourcedata>.

9) Our journal encourages inclusion of *data citations in the reference list* to directly cite datasets that were re-used and obtained from public databases. Data citations in the article text are distinct from normal bibliographical citations and should directly link to the database records from which the data can be accessed. In the main text, data citations are formatted as follows: "Data ref: Smith et al, 2001" or "Data ref: NCBI Sequence Read Archive PRJNA342805, 2017". In the Reference list, data citations must be labeled with "[DATASET]". A data reference must provide the database name, accession number/identifiers and a resolvable link to the landing page from which the data can be accessed at the end of the reference. Further instructions are available at <https://www.embopress.org/page/journal/14693178/authorguide#referencesformat>.

10) Regarding data quantification
The following points must be specified in each figure legend:
- the name of the statistical test used to generate error bars and P values,
- the number (n) of independent experiments (please specify technical or biological replicates) underlying each data point,
- the nature of the bars and error bars (s.d., s.e.m.)
Discussion of statistical methodology can be reported in the materials and methods section, but figure legends should contain a basic description of n, P and the test applied.
- Please also include scale bars in all microscopy images.

11) As part of the EMBO publication's Transparent Editorial Process, EMBO reports publishes online a Review Process File to accompany accepted manuscripts. This File will be published in conjunction with your paper and will include the referee reports, your point-by-point response and all pertinent correspondence relating to the manuscript.

You are able to opt out of this by letting the editorial office know (emboreports@embo.org). If you do opt out, the Review Process File link will point to the following statement: "No Review Process File is available with this article, as the authors have chosen not to make the review process public in this case."

We would also welcome the submission of cover suggestions, or motifs to be used by our Graphics Illustrator in designing a cover.

I look forward to seeing a revised version of your manuscript when it is ready. Please let me know if you have questions or comments regarding the revision.

Yours sincerely

Martina Rembold, PhD
Senior Editor
EMBO reports

****

Referee #1:

In their revised version the authors have now significantly strengthened their data and addressed many of my concerns.

Nevertheless, to my opinion, there are still some important points that need to be clarified prior to publication.

1) My major concern was the use of a largely uncharacterized mito-pHfluorin reporter for the detection of autophagy. Suppl. Figure 2 now shows co-staining of mito-pHfluorin with COX4. Given that mito-pHfluorin is targeted to the outer membrane, co-localization with a mitochondrial outer membrane protein would be more reliable.

2) Since the mito-Keima reporter system is well established in the mitophagy field, my suggestion was to use this system for detection of mitophagy. Referring to Suppl. Fig. 3C, D, the authors now state in their rebuttal letter that they could confirm the data using mitoKeima. However, these data are missing in the respective Figure.

3) To my opinion, the quantification of "mitophagic punctae" still remains a critical issue. The critera defined in Suppl. Figure 4 illustrate the heterogeneity in number and size of these structures. To strenhthen this point the authors now refer to the data obtained with the mito-Keima system (Suppl. Fig. 3C, D and Suppl. Table 1). So, as mentioned above the missing data in Suppl. Fig. 3C, D are very critical.

Referee #2:

The authors report some interesting links between iron chelation and CHIP levels and links between CHIP levels and SENP3 levels. They also show increased SENP3 increases mitophagy implicating deSUMOylation as an important step in mitophagy. They then ascribe SENP3 activity in mitophagy to Fis1 deSUMOylation. How iron chelation links to CHIP or SENP3 is not clear and how Fis1 fosters mitophagy was not explored. Although of potential importance, a number of issues described below need to be rigorously addressed.

1) At the top of page 6 the authors equate LC3 lipidation in Suppl. Fig. 1 with autophagosome formation. This is incorrect as LAP and other lipidation processes occur in the absence of autophagosomes. Please reword. Also on page 6, Fig 1D shows a western blot not mRNA as stated in the text.

2) Fig. 4B is quite important and shows a quite minimal effect on LC3II in Fis1 KO cells. It is important to perform Fis1 rescue experiments in the Fis1 KO cells and also ascertain if Fis1 OE increases mitophagy. What happens if one adds Bafilomycin to prevent LC3II degradation in such experiments?

3) The model (Fig. 8) is that the loss of SUMOylation promotes Fis1-mediated mitophagy activity. But not much of the total Fis1 is SUMOylated in the absence of DFP (Fig. 5B). If a DFP-induced increase in SENP3 levels decreases further the minor level of SUMO-Fis1, how could such a slight increase in deSUMOylated Fis1 have such big effects on mitophagy? Does OE of SENP3 decrease the small amount of SUMO-Fis1 in Fig. 5B? Does DFP treatment decrease the level of SUMO-Fis1 in Fig. 5B? It seems that endogenous SENP3 does a fine job of deSUMOylating Fis1 (Fig. 5C) and it is not clear that an increase in SENP3 by DFP would make much difference.

4) Is SENP3 involved in Parkin mediated mitophagy? This is particularly interesting because of prior reports of Fis1 involvement in Parkin-mediated mitophagy as noted by the authors.

We thank the ***EMBO Reports*** reviewers for their comments regarding the significant improvement associated with the revised manuscript (EMBOR-2019-48754V2-Q), as well as their further reasonable concerns and constructive advice.

Accordingly, we have now performed further experimental work and additional revision to address all of the points raised. Please find our point-by-point response below. We believe that the manuscript has been further improved as a result. Therefore, we very much hope the further revised manuscript will be accepted for publication in ***EMBO Reports*** in due course.

Referee #1:

In their revised version the authors have now significantly strengthened their data and addressed many of my concerns. Nevertheless, to my opinion, there are still some important points that need to be clarified prior to publication.

We thank the reviewer for her/his positive comment on the data presented in the revised manuscript, and we greatly appreciate all the important points raised.

1) My major concern was the use of a largely uncharacterized mito-pHfluorin reporter for the detection of autophagy. Suppl. Figure 2 now shows co-staining of mito-pHfluorin with COX4. Given that mito-pHfluorin is targeted to the outer membrane, co-localization with a mitochondrial outer membrane protein would be more reliable.

We thank the reviewer for raising this important point. Accordingly, we have now immuocytochemically stained for two mitochondrial outer membrane proteins, Fis1 and Mff, in HeLa cells expressing the mito-pHfluorin. As expected, our new results show co-localization of either protein with the mitophagy reporter (see Fig EV2). It is worthy of note that i) the outlines of mitochondria depicted by the mito-pHfluorin shown in the further revised manuscript share a similar pattern to ones shown in a previous observation using high-resolution confocal microscopy in HeLa cells expressing GFP-ActA (Sengupta et al., 2009); ii) the ActA mitochondrial targeting sequence has been used previously in a reporter for mitophagy detection (Thomas et al., 2018).

2) Since the mito-Keima reporter system is well established in the mitophagy field, my suggestion was to use this system for detection of mitophagy. Referring to Suppl. Fig. 3C, D, the authors now state in their rebuttal letter that they could confirm the data using mitoKeima. However, these data are missing in the respective Figure.

We thank the reviewer for emphasizing this important point. In the meantime, we have to point out that the data using mito-Keima the reviewer is referring to were included in the revised manuscript (EMBOR-2019-48754V2-Q) as Fig 3C and D: the former showing the establishment of HeLa cells stably expressing the mito-Keima, and the latter showing the effect of SENP3 knockdown on changes in numbers of cells displaying the “mitophagic” red puncta induced by DFP treatment in the mito-Keima expressing cells.

3) To my opinion, the quantification of "mitophagic punctae" still remains a critical issue. The critera defined in Suppl. Figure 4 illustrate the heterogeneity in number and size of these structures. To strenhthen this point the authors now refer to the data obtained with the mito-Keima system (Suppl. Fig. 3C, D and Suppl. Table 1). So, as mentioned above the missing data in Suppl. Fig. 3C, D are very critical.

Please see our response to point 2.

Referee #2:

The authors report some interesting links between iron chelation and CHIP levels and links between CHIP levels and SENP3 levels. They also show increased SENP3 increases mitophagy implicating deSUMOylation as an important step in mitophagy. They then ascribe SENP3 activity in mitophagy to Fis1 deSUMOylation. How iron chelation links to CHIP or SENP3 is not clear and how Fis1 fosters mitophagy was not explored. Although of potential importance, a number of issues described below need to be rigorously addressed.

We thank the reviewer for raising the two important new research questions. In our view, these questions, although beyond the scope of this manuscript, will definitely serve as the starting point for our future investigation. Therefore, a few sentences have been included in the Discussion in the further revised manuscript: "In future studies it will be necessary to establish how DFP-mediated iron chelation reduces CHIP gene expression." on page 13; "Moreover, the C-terminus of Fis1 is known to be essential for its interaction with TBC1D15 (Onoue et al., 2013), a Rab GTPase-activating protein (Rab-GAP) required for mitophagic autophagosome formation (Yamano et al., 2014). Therefore, in future it will be necessary to establish if Fis1 SUMOylation status regulates Fis1 binding to TBC1D15 to regulate mitophagy." on page 15.

1) At the top of page 6 the authors equate LC3 lipidation in Suppl. Fig. 1 with autophagosome formation. This is incorrect as LAP and other lipidation processes occur in the absence of autophagosomes. Please reword. Also on page 6, Fig 1D shows a western blot not mRNA as stated in the text.

We thank the reviewer for raising this interesting point. To the best of our knowledge, LC3-II is generally accepted as a marker for autophagy (Klionsky et al., 2021), and so far no published evidence suggests/indicates the occurrence of LC3-associated phagocytosis (LAP) or the induction of LAP by DFP treatment, in HeLa cells. Currently, induction time duration is used as one of the existing criteria to distinguish the two cellular processes: "LC3-decorated autophagosome" formation usually occurs over a period of hours, whilst phagocytosis-induced LAP can occur in as little as 10 minutes (Klionsky et al., 2021). As shown in the following figure, in our hands, LC3-II induction by DFP (50mM) is a temporal process and needs at least 8-12 hours to be detected in HeLa cells.

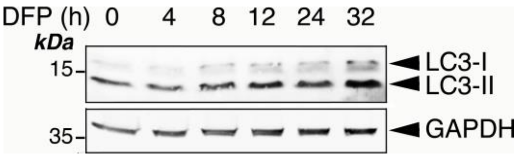

Therefore, we believe that in the present study LC3-II represents a marker for DFP-induced mitophagic autophagosomes. Nevertheless, as the reviewer suggests, we have now replaced "autophagosomes" or "autophagosome formation" with "LC3 lipidation" throughout the text and figure legends in the further revised manuscript.

We thank the reviewer for pointing out our error in figure labelling: Fig 1"E" was mistakenly referred to as Fig 1"D" in the revised manuscript. Accordingly, the correction has been made in the further revised manuscript.

2) Fig. 4B is quite important and shows a quite minimal effect on LC3II in Fis1 KO cells. It is important to perform Fis1 rescue experiments in the Fis1 KO cells and also ascertain if Fis1 OE increases mitophagy. What happens if one adds Bafilomycin to prevent LC3II degradation in such experiments?

We thank the reviewer for raising this important question. We have conducted the rescue experiment as requested. Our new results show that LC3-II becomes inducible in DFP-treated Fis1 KO cells expressing Flag-Fis1 (see new Fig 4C), further substantiating the role of Fis1 in DFP-induced mitophagy. Moreover, our new results indicate that expressing Flag-Fis1 in either WT or Fis1 KO cells does not cause changes in LC3-II levels in the presence of chloroquine (see Appendix Fig S6 and Fig 7A), discounting the possibility that Fis1 overexpression alone results in mitophagy.

3) The model (Fig. 8) is that the loss of SUMOylation promotes Fis1-mediated mitophagy activity. But not much of the total Fis1 is SUMOylated in the absence of DFP (Fig. 5B). If a DFP-induced increase in SENP3 levels decreases further the minor level of SUMO-Fis1, how could such a slight increase in deSUMOylated Fis1 have such big effects on mitophagy? Does OE of SENP3 decrease the small amount of SUMO-Fis1 in Fig. 5B? Does DFP treatment decrease the level of SUMO-Fis1 in Fig. 5B? It seems that endogenous SENP3 does a fine job of deSUMOylating Fis1 (Fig. 5C) and it is not clear that an increase in SENP3 by DFP would make much difference.

We thank the reviewer for raising these important questions.
The first question is intriguing (*i.e.*, "*If a DFP-induced increase in SENP3 levels decreases further the minor level of SUMO-Fis1, how could such a slight increase in deSUMOylated Fis1 have such big effects on mitophagy?"*): Since we do not have a direct answer to this question, we have suggested a potential mechanism based on our findings in this study in the Discussion on pages 14 and 15: "In keeping with the importance of Fis1 mitochondrial localization, our results showing that mutation of the major SUMOylated lysine (K149) enhances its mitochondrial presence indicate that preventing SUMOylation promotes Fis1 mitochondrial localization and suggest that SUMO-2/3 modification may act as a molecular switch to regulate levels of mitochondrial Fis1. However, it should be noted that, in fact, at any given time only a small proportion of Fis1 is SUMOylated. This argues against constitutive SUMOylation being required to maintain Fis1 away from mitochondria. Rather, similar to what we have previously suggested regarding the roles for SUMOylation in Drp1 mitochondrial partitioning (Guo et al., 2013), deSUMOylation may act as a 'mobilisation factor' for Fis1 mitochondrial targeting."

The second question is important (*i.e.*, "*Does OE of SENP3 decrease the small amount of SUMO-Fis1 in Fig. 5B?"*): Our initial experiment for detecting His-SUMO-2-lyation of endogenous Fis1 used HEK293 cells stably expressing His-SUMO-2 (through puromycin selection), as shown in Fig 5B. To better answer this question, we compared the levels of endogenous SUMO-2/3 modification of Fis1 detected in HeLa cells (the model cells used in this study) expressing GFP or GFP-SENP3. As expected, our new results show that levels of SUMO-2/3-ylated Fis1 in GFP-SENP3 expressing cells were lower than those in GFP expressing cells (see Appendix Fig S7).

The third question is important, but challenging to design an experiment to provide an answer to (*i.e.*, "*Does DFP treatment decrease the level of SUMO-Fis1 in Fig. 5B?"*): because DFP treatment leads to Fis1 degradation, as shown by others (Yamano et al., 2014) and us (see Figs 4A, 4B and 4C and Appendix Fig S5), it is difficult to maintain Fis1 levels for demonstrating SENP3-mediated deSUMOlyation of Fis1 in DFP-treated cells. Owing to the fact we do not have direct experimental evidence to answer this question, a few sentences have been included in the Discussion on page 15, "Furthermore, the fact that DFP treatment leads to significant loss of Fis1 in the model cells has made it technically difficult to compare Fis1 SUMOylation levels between DFP-treated cells and their controls. Therefore, in this respect, it will be necessary to develop new experimental tools/assays for demonstrating SENP3-mediated deSUMOylation of Fis1 upon DFP-mediated iron chelation in future."

4) Is SENP3 involved in Parkin mediated mitophagy? This is particularly interesting because of prior reports of Fis1 involvement in Parkin-mediated mitophagy as noted by the authors.

We thank the reviewer for raising this very important and intriguing question. Although currently we do not have experimental data to answer this question, we discuss it on page 14, "Interestingly, we found that increased levels of SENP3 are also inducible using the mitochondrial stressors CCCP and paraquat (Appendix Figs S10 and S11). However, SENP3 knockdown did not seem to have an effect on LC3-II induction by CCCP in HEK293 cells, implying that SENP3 may not be important for mitophagy dependent on Parkin. Nevertheless, since current evidence shows Fis1 involvement in Parkin-dependent mitophagy (Rojansky et al., 2016, Yamano et al., 2014), in future it will be necessary to fully assess the role of SENP3-mediated Fis1 deSUMOlyation in regulating Parkin-dependent mitophagic autolysosome formation using the reporters used in this study."

Reference

Klionsky DJ, Abdel-Aziz AK, Abdelfatah S, Abdellatif M, Abdoli A, Abel S, Abeliovich H, Abildgaard MH, Abudu YP, Acevedo-Arozena A, Adamopoulos IE, Adeli K, Adolph TE, Adornetto A, Aflaki E, Agam G, Agarwal A, Aggarwal BB, Agnello M, Agostinis P et al. (2021) Guidelines for the use and interpretation of assays for monitoring autophagy (4th edition)(1). Autophagy 17: 1-382

Rojansky R, Cha MY, Chan DC (2016) Elimination of paternal mitochondria in mouse embryos occurs through autophagic degradation dependent on PARKIN and MUL1. eLife 5

Sengupta D, Truschel S, Bachert C, Linstedt AD (2009) Organelle tethering by a homotypic PDZ interaction underlies formation of the Golgi membrane network. J Cell Biol 186: 41-55

Thomas HE, Zhang Y, Stefely JA, Veiga SR, Thomas G, Kozma SC, Mercer CA (2018) Mitochondrial Complex I Activity Is Required for Maximal Autophagy. Cell Rep 24: 2404-2417.e8

Yamano K, Fogel AI, Wang C, van der Bliek AM, Youle RJ (2014) Mitochondrial Rab GAPs govern autophagosome biogenesis during mitophagy. eLife 3: e01612

Dear Dr. Guo

Thank you for the submission of your revised manuscript to EMBO reports. I am sorry for the delay in handling your manuscript. We have received the report from former referee 1 (copied below), who is positive about the study and supports publication. Unfortunately, referee 2 was not available to comment further on the revisions and I have therefore asked additional input from an expert advisor on your manuscript. The advisor analysed your revised manuscript and your response to the referee's concerns and concluded that you have addressed most concerns either experimentally or alternatively in the text in an overall convincing manner. The advisor agreed with referee 1 and supported publication of your manuscript in EMBO Reports.

Browsing through the manuscript myself, I noticed a few editorial things that we need before we can proceed with the official acceptance of your study.

- Please note that a Data Availability section at the end of Materials and Methods is now mandatory. In case you have no data that requires deposition in a public database, please state so instead of referring to a database.
See also https://www.embopress.org/page/journal/14693178/authorguide#dataavailability>).

- Figure legends: Please define in all legends whether the replicate experiments (n) refer to technical or biological/independent experiments.

- We noticed that Fig. EV4 is called out after Fig. EV5. You might want to consider changing their order.

- Appendix: Please remove the legends from the manuscript file and only include them in the Appendix pdf.

- I attach to this email a related manuscript file with comments by our data editors. Please address all comments and upload a revised file with tracked changes with your final manuscript submission.

- Source data for Fig. 7C: the LC3-II band in lane 5 misses the middle part, so to say, with the air bubble. Is this from a different exposure?

- Source data for Fig. 4D: The Nsi DFP data set we have as source data looks different to the rest (572x572 pixels in size instead of the 472x472 pixels for the other .czi files. Also the contrast settings differ.) Do you still have the original file for this dataset? The same holds true for GFP-Cb5/Flag-Fis1-SUMO-2 DGG for Fig. 6B.
- We also note that the contrast of the confocal images has been adjusted to reduce what seems background staining. We generally recommend using as little brightness/contrast modification as possible and to comment on image processing in the methods section.

- Finally, EMBO reports papers are accompanied online by A) a short (1-2 sentences) summary of the findings and their significance, B) 2-3 bullet points highlighting key results and C) a synopsis image that is 550x200-600 pixels large (width x height) in .png format. You can either show a model or key data in the synopsis image. Please note that the size is rather small and that text needs to be readable at the final size. Please send us this information along with the revised manuscript.

We look forward to seeing a final version of your manuscript as soon as possible.

With kind regards,

Martina Rembold, PhD
Senior Editor
EMBO reports

****

Referee #1:

The authors have now adequately addressed my criticisms.

Thank you for the submission of your revised manuscript to EMBO reports. I am sorry for the delay in handling your manuscript. We have received the report from former referee 1 (copied below), who is positive about the study and supports publication.
Unfortunately, referee 2 was not available to comment further on the revisions and I have therefore asked additional input from an expert advisor on your manuscript. The advisor analysed your revised manuscript and your response to the referee's concerns and concluded that you have addressed most concerns either experimentally or alternatively in the text in an overall convincing manner. The advisor agreed with referee 1 and supported publication of your manuscript in EMBO Reports.
We thank all the excellent editorial work by ***EMBO Reports*** team, the comment by the referee 1, the analysis and conclusion by the expert advisor, and their support to publish our manuscript in ***EMBO Reports***.

Browsing through the manuscript myself, I noticed a few editorial things that we need before we can proceed with the official acceptance of your study.
We are grateful for Dr Rembold's careful and insightful editorial work. Please find our point-by-point response below.

- Please note that a Data Availability section at the end of Materials and Methods is now mandatory. In case you have no data that requires deposition in a public database, please state so instead of referring to a database. See also https://www.embopress.org/page/journal/14693178/authorguide#dataavailability>).
We thank this helpful instruction. Accordingly, the Data Availability section "This study includes no data deposited in public repositories" has been included in the revised manuscript.

- Figure legends: Please define in all legends whether the replicate experiments (n) refer to technical or biological/independent experiments.
We have included all the details referring to "technical or biological/independent experiments", as required, in the Figure legends with tracked-changes in the revised manuscript.

- We noticed that Fig. EV4 is called out after Fig. EV5. You might want to consider changing their order.
We thank this constructive advice. Now the order of the two figures has been changed in the revised manuscript.

- Appendix: Please remove the legends from the manuscript file and only include them in the Appendix pdf.
We thank this helpful instruction. Now all the legends for the Appendix Figures S1-12 have been removed from the manuscript (text) file and they have been included in the Appendix pdf.

- I attach to this email a related manuscript file with comments by our data editors. Please address all comments and upload a revised file with tracked changes with your final manuscript submission.
We thank this helpful instruction. All the comments from Dr Rembold and EMBO Reports data editors have been addressed and the revised manuscript file with tracked-changes has been submitted.

- Source data for Fig. 7C: the LC3-II band in lane 5 misses the middle part, so to say, with the air bubble. Is this from a different exposure?
We thank Dr Rembold's important question. We have updated the Source data with a blot with the same exposure as the one shown in the Fig.7C.

-Source data for Fig. 4D: The Nsi DFP data set we have as source data looks different to the rest (572x572 pixels in size instead of the 472x472 pixels for the other .czi files. Also the contrast settings differ.) Do you still have the original file for this dataset? The same holds true for GFP-Cb5/Flag-Fis1-SUMO-2 DGG for Fig. 6B.

We thank Dr Rembold for raising this important point. We have taken it very seriously and conducted an investigation. For the Fig. 4D, we found the original file for Nsi DFP (CZI files for Red/Green and Blue channels respectively), where the resolution for the Nsi DFP images is comparable to that for the other microscopic images in the same figure though their total pixels in size are greater than those for the other raw images. Accordingly we have included the original files, and, based on them, revised the Nsi DFP composite figure with appropriate contrast settings and scale bars. As for Fig. 6B, we noticed the differences in the image resolution between the previous raw data for GFP-Cb5/Flag-Fis1-SUMO-2 DGG and the raw data for other images in the same figure. Therefore, we have included new raw data for GFP-Cb5/Flag-Fis1-SUMO-2 DGG, and based on them, re-prepared a composite figure showing the overlap between green (GFP-Cb5) channel and blue (Flag-Fis1-SUMO-2 DGG) channel.

-We also note that the contrast of the confocal images has been adjusted to reduce what seems background staining. We generally recommend using as little brightness/contrast modification as possible and to comment on image processing in the methods section.

We thank EMBO Reports team for this very important recommendation. Accordingly, we have checked all our imaging results/data presented in this manuscript, and commented in the methods section as follows:
"Adjustments of Brightness and contrast using Fjji ImageJ (https://imagej.net/software/fiji/) were applied to each individual confocal image as a whole, as little as possible, and under conditions the adjustments do not alter any information contained in the original image."

-Finally, EMBO reports papers are accompanied online by A) a short (1-2 sentences) summary of the findings and their significance, B) 2-3 bullet points highlighting key results and C) a synopsis image that is 550x200-600 pixels large (width x height) in .png format. You can either show a model or key data in the synopsis image. Please note that the size is rather small and that text needs to be readable at the final size. Please send us this information along with the revised manuscript.

We thank this helpful instruction. Accordingly we have prepared a short summary and bullet pointed sentences as well as a synopsis image (two versions with different pixel dimensions) as required along with our revised manuscript. Please feel free to let us know if they need to be modified/revised.

Referee #1:

The authors have now adequately addressed my criticisms.

We thank the Referee #1 for all her/his comments and advice on our manuscript.

Dr. Chun Guo
University of Sheffield
School of Biosciences
B2.221a Alfred Denny building
Firth Court, Western Bank
Sheffield, South Yorkshire S10 2TN
United Kingdom

Dear Dr. Guo,

I am very pleased to accept your manuscript for publication in the next available issue of EMBO reports. Thank you for your contribution to our journal.

At the end of this email I include important information about how to proceed. Please ensure that you take the time to read the information and complete and return the necessary forms to allow us to publish your manuscript as quickly as possible.

As part of the EMBO publication's Transparent Editorial Process, EMBO reports publishes online a Review Process File to accompany accepted manuscripts. As you are aware, this File will be published in conjunction with your paper and will include the referee reports, your point-by-point response and all pertinent correspondence relating to the manuscript.

If you do NOT want this File to be published, please inform the editorial office within 2 days, if you have not done so already, otherwise the File will be published by default [contact: emboreports@embo.org]. If you do opt out, the Review Process File link will point to the following statement: "No Review Process File is available with this article, as the authors have chosen not to make the review process public in this case." Please note that the author checklist will still be published even if you opt out of the transparent process.

Thank you again for your contribution to EMBO reports and congratulations on a successful publication. Please consider us again in the future for your most exciting work.

Kind regards,

Martina Rembold, PhD
Senior Editor
EMBO reports

****

THINGS TO DO NOW:

You will receive proofs by e-mail approximately 2-3 weeks after all relevant files have been sent to our Production Office; you should return your corrections within 2 days of receiving the proofs.

Please inform us if there is likely to be any difficulty in reaching you at the above address at that time. Failure to meet our deadlines may result in a delay of publication, or publication without your corrections.

All further communications concerning your paper should quote reference number EMBOR-2019-48754V4 and be addressed to emboreports@wiley.com.

Should you be planning a Press Release on your article, please get in contact with emboreports@wiley.com as early as possible, in order to coordinate publication and release dates.

EMBO PRESS

YOU MUST COMPLETE ALL CELLS WITH A PINK BACKGROUND ➔

PLEASE NOTE THAT THIS CHECKLIST WILL BE PUBLISHED ALONGSIDE YOUR PAPER

Corresponding Author Name: Chun Guo

Journal Submitted to: EMBO Reports

Manuscript Number: EMBOR-2019-48754V3

Reporting Checklist For Life Sciences Articles (Rev. June 2017)

This checklist is used to ensure good reporting standards and to improve the reproducibility of published results. These guidelines are consistent with the Principles and Guidelines for Reporting Preclinical Research issued by the NIH in 2014. Please follow the journal's authorship guidelines in preparing your manuscript.

A- Figures

1. Data

The data shown in figures should satisfy the following conditions:

- the data were obtained and processed according to the field's best practice and are presented to reflect the results of the experiments in an accurate and unbiased manner.
- figure panels include only data points, measurements or observations that can be compared to each other in a scientifically meaningful way.
- graphs include clearly labeled error bars for independent experiments and sample sizes. Unless justified, error bars should not be shown for technical replicates.
- if n< 5, the individual data points from each experiment should be plotted and any statistical test employed should be justified
- Source Data should be included to report the data underlying graphs. Please follow the guidelines set out in the author ship guidelines on Data Presentation.

2. Captions

Each figure caption should contain the following information, for each panel where they are relevant:

- a specification of the experimental system investigated (eg cell line, species name).
- the assay(s) and method(s) used to carry out the reported observations and measurements
- an explicit mention of the biological and chemical entity(ies) that are being measured.
- an explicit mention of the biological and chemical entity(ies) that are altered/varied/perturbed in a controlled manner.

- the exact sample size (n) for each experimental group/condition, given as a number, not a range;
- a description of the sample collection allowing the reader to understand whether the samples represent technical or biological replicates (including how many animals, litters, cultures, etc.).
- a statement of how many times the experiment shown was independently replicated in the laboratory.
- definitions of statistical methods and measures:
  - common tests, such as t-test (please specify whether paired vs. unpaired), simple χ2 tests, Wilcoxon and Mann-Whitney tests, can be unambiguously identified by name only, but more complex techniques should be described in the methods section;
  - are tests one-sided or two-sided?
  - are there adjustments for multiple comparisons?
  - exact statistical test results, e.g., P values = x but not P values < x;
  - definition of 'center values' as median or average;
  - definition of error bars as s.d. or s.e.m.

Any descriptions too long for the figure legend should be included in the methods section and/or with the source data.

In the pink boxes below, please ensure that the answers to the following questions are reported in the manuscript itself. Every question should be answered. If the question is not relevant to your research, please write NA (non applicable). We encourage you to include a specific subsection in the methods section for statistics, reagents, animal models and human subjects.

USEFUL LINKS FOR COMPLETING THIS FORM

http://www.antibodypedia.com	Antibodypedia
http://1degreebio.org	1DegreeBio
http://www.equator-network.org/reporting-guidelines/improving-bioscience-research-repor	ARRIVE Guidelines
http://grants.nih.gov/grants/olaw/olaw.htm	NIH Guidelines in animal use
http://www.mrc.ac.uk/Ourresearch/Ethicsresearchguidance/Useofanimals/index.htm	MRC Guidelines on animal use
http://ClinicalTrials.gov	Clinical Trial registration
http://www.consort-statement.org	CONSORT Flow Diagram
http://www.consort-statement.org/checklists/view/32-consort/66-title	CONSORT Check List
http://www.equator-network.org/reporting-guidelines/reporting-recommendations-for-tumo	REMARK Reporting Guidelines (marker prognostic studies)
http://datadryad.org	Dryad
http://figshare.com	Figshare
http://www.ncbi.nlm.nih.gov/gap	dbGAP
http://www.ebi.ac.uk/ega	EGA
http://biomodels.net/	Biomodels Database
http://biomodels.net/miriam/	MIRIAM Guidelines
http://jjj.biochem.sun.ac.za	JWS Online
https://osp.od.nih.gov/biosafety-biosecurity-and-emerging-biotechnology/	Biosecurity Documents from NIH
http://www.selectagents.gov/	List of Select Agents

B- Statistics and general methods

	Please fill out these boxes ➔ (Do not worry if you cannot see all your text once you press return)
1.a. How was the sample size chosen to ensure adequate power to detect a pre-specified effect size?	Undoubtedly with larger sample size we have more power to detect a pre-specified effect size if it exists. To detect small differences (a type of pre-specified effect size) a large sample size is required. In this study, in reference to the similar experiments described in the literature, to detect big differences (changes) in terms of either protein/mRNA levels or fluorescent colouration between groups under different well-controlled experimental conditions, relatively small sample sizes were chosen through considering the trade-offs among sample size, power and the pre-specified effect size, as well as consideration of experimental time and expense. Briefly, in biochemical experiments involving the use of immunoblotting/quantitative real-time PCR, sample size was = or >3 whilst in imaging experiments involving the use of microscopy data, sample sizes were >20 and > 360 for cells transfected with mito-pHfluorin and cells stably expressing mito-
1.b. For animal studies, include a statement about sample size estimate even if no statistical methods were used.	N/A
2. Describe inclusion/exclusion criteria if samples or animals were excluded from the analysis. Were the criteria pre-established?	Yes, criteria were pre-established. Inclusion criteria: 1) samples were prepared from specified cell types; 2) the specified cells were from an authenticated Depository Authority (e.g., ATCC and ECACC); 2) the specified cells were cultured under sterile and appropriate conditions; 3) the specified cells were free from contaminations; 4) the specified cells were treated and harvested strictly and precisely according to pre-established experimental procedures. Exclusion criteria: 1) samples were NOT prepared from specified cell types; 2) the specified cells were NOT from an authenticated Depository Authority (e.g., ATCC and ECACC); 2) the specified cells were NOT cultured under sterile and appropriate conditions; 3) the specified cells were NOT free from contaminations; 4) the specified cells were NOT treated and NOT harvested strictly and precisely according to pre-established
3. Were any steps taken to minimize the effects of subjective bias when allocating animals/samples to treatment (e.g. randomization procedure)? If yes, please describe.	Yes, for all the experiments involving transfections/treatments in this study, two steps were taken to minimize the effects of subjective bias when preparing cells for treatment: 1) an aliquot (equal volume) from one common stock of cell suspension was randomly plated cells into individual wells of different plates or individual imaging dishes; 2) upon treatment, cell-containing wells in the plates or dishes were randomly allociated to be treated with a vehicle or a drug/reagent.
For animal studies, include a statement about randomization even if no randomization was used.	N/A
4.a. Were any steps taken to minimize the effects of subjective bias during group allocation or/and when assessing results (e.g. blinding of the investigator)? If yes please describe.	Yes, multiple steps were in place to minimize the effects of subjective bias for some imaging experiments involving statistical comparisons between groups, as described in the manuscript: 1) researcher A randomly plated cells into individual plate wells or imaging dishes; 2) researcher A transfected/treated/harvested cells strictly according to experimental design; 3) researcher A re-labelled the samples in alphabetical order and passed them to researcher B; 4) researcher B captured microscopy images and conducted analysis without knowing sample identities; 5) researcher A finally revealed the sample identities to researcher B.
4.b. For animal studies, include a statement about blinding even if no blinding was done	N/A
5. For every figure, are statistical tests justified as appropriate?	Yes
Do the data meet the assumptions of the tests (e.g., normal distribution)? Describe any methods used to assess it.	Yes, Shapiro-Wilk normality test was used.

Is there an estimate of variation within each group of data?	Yes
Is the variance similar between the groups that are being statistically compared?	Yes

C- Reagents

6. To show that antibodies were profiled for use in the system under study (assay and species), provide a citation, catalog number and/or clone number, supplementary information or reference to an antibody validation profile. e.g., Antibodypedia (see link list at top right), 1DegreeBio (see link list at top right).	Monoclonal β-Actin antibody: https://www.sigmaaldrich.com/GB/en/product/sigma/a5441?context=product; CHIP antibody: https://www.antibodypedia.com/gene/22808/STUB1/antibody/105517/2080; Drp1 antibody: https://www.cellsignal.co.uk/products/primary-antibodies/drp1-d6c7-rabbit-mab/8570; Fis1 Antibod: https://www.antibodypedia.com/gene/2313/FIS1/antibody/152470/10956-1-AP; Flag Antibody: https://www.ptglab.com/products/Flag-tag-Antibody-66008-3-Ig.htm; FTH1 antibody: https://www.antibodypedia.com/gene/28385/FTH1/antibody/611565/4393; GAPDH antibody: https://www.antibodypedia.com/gene/3923/GAPDH/antibody/12717/sc-47724; GST antibody: https://www.labome.com/product/GE-Healthcare-Life-Biosciences/27-4577-01.html; LC3 antibody: https://www.cellsignal.co.uk/products/primary-antibodies/lc3a-b-antibody/4108?site-search-type=Products&N=4294956287&Ntt=lc3&fromPage=plp; p62 antibody: https://www.cellsignal.co.uk/products/primary-antibodies/sqstm1-p62-antibody/5114?site-search-type=Products&N=4294956287&Ntt=p62&fromPage=plp; SENP3 antibody: https://www.antibodypedia.com/gene/24161/SENP3/antibody/167214/5591; SENP5 antibody: https://www.antibodypedia.com/gene/33951/SENP5/antibody/157440/19529-1-AP; SUMO-1 antibody: https://www.scbt.com/p/sumo-1-antibody-d-11; SUMO-2/3 antibodies: https://www.cellsignal.co.uk/products/primary-antibodies/sumo-2-3-18h8-rabbit-mab/4971, https://www.mblintl.com/products/m114-3/; SUMO-2/3 antibody affinity beads (ASM24): https://www.antibodypedia.com/gene/24332/SUMO3/antibody/2109018/ASM24; Tom20 antibody:
7. Identify the source of cell lines and report if they were recently authenticated (e.g., by STR profiling) and tested for mycoplasma contamination.	HeLa cells: https://www.atcc.org/products/ccl-2; HEK293 cells: https://www.phe-culturecollections.org.uk/products/celllines/generalcell/detail.jsp?refId=85120602&collection=ecacc_gc; SH-SY5Y cells: https://www.phe-culturecollections.org.uk/products/celllines/generalcell/detail.jsp?refId=94030304&collection=ecacc_gc

* for all hyperlinks, please see the table at the top right of the document

D- Animal Models

8. Report species, strain, gender, age of animals and genetic modification status where applicable. Please detail housing and husbandry conditions and the source of animals.	N/A
9. For experiments involving live vertebrates, include a statement of compliance with ethical regulations and identify the committee(s) approving the experiments.	N/A
10. We recommend consulting the ARRIVE guidelines (see link list at top right) (PLoS Biol. 8(6), e1000412, 2010) to ensure that other relevant aspects of animal studies are adequately reported. See author guidelines, under 'Reporting Guidelines'. See also: NIH (see link list at top right) and MRC (see link list at top right) recommendations. Please confirm compliance.	N/A

E- Human Subjects

11. Identify the committee(s) approving the study protocol.	N/A
12. Include a statement confirming that informed consent was obtained from all subjects and that the experiments conformed to the principles set out in the WMA Declaration of Helsinki and the Department of Health and Human Services Belmont Report.	N/A
13. For publication of patient photos, include a statement confirming that consent to publish was obtained.	N/A
14. Report any restrictions on the availability (and/or on the use) of human data or samples.	N/A
15. Report the clinical trial registration number (at ClinicalTrials.gov or equivalent), where applicable.	N/A
16. For phase II and III randomized controlled trials, please refer to the CONSORT flow diagram (see link list at top right) and submit the CONSORT checklist (see link list at top right) with your submission. See author guidelines, under 'Reporting Guidelines'. Please confirm you have submitted this list.	N/A
17. For tumor marker prognostic studies, we recommend that you follow the REMARK reporting guidelines (see link list at top right). See author guidelines, under 'Reporting Guidelines'. Please confirm you have followed these guidelines.	N/A

F- Data Accessibility

18: Provide a "Data Availability" section at the end of the Materials & Methods, listing the accession codes for data generated in this study and deposited in a public database (e.g. RNA-Seq data: Gene Expression Omnibus GSE39462, Proteomics data: PRIDE PXD000208 etc.) Please refer to our author guidelines for 'Data Deposition'.<br><br>Data deposition in a public repository is mandatory for:<br>a. Protein, DNA and RNA sequences<br>b. Macromolecular structures<br>c. Crystallographic data for small molecules<br>d. Functional genomics data<br>e. Proteomics and molecular interactions	This study includes no data deposited in external repositories.
19. Deposition is strongly recommended for any datasets that are central and integral to the study; please consider the journal's data policy. If no structured public repository exists for a given data type, we encourage the provision of datasets in the manuscript as a Supplementary Document (see author guidelines under 'Expanded View' or in unstructured repositories such as Dryad (see link list at top right) or Figshare (see link list at top right).	N/A
20. Access to human clinical and genomic datasets should be provided with as few restrictions as possible while respecting ethical obligations to the patients and relevant medical and legal issues. If practically possible and compatible with the individual consent agreement used in the study, such data should be deposited in one of the major public access-controlled repositories such as dbGAP (see link list at top right) or EGA (see link list at top right).	N/A
21. Computational models that are central and integral to a study should be shared without restrictions and provided in a machine-readable form. The relevant accession numbers or links should be provided. When possible, standardized format (SBML, CellML) should be used instead of scripts (e.g. MATLAB). Authors are strongly encouraged to follow the MIRIAM guidelines (see link list at top right) and deposit their model in a public database such as Biomodels (see link list at top right) or JWS Online (see link list at top right). If computer source code is provided with the paper, it should be deposited in a public repository or included in supplementary information.	N/A

G- Dual use research of concern

22. Could your study fall under dual use research restrictions? Please check biosecurity documents (see link list at top right) and list of select agents and toxins (APHIS/CDC) (see link list at top right). According to our biosecurity guidelines, provide a statement only if it could.	No
